# 2023 Chinese guideline for lipid management

**DOI:** 10.3389/fphar.2023.1190934

**Published:** 2023-08-29

**Authors:** Jian-Jun Li, Shui-Ping Zhao, Dong Zhao, Guo-Ping Lu, Dao-Quan Peng, Jing Liu, Zhen-Yue Chen, Yuan-Lin Guo, Na-Qiong Wu, Sheng-Kai Yan, Zeng-Wu Wang, Run-Lin Gao

**Affiliations:** ^1^ National Center for Cardiovascular Diseases, FuWai Hospital, Chinese Academy of Medical Sciences, Beijing, China; ^2^ The Second Xiangya Hospital of Central South University, Changsha, Hunan, China; ^3^ Beijing Anzhen Hospital, Capital Medical University, Beijing, China; ^4^ Ruijin Hospital, Shanghai Jiaotong University School of Medicine, Shanghai, China; ^5^ Affiliated Hospital of Zunyi Medical University, School of Laboratory Medicine of Zunyi Medical University, Zunyi, Guizhou, China

**Keywords:** atherosclerosis, cardiovascular disease, lipid, management, statin, combined therapy

## Abstract

Atherosclerotic cardiovascular disease (ASCVD) is the leading cause of death among urban and rural residents in China, and elevated low-density lipoprotein cholesterol (LDL-C) is a risk factor for ASCVD. Considering the increasing burden of ASCVD, lipid management is of the utmost importance. In recent years, research on blood lipids has made breakthroughs around the world, hence a revision of Chinese guideline for lipid management is imperative, especially since the target lipid levels in the general population vary in respect to the risk of ASCVD. The level of LDL-C, which can be regarded as appropriate in a population without frisk factors, can be considered abnormal in people at high risk of developing ASCVD. As a result, the “Guidelines for the prevention and treatment of dyslipidemia" were adapted into the “Chinese guideline for Lipid Management" (henceforth referred to as the new guidelines) by an Experts’ committee after careful deliberation. The new guidelines still recommend LDL-C as the primary target for lipid control, with cardiovascular disease (CVD) risk stratification to determine its target value. These guidelines recommend that moderate intensity statin therapy in adjunct with a heart-healthy lifestyle, be used as an initial line of treatment, followed by cholesterol absorption inhibitors or/and proprotein convertase subtilisin/kexin type 9 (PCSK9) inhibitors, as necessary. The new guidelines provide guidance for lipid management across various age groups, from children to the elderly. The aim of these guidelines is to comprehensively improve the management of lipids and promote the prevention and treatment of ASCVD by guiding clinical practice.

## 1 Introduction

Cardiovascular disease (CVD) is the most common chronic non-communicable disease threatening human life and health globally. CVD, and especially atherosclerotic cardiovascular disease (ASCVD) (e.g., ischemic heart disease and ischemic stroke), is the leading cause of death among urban and rural residents in China, accounting for more than 40% of the deaths (National Center for Cardiovascular Diseases, 2022). Cardiovascular Disease (CVD) refers to a class of diseases that affect the heart and blood vessels. It encompasses various conditions such as coronary artery disease, heart failure, arrhythmias, valvular heart diseases, and peripheral artery disease. The component events of CVD can vary depending on the specific condition but may include coronary artery blockage, heart attack, heart failure, arrhythmias (abnormal heart rhythms), and problems with heart valves or the blood vessels. Atherosclerotic Cardiovascular Disease (ASCVD): ASCVD specifically refers to a subset of cardiovascular diseases caused by the buildup of plaque in the arteries, leading to atherosclerosis. ASCVD primarily involves conditions like coronary artery disease, cerebrovascular disease (stroke), and peripheral artery disease. The primary component events of ASCVD are related to the development and consequences of plaque buildup in the arteries. These include coronary artery blockage leading to angina or heart attack, carotid artery disease resulting in stroke, and peripheral artery disease leading to reduced blood flow to the extremities. In recent years, the disease burden of ASCVD in China has continued to increase (Zhao et al., 2019), and the situation of prevention and control is severe. Hence, there is an urgent need to improve the measures of prevention and treatment of ASCVD.

There is strong evidence from epidemiological, genetic, and clinical interventional studies confirming that low-density lipoprotein cholesterol (LDL-C) is a causal risk factor for ASCVD (Ference et al., 2017). Recent studies also suggest that apolipoprotein B (ApoB) containing lipoproteins, including triglyceride-rich lipoproteins (TRL) and their residues, as well as lipoprotein a) [Lp(a)], are not only involved in the pathophysiology of ASCVD but may also be associated with events such as atherosclerotic thrombosis (Ference et al., 2017).

The age-adjusted coronary heart disease mortality rate in the United States in the 20th century showed the start of a declining trend in 1968 and decreased by more than 40% between 1980 and 2000. The contribution of controlling the risk factors accounted for 44%, and the largest contributor was the reduction of total cholesterol (TC) levels which accounted for 24% (Ford et al., 2007). To the contrary, data show that the TC, LDL-C, and triglyceride (TG) levels of Chinese residents have increased significantly in 2012 compared to 2002, while high-density lipoprotein cholesterol (HDL-C) decreased significantly, and the prevalence of dyslipidemia in people aged ≥18 years has increased significantly (Center for Disease Control and Prevention of National Health Commission of the People’s Republic of China, 2015). The awareness -, treatment - and control rate of dyslipidemia among residents are at a low level during the same time periods. As a result, China is facing an increasing trend of ASCVD disease burden, and lipid management is urgent.

In 2007, the Chinese Society of Cardiology, with the support of the former Center for Disease Control and Prevention of Ministry of Health of the People’s Republic of China, organized a joint committee of multidisciplinary Experts to develop the *Chinese guidelines on Prevention and Treatment of Dyslipidemia in Adults* (Joint Committee on Chinese Guidelines on Prevention and Treatment of Dyslipidemia in Adults, 2007) based on the 1997 *Recommendations for Prevention and Treatment of Dyslipidemia* (Chinese Cardiovascular Journal Editorial Board Dyslipidemia Prevention and Treatment Countermeasures Special Group, 1997). In 2016, the National Experts’ Committee for Cardiovascular Diseases of the National Center for Cardiovascular Disease, in collaboration with the former Center for Disease Control and Prevention of National Health and Family Planning Commission of the People’s Republic of China, organized a multidisciplinary Expert committee. The objective of the committee was to document the progress of national and international research in the field of dyslipidemia, focusing on epidemiological and clinical research evidence in China, and comprehensively integrate it with the international dyslipidemia guidelines, to formulate the *2016 Chinese guideline for the Management of Dyslipidemia in Adults* (Joint Committee on Chinese Guideline for the Management of Dyslipidemia in Adults, 2016). This guideline put forward more suitable dyslipidemia management recommendations for the Chinese population and was pivotal in guiding the prevention and treatment of dyslipidemia, including at the grassroots level (National Society of Cardiometabolic Medicine, 2022).

Following the publication of the *2016 Chinese guideline for the Management of Dyslipidemia in Adults*, the causal relationship between LDL-C and atherosclerosis was established. Research also demonstrated that the combination of lipid-lowering drugs and use of new drug classes such as proprotein convertase subtilisin/kexin type 9 (PCSK9) inhibitors can reduce LDL-C levels by 50%–70%, resulting in further reduction in major adverse cardiovascular events (MACE) based on statin therapy. This observation reaffirmed that additional substantial LDL-C reductions can lead to greater cardiovascular protection (Sabatine et al., 2017; Bhatt et al., 2019). These new clinical trial results have led to updates and revisions of many foreign lipid management guidelines (Grundy et al., 2019; Mach et al., 2020), which tend to emphasize more stringent LDL-C control goals, especially for patients at high risk for ASCVD. Additionally, significant progress has been made researching the residual cardiovascular risk associated with lipids.

Based on the above background, the National Cardiovascular Disease Expert Committee, together with the Chinese Society of Cardiology, the Chinese Society of Endocrinology, the Chinese Diabetes Society, the Chinese Society of Laboratory Medicine and the Chinese Stroke Association, formed a joint Expert committee of multidisciplinary Experts to update the *2016 Chinese guideline for the Management of Dyslipidemia in Adults*. The aim of this committee was to guide clinical practice to comprehensively improve the level of lipid management in China by promoting CVD prevention and treatment.

Considering that the target levels of lipids in the population varies with the level of ASCVD risk stratification, the LDL-C level, which can be regarded as “normal” in a population without risk factors, may be considered significantly “elevated” in patients with ultra (very) high risk of ASCVD. Therefore, after careful and extensive deliberations, the Experts’ committee on guideline revision decided to revise the “Guidelines for the Management of Dyslipidemia” to “Guidelines for Lipid Management”. In addition, as dyslipidemia and atherosclerosis may have onset in childhood, lipid management should also be initiated in children and young adults. This guideline includes recommendations for lipid management in children as well as for patients in various age groups. Therefore, the Experts’ committee unanimously agreed to rename the newly revised guideline as *2023 Chinese guideline for lipid management*.

This guideline revision process followed the methodology and procedures as outlined by the World Health Organization and the Chinese Medical Association for guideline development/revision (Jiang, Z et al., 2016). Firstly, the main contents and core issues of the new guideline were solicited from the Expert committee members, and a total of 15 core issues in 6 areas (1. General principles of guideline revision, 2. Main contents of update, 3. Overall cardiovascular risk assessment, 4. Goals of lipid-lowering therapy, 5. Pharmacological and non-pharmacological treatment measures for lipid-lowering therapy, and 6. Lipid management in children and special populations) were identified after research and ranked. The guideline revision working group developed a literature search and evaluation strategy based on the core issues. The results from the comprehensive search of Chinese and English literature databases were then provided to the Experts to conduct a systematic review and evaluation, paying special attention to the collection and adoption of results and data from national clinical studies and population-based cohort studies. During the preparation process, the Expert’s committee held seven symposiums to discuss the core issues. Based upon the systematic literature review, the Experts’ committee reached consensus after repeated discussion sessions, made recommendations, and assigned levels of evidence. In the instances where there was difference of opinion after repeated discussions, the opinion supported by majority of the experts was accepted.

The definition of levels of recommendation classifications and levels of evidence in this guideline are based on corresponding lipid management guidelines from Europe and the United States (Grundy et al., 2019; Mach et al., 2020).

The definitions of the recommended classifications in this guide are expressed as follows.

Class Ⅰ: Treatments or operations for which there is proven and/or consistent recognition of benefits, usefulness, or effectiveness are recommended.
Class Ⅱ: Treatments or operations for which the evidence of usefulness and/or effectiveness is still contradictory or for which there are different opinions.
Class Ⅱa: Evidence, opinions that tend to be useful and/or effective, and the application of these treatments or operations is justified.
Class Ⅱb: The relevant evidence and views are not yet adequate to prove useful and/or effective, but the application can be considered.
Class Ⅲ: Treatments or operations that have been proven and/or unanimously recognized as ineffective and may be unsafe in some cases are not recommended.

Levels of evidence are defined as follows in the guideline.

Evidence level A: Evidence based on multiple randomized clinical trials (RCT) or meta-analyses.
Evidence level B: Evidence based on a single RCT or multiple non-randomized controlled studies.
Evidence level C: Expert consensus opinion only and/or based on small size studies, retrospective studies, and registry study results.

Declaration of conflict of interest: all participants in the guideline revision declared no conflict of interest. Highlights of the new 2023 guidelines updates compared to the 2016 version are listed in Supplementary Table S2.

## 2 Epidemic characteristics of dyslipidemia

Key points.

1. In recent decades, the lipid levels and the prevalence of dyslipidemia in the Chinese population has increased significantly. The increase in hypercholesterolemia is most pronounced.
2. The treatment rate and achieved rate of lipid-lowering in the ASCVD ultra (very) high risk population are relatively low and need urgent improvement.

Since the 1980s, the Chinese population, including children and adolescents, has experienced a significant change in the lipid levels and a marked increase in the prevalence of dyslipidemia (Yang et al., 2012; Ding et al., 2016; Zhang et al., 2018; Song et al., 2019; Center for Disease Control and Prevention of National Health Commission of the People’s Republic of China, 2020; NCD Risk Factor Collaboration, 2020).

The average level of lipid components is an important indicator to evaluate the trend of lipid changes in the population. Data from the 2018 national survey (Center for Disease Control and Prevention of National Health Commission of the People’s Republic of China, 2020) showed that for Chinese adults aged ≥18 years, the average serum TC was 4.8 mmol/L, LDL-C was 2.9 mmol/L, and TG was 1.7 mmol/L. Compared with the data obtained from the national surveys conducted in 2002, 2010, and 2015 respectively, the average levels of all lipid components were significantly higher (Yang et al., 2012; Song et al., 2019). A recently published global study (NCD Risk Factor Collaboration, 2020) reported that in 1980, the average levels of TC and non-high-density lipoprotein cholesterol (non-HDL-C) in Chinese adults were in a relatively low tier and significantly lower than those in Western countries; whereas in 2018, the average levels of TC and non-HDL-C in Chinese adults were equivalent to - or exceeded the average levels in certain Western countries. Meanwhile, lipid levels in children and adolescents also showed an elevated trend. A study on child and adolescent metabolic syndrome in Beijing (Ding et al., 2016) demonstrated that the average serum TC, LDL-C, and non-HDL-C levels of children and adolescents aged 6–18 years in 2014 were 4.3, 2.4, and 2.8 mmol/L respectively, which were significantly higher than those reported10 years ago. Elevated level of serum cholesterol is expected to lead to an increase of approximately 9.2 million cardiovascular events in China between 2010 and 2030 (Moran et al., 2010). Mitigating this continued rise in the mean serum cholesterol level is an important goal for ASCVD prevention in China.

The prevalence of adult dyslipidemia in China has remained high in recent years (Pan et al., 2016; Song et al., 2019; Center for Disease Control and Prevention of National Health Commission of the People’s Republic of China, 2020). The results of the 2018 national survey demonstrated that the prevalence of dyslipidemia among adults aged ≥18 years was 35.6% (Center for Disease Control and Prevention of National Health Commission of the People’s Republic of China, 2020), which was higher compared with the 2015 national survey (Song et al., 2019). The increase in prevalence of hypertriglyceridemia (TC ≥ 6.2 mmol/L) was the most marked (Center for Disease Control and Prevention of National Health Commission of the People’s Republic of China, 2020). Compared with 2015, the age-specific prevalence of hypertriglyceridemia nearly doubled in 2018 (from 4.9% to 8.2%). The prevalence of high LDL-C also continued to increase, with 8.0% of adults aged ≥18 years with LDL-C ≥4.1 mmol/L in 2018 compared with 5.6% and 7.2% in 2010 and 2015 respectively (Song et al., 2019; Center for Disease Control and Prevention of National Health Commission of the People’s Republic of China, 2020). The prevalence of hypertriglyceridemia also significantly increased in children and adolescents in China during this time period (Ding et al., 2015). The 2012 national survey in children and adolescents aged 6–17 years in 7 provinces, autonomous regions and municipalities directly under the central government (Wang et al., 2018) showed that 5.4% of children and adolescents had hypertriglyceridemia (TC > 5.2 mmol/L), which was about 1.5 times higher than 10 years ago, whilst high TG and low HDL-C were more common in the children.

Improving awareness -, treatment - and control rate of dyslipidemia among the public or ASCVD patients is the core strategy for the primary and secondary prevention of ASCVD. A survey conducted between 2012 and 2015 showed that the awareness rate of dyslipidemia among Chinese adults aged ≥35 years was only 16.1% (China Hypertension Survey and Research Group, 2019). For the high-risk cardiovascular population and ASCVD patients, the goal is to focus on improving the treatment rate of cholesterol-lowering drugs and the target LDL-C rate. The treatment rate of lipid-lowering drugs was only 5.5% of the population at high risk for ASCVD in primary prevention. Of those who already had ASCVD, the treatment rate of lipid-lowering drugs was 14.5% and the target LDL-C rate was only 6.8% (Zhang et al., 2018). In addition, among 104,516 patients hospitalized with acute coronary syndrome (ACS) in 246 hospitals across China, analysis using the criteria of *The Chinese Expert Consensus on Lipid Management of Very High-risk Atherosclerotic Cardiovascular Disease Patients* (Atherosclerosis and Coronary Heart Disease Working Group of Chinese Society of Cardiology and Editorial Board of Chinese Journal of Cardiology, 2020) showed that 75.1% of these patients were at very-high risk, and the target LDL-C rate at admission (<1.4 mmol/L) was only 6.6%; 95.1% of patients (those with discharge prescription information) received only statin monotherapy at discharge (Zeng et al., 2020). A recent follow-up study of 9,944 ASCVD patients including chronic coronary heart disease, ischemic stroke and peripheral vascular disease suggested that 26% of Chinese ASCVD patients were at very-high risk and the target LDL-C rate was only 13% (Li et al., 2021). These results indicate that lipid management in the Chinese population needs to be further strengthened.

## 3 Lipids and lipoproteins

Key points.

1. The main clinically relevant lipid components include cholesterol and TG.
2. Blood cholesterol and TG are mainly found in lipoproteins, including chylomicron (CM), very low-density lipoprotein (VLDL), intermediate-density lipoprotein (IDL), low-density lipoprotein (LDL), High-density Lipoprotein (HDL) and Lipoprotein a) (Lp(a)).

Blood lipids is a general term encompassing serum cholesterol, TG and other lipids (e.g., phospholipids), with the clinically most relevant lipids being cholesterol and TG. Lipids are insoluble in water and must be bound to a special protein, apolipoprotein (Apo), to form lipoproteins before they can be dissolved in the blood and transported to tissues for metabolism.

Lipoproteins are classified as chylomicron (CM), very low-density lipoprotein (VLDL), intermediate-density lipoprotein (IDL), low-density lipoprotein (LDL) and high-density lipoprotein (HDL). In addition, there is another type of lipoprotein called lipoprotein (a) [Lp(a)]. The physical features, main components, sources, and functions of lipoproteins are shown in Supplementary Table S3.

### 3.1 Chylomicron (CM)

CM is synthesized by the small intestine and is the largest lipoprotein in the blood, with the lowest density and the main component being TG. When blood is collected after 12-h fasting in normal people, there is no CM in the serum, but when there is a large amount of CM in the blood after a meal or in certain pathological conditions, the blood has a white cloudy appearance and is called “Chylomicronemia”.

### 3.2 Very low-density lipoprotein (VLDL)

VLDL is synthesized by the liver and its TG content is about 50%–65%, together with CM it is collectively called triglyceride-rich lipoprotein (TRL). VLDL molecule is smaller than CM. When TG is normal, the serum of 12-h fasting is clear and transparent, but when the fasting serum TG level is > 3.4 mmol/L, the serum appears milky and shiny up to cloudy.

### 3.3 Low-density lipoprotein (LDL)

LDL is converted from VLDL. LDL particles contain about 50% of cholesterol and are the lipoproteins with the highest cholesterol content in the blood, so they are called cholesterol-rich lipoproteins. Due to the small size of LDL particles, serum is not cloudy even if the concentration of LDL-C is high.

More than 95% of apolipoprotein in LDL is ApoB100. LDL transports cholesterol to peripheral tissues, and most LDL is catabolized by LDL receptors (LDLR) in hepatocytes and extrahepatic tissues. LDL plays a key role in the development and progression of atherosclerosis. Additionally, differences in physicochemical, metabolism and function lead to a certain heterogeneity between LDL particles. Depending on the particle size and density, LDL can be divided into different subfractions, including large and light, intermediate and small dense LDL (sdLDL). The latter may exert a stronger atherogenic effect (Boren et al., 2020).

### 3.4 High-density lipoprotein (HDL)

HDL is mainly synthesized by the liver and small intestine and is the smallest lipoprotein, being almost half lipid and half protein. The apolipoprotein in HDL is predominantly ApoA1. HDL is also a type of heterogeneous lipoprotein and can be divided into different subfractions. These HDL subfractions differ in shape, density, particle size, charge and anti-atherogenic properties.

### 3.5 Lipoprotein(a) (Lp(a))

Lp(a) consists of LDL-like particles and Apo(a), which are covalently bound by disulfide bonds. Lp(a) has a significant polymorphism derived from the variable length of the Apo(a) peptide chain. Unlike LDL, Lp(a) cannot be converted from VLDL or to other lipoproteins and is an independent class of lipoproteins synthesized by the liver.

Currently, the vast majority of studies support Lp(a) as an independent risk factor for ASCVD and calcified aortic stenosis (Ong et al., 2021; Li et al., 2022; Mehta et al., 2022).

### 3.6 Triglyceride-rich lipoprotein (TRL)

TRL contains both CM and VLDL and is rich in TG. ApoB is the predominant structural protein of TRL. VLDL containing ApoB100 is synthesized by the liver and can be metabolized into VLDL residues, IDL and LDL. CM containing ApoB48 is synthesized by the small intestine, has a larger diameter, and can be metabolized into CM residues.

TRL and its residuals are associated with ASCVD risk. In populations treated with statins, TRL remains an independent factor for residual lipid-related cardiovascular risk in addition to LDL-C, especially in some special populations such as diabetic patients (Chapman et al., 2011; Vallejo-Vaz et al., 2018; Chait et al., 2020; Mach et al., 2020; Raposeiras-Roubin et al., 2021).

## 4 lipid testing programs

Key points.

1. Routine clinical lipid tests include TC, TG, LDL-C and HDL-C; ApoA1, ApoB and Lp(a) have been used as lipid tests by an increasing number of clinical laboratories.
2. Non-HDL-C can be obtained by calculation and is a secondary target of intervention with lipid-lowering therapy.

Routine clinical lipid tests include TC, TG, LDL-C, and HDL-C. It is easy and practical to obtain non-HDL-C by subtracting HDL-C from TC. Many large hospitals in China also conduct ApoA1, ApoB, and Lp(a) tests (Yan, 2003; Laboratory Medicine Society of Chinese Medical Association et al., 2022). In addition, sdLDL-C, lipoprotein particles or subfractions are available in some specialized organizations, and their clinical application value is also gaining attention (Yan, 2008; Liu and Zhao, 2021; Laboratory Medicine Society of Chinese Medical Association et al., 2022).

### 4.1 Total cholesterol (TC)

TC is the sum of the cholesterol contained in each lipoprotein in the blood. The main factors that affect TC levels are.

(1) Age and gender: TC level often increases with age but plateaus or even decreases after the age of 70. TC level is also lower in young and middle-aged women compared to men whereas it is higher in postmenopausal women than in men of the same age.
(2) Dietary habits: long-term high cholesterol, high saturated fatty acid intake can cause TC elevation.
(3) Genetic factors: Mutations in enzymes or receptor genes related to lipoprotein metabolism are the main reason for the significant increase in TC.
Fasting or non-fasting blood specimens can be used for TC tests, with no significant difference in results.

### 4.2 Triglyceride (TG)

In addition to genetic factors, race, age, gender, and lifestyle habits (e.g., diet, exercise, *etc.*) may contribute to higher triglyceride levels. TG levels are highly variable within and between individuals, and the TG level of the same individual is influenced by diet and different time of day, so there may be large differences in TG values when the same individual is measured multiple times (Yan, 2003; Laboratory Medicine Society of Chinese Medical Association et al., 2022). Serum TG levels show a significant skewed distribution in the population. Postprandial TG levels can be elevated (approximately 0.3 mmol/L) regardless of lipid abnormalities; if non-fasting serum TG is ≥ 4.5 mmol/L, a fasting specimen should be collected for lipid testing to assess TG concentrations (Yan, 2008; Liu and Zhao, 2021).

### 4.3 Low-density lipoprotein cholesterol (LDL-C)

Cholesterol accounts for about 50% of LDL particles, and LDL-C concentrations basically reflect blood LDL particle levels. The same factors that affect TC can also affect LDL-C levels. LDL-C can be calculated directly by using Friedewald’s formula (LDL-C = TC − HDL-C − TG/2.2), which was once the most common method of LDL-C determination internationally and is still used in many countries (Yan, 2003; Laboratory Medicine Society of Chinese Medical Association et al., 2022). However, for patients with TG ≥ 4.5 mmol/L or certain abnormal lipoproteinemia, it is appropriate to use the direct assay to detect serum LDL-C level (Jacobson et al., 2015a; He and Yan, 2021). Homogeneous phase method is the most commonly used method for LDL-C determination in China at present (Yan, 2003; He and Yan, 2021; Laboratory Medicine Society of Chinese Medical Association et al., 2022).

### 4.4 High-density lipoprotein cholesterol (HDL-C)

The HDL-C level is clearly influenced by genetic factors. Severe malnutrition is accompanied by significantly lower serum TC and lower HDL-C. Obesity and smoking can also result in lower HDL-C, Research indicates that conditions like diabetes, hepatitis and cirrhosis are related to low HDL-C. Patients with hypertriglyceridemia often have low HDL-C, whereas physical activity (exercise) is associated with mild increases in HDL-C.

The cholesterol content of HDL is relatively stable, so the amount of cholesterol that HDL contains is currently measured to indirectly obtain the level of HDL in the blood. In general, serum HDL-C levels are inversely correlated with the risk of ASCVD (Yan, 2003; Gotto and Brinton, 2004; Laboratory Medicine Society of Chinese Medical Association et al., 2022).

### 4.5 Non-high-density lipoprotein cholesterol (non-HDL-C)

Non-HDL-C is the sum of cholesterol contained in lipoproteins other than HDL in the blood, including cholesterol in VLDL, IDL, LDL and Lp(a). Non-HDL-C represents the total amount of cholesterol in ApoB-containing lipoprotein particles and is calculated as follows: Non-HDL-C = TC − HDL-C. Some international guidelines recommend non-HDL-C as the primary target for primary and secondary prevention of ASCVD (Jacobson et al., 2015a; Boren et al., 2020).

### 4.6 Apolipoprotein A1 (Apo A1)

ApoA1 levels in the normal population are mostly within the range of 1.20–1.60 g/L, and slightly higher in women than in men. ApoA1 is the main protein component of HDL particles (about 65%–75%), while ApoA1 is very little in other lipoproteins. Thus, serum ApoA1 can reflect the level of HDL particles and is significantly positively correlated with HDL-C, and its clinical significance is generally similar (Yan, 2003; Laboratory Medicine Society of Chinese Medical Association et al., 2022). In rare cases, such as patients with familial hypertriglyceridemia, their HDL-C tends to be low, but ApoA1 is not necessarily low, and measuring ApoA1 and HDL-C simultaneously can help clinical diagnosis.

### 4.7 Apolipoprotein B (ApoB)

Serum ApoB is in the range of 0.80–1.10 g/L in the normal population. Under normal conditions, each LDL, IDL, VLDL and Lp(a) particle contains one molecule of ApoB. ApoB has two subclasses, ApoB48 and ApoB100, the former being mainly present in CM and the latter in LDL. Unless otherwise stated, ApoB is usually referred to as ApoB100 in routine clinical measurements.

Serum ApoB mainly reflects the level of LDL particles and is significantly positively correlated with serum LDL-C level, and the clinical significance of both is similar (Yan, 2003; Laboratory Medicine Society of Chinese Medical Association et al., 2022). In some cases, such as hypertriglyceridemia, due to the increase of TRL and residual particles as well as sdLDL particles, the ApoB level is high and the cholesterol level is relatively low, therefore, the so-called “hyper-ApoB” may occur in which the LDL-C level is not high but the serum ApoB level is increased. Thus, simultaneous measurement of ApoB and LDL-C is beneficial for clinical ASCVD risk determination.

### 4.8 Lipoprotein (a) (Lp(a))

Serum Lp(a) concentrations are mainly genetically related, with a significant skewed distribution of Lp(a) levels in the normal population, with geographic and ethnic differences. A cut-off point of 300 mg/L is usually used, above which the risk of CVD increases (Yan, 2003; Laboratory Medicine Society of Chinese Medical Association et al., 2022). An increase in Lp(a) is an independent risk factor for coronary heart disease, ischemic stroke, peripheral vascular disease, coronary artery calcification, and calcified aortic stenosis. In addition, increased Lp(a) is also seen in a variety of inflammatory responses, nephrotic syndrome, diabetic nephropathy, pregnancy, the administration of growth hormones, *etc.* (Wilson et al., 2019; Beijing Society of Cardiology, 2021; Laboratory Medicine Society of Chinese Medical Association et al., 2022).

As Apo(a) has obvious polymorphism, different Apo(a) isomers have different molecular weights, resulting in different Lp(a) test methods to obtain results are not completely consistent, the unit of detection results are nmol/L and mg/L, but the two cannot be directly converted (Wilson et al., 2019; Beijing Society of Cardiology, 2021; Laboratory Medicine Society of Chinese Medical Association et al., 2022).

### 4.9 Small, dense LDL-C with lipoprotein particles and subfractions (sdLDL)

Compared to large particle LDL, the sdLDL subtype is considered to have a more prominent role in promoting the onset and progression of atherosclerosis. Serum sdLDL-C is mostly in the range of 0.2 mmol/L to 1.4 mmol/L in the normal population, and sdLDL-C measurement is useful for ASCVD risk assessment and determination of the severity of relevant diseases (Wu and Wang, 2017; Laboratory Medicine Society of Chinese Medical Association et al., 2022).

In addition, the use of new techniques such as novel vertical automated density gradient ultracentrifugation and magnetic resonance spectroscopy, which can detect various lipoprotein subfraction cholesterol levels and particle concentrations, may be an aid in assessing the residual lipid-related risk of ASCVD (Xu et al., 2015; He and Yan, 2021; Laboratory Medicine Society of Chinese Medical Association et al., 2022; Zhang et al., 2022).

The unit of the measured values of each lipid item is mmol/L (or g/L) according to the Chinese national standard while mg/dL is used in certain countries. Interconversion coefficients are shown as follows:

TC, HDL-C and LDL-C: 1.0 mmol/L = 38.6 mg/dL;

TG: 1.0 mmol/L = 88.5 mg/dL; ApoA1 and ApoB: 0.01 g/L = 1 mg/dL.

The accuracy of lipid test results is affected by a variety of factors, and it is recommended that clinical testing work should be performed in accordance with the requirements of *Clinical Lipid Testing*.

## 5 Overall risk assessment for atherosclerotic cardiovascular disease

Key points.

1. Overall ASCVD risk assessment is the basis for lipid intervention decisions.
2. It is recommended to use *The Flow Chart for Overall Risk Assessment of ASCVD in Chinese Adults* based on a long-term cohort study in Chinese population for risk assessment.
3. Further risk assessment for the rest of life should be performed for those aged <55 years with a moderate 10-year risk of ASCVD.
4. Individuals at moderate risk for ASCVD at 10 years and not at high risk for the rest of their lives should be considered for intervention decisions in combination with risk-enhancing factors.

Numerous observational studies and clinical trials have confirmed LDL-C as a pathogenic risk factor for ASCVD (Cholesterol Treatment Trialists Collaboration, 2015; Navarese et al., 2018). However, an individual’s risk of ASCVD depends not only on the level of LDL-C, but also on the number and level of co-existing disease states and other ASCVD risk factors (Liu et al., 2004; Wu et al., 2006; Yang et al., 2016). Even for individuals with the same LDL-C level, other conditions can lead to significant differences in overall ASCVD risk, and the presence of co-morbidities or risk factors can significantly increase the overall risk of ASCVD. In addition, the risk of recurrent cardiovascular events varies significantly among patients who have already developed ASCVD. Even after controlling risk factors such as lipids, blood pressure and blood glucose according to ultra and very high risk criteria, there may still be a high “residual cardiovascular risk” (Kaasenbrood et al., 2016). RCTs have demonstrated that patients with ASCVD at a higher risk benefit more significantly from intensive LDL-C-lowering therapy (Bohula et al., 2017a; Sabatine et al., 2018). Therefore, further risk assessment should also be performed for patients who already have ASCVD, so that interventions can be continuously improved to reduce the risk of recurrence and improve patient prognosis. Whether for primary prevention to prevent the occurrence of ASCVD or secondary prevention to improve the prognosis of ASCVD, a comprehensive evaluation of the overall risk of ASCVD not only helps to determine the decision of lipid-lowering therapy for patients with dyslipidemia, but also helps clinicians to make individualized and comprehensive treatment decisions according to the patient’s risk level, in order to minimize the overall risk of ASCVD in patients while avoiding the potential harm caused by overtreatment.

Currently, all the national and international guidelines for the prevention and treatment of dyslipidemia include the assessment methods of the overall risk of ASCVD development and the criteria of risk stratification (Joint Committee on Chinese Guideline for the Management of Dyslipidemia in Adults, 2016; Grundy et al., 2019; Chinese Society of Cardiology of Chinese Medical Association et al., 2020; Mach et al., 2020). The *2016 Chinese guideline for the Management of Dyslipidemia in Adults* also emphasize that the adoption of different intensity interventions based on the risk of ASCVD is the core strategy for the prevention and treatment of dyslipidemia, and the overall ASCVD risk assessment is the basis for the treatment decision of dyslipidemia. The “ASCVD overall risk assessment flow chart” based on long-term cohort studies in Chinese population is recommended for risk assessment and stratification (Joint Committee on Chinese Guideline for the Management of Dyslipidemia in Adults, 2016). Based on the recommendations for ASCVD risk assessment in the *2016 Chinese guideline for the Management of Dyslipidemia in Adults*, this revision updated the risk assessment process in the 2016 version of the guidelines by combining the latest research evidence with national and international guidelines and consensus: 1) classifying ASCVD prevention into secondary and primary prevention, depending on prior history of ASCVD; 2) risk stratification of ultra (very) high risk in the secondary prevention population already with ASCVD (Bohula et al., 2017a; Sabatine et al., 2018; Atherosclerosis and Coronary Heart Disease Working Group of Chinese Society of Cardiology and Editorial Board of Chinese Journal of Cardiology, 2020; Li et al., 2021); 3) the addition of chronic kidney disease (CKD) stage 3–5 as one of the three conditions directly classified as high risk in the primary prevention population without ASCVD (Chinese Society of Cardiology of Chinese Medical Association et al., 2020).

**FIGURE 1:**
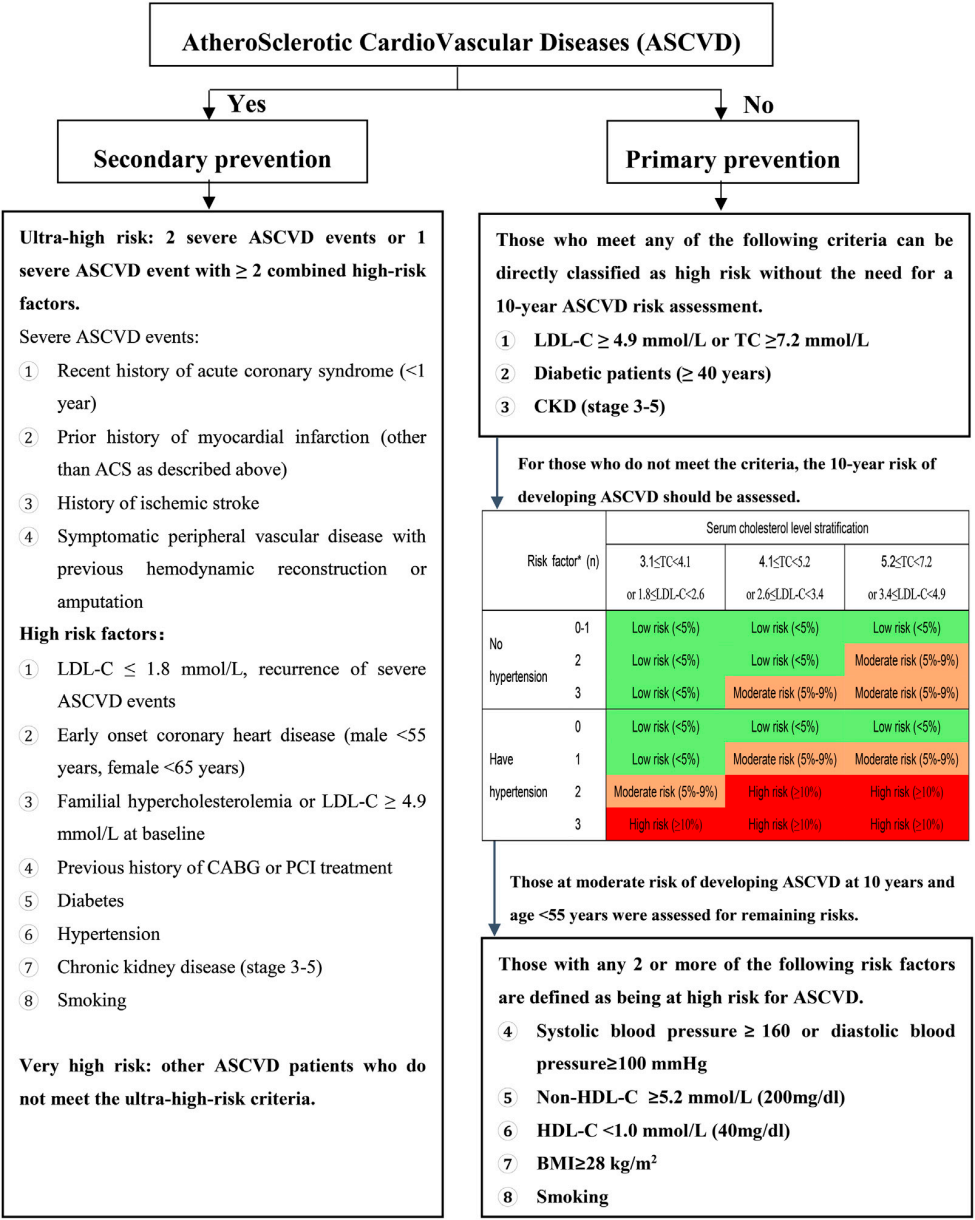
How chart of the overall risk assessment of ASCVD in Chinese adults LDL-C: low-density lipoprotein cholesterol; TC: total cholesterol; CKD: chronic kidney disease; ASCVD: atherosclerotic cardiovascular disease; HDL-C: high-density lipoprotein cholesterol; ACS- acute coronary syndrome; CABG: coronary artery bypass grafting; PCI: percutaneous coronary intervention; BlVIL body mass index. The levels of risk factors are all at pre-intervention levels. *Risk factors included smoking, love HDL-C, and age >45,155 years (male/female); see the section on diabetes in special populations for risk stratification of diabetic patients < −40 years old.

The overall ASCVD risk assessment process is presented in Figure 1.

Firstly, depending on the presence of ASCVD, two categories are classified as secondary prevention and primary prevention.

In patients with ASCVD, those who have had ≥2 severe ASCVD events or 1 severe ASCVD event combined with ≥2 high-risk factors are classified as ultra-high-risk population, and other ASCVD patients are classified as very high-risk.

Among those without ASCVD, those who meet one of the following 3 conditions are directly classified as high-risk and do not require additional 10-year risk assessment for ASCVD: 1) LDL-C ≥ 4.9 mmol/L (or TC ≥ 7.2 mmol/L); 2) diabetic patients aged ≥40 years; or 3) CKD stage 3–5. Individuals without the above-mentioned 3 conditions should be evaluated for overall risk of ASCVD over the next 10 years when considering the need for lipid-lowering therapy. There are 21 “categories”, related to the overall 10-year ASCVD risk assessment, when taking into account the individual’s: serum cholesterol level stratification (TC and LDL-C indices), presence or absence of hypertension and cumulative number of additional risk factors. The 10-year risk is broadly classified as low (<5%), moderate (5%–9%) and high risk (≥10%) and further stratified based on hypertensive status and cholesterol levels. For patients over 55 years of age with a moderate 10-year risk of ASCVD, they should be evaluated for ASCVD risk for the rest of their lives. Those with any 2 or more of the following risk factors are considered at high risk for ASCVD for the rest of their lives: 1) systolic blood pressure ≥160 mmHg (1 mmHg = 0.133 kPa) or diastolic blood pressure ≥100 mmHg; 2) non-HDL-C ≥5.2 mmol/L; 3) HDL-C <1.0 mmol/L; 4) body mass index ≥28 kg/m2; 5) smoking.

It is important to note that in clinical practice, the actual situation of each patient may be complex, especially for those with moderate risk assessment results, and it is sometimes difficult to determine whether to initiate statin therapy. In such cases, the possible co-existence of CVD risk enhancers (Table 1) should also be considered. When patients have multiple risk-enhancing factors combined, they are more inclined to be treated as high-risk. In addition, both doctors and patients can also refer to the digital cardiovascular and cerebrovascular disease risk assessment tool (FuWai Hospital, 2010; Yang et al., 2016) developed and based on population cohort research in our country to fully discuss the risks and to further determine whether to initiate interventions, taking into account the patient’s wishes.

**TABLE 1:** ASCVD risk enhancing factors.

Item	Content
Target organ damage	Coronary artery calcification ≥100 AU (Blaha et al., 2016; Yeboah et al., 2016)
	Ultrasound shows carotid intima-media thickness ≥0.9 mm or presence of carotid atheromatous plaque (Lorenz et al., 2007; Xie et al., 2011; Blaha et al., 2016)
	ABI<0.9 (Blaha et al., 2016; Yeboah et al., 2016)
	Left ventricular hypertrophy: ECG Sv1 + Rv5 (Rv6) voltage >3.8 mV, or echocardiography shows left ventricular mass index >109/105 g/m2 (male/female), or septal thickness ≥11 mm (Echocardiography Group of Chinese Society of Ultrasound in Medicine, 2016; Zhang et al., 2020; Sheng et al., 2022)
Serum biomarkers	Non-HDL-C≥4.9 mmol/L (Sniderman et al., 2011; Emerging Risk Factors Collaboration, 2012; Cao Y et al., 2019)
	ApoB≥1.3 g/L (Sniderman et al., 2011; Emerging Risk Factors Collaboration, 2012)
	Lp(a)≥500 mg/L (Emerging Risk Factors Collaboration, 2012; Willeit et al., 2014)
	TG ≥ 2.3 mmol/L (Nordestgaard and Varbo, 2014; Nordestgaard, 2016; Madsen et al., 2018)
	High-sensitivity C-reactive protein≥2.0 mg/L (Blaha et al., 2016; Yeboah et al., 2016)
Other factors	Obesity or abdominal obesity, family history of early onset cardiovascular disease [age of onset <55/65 years (male/female)] (Blaha et al., 2016; Yang et al., 2016)*etc.*

ABI: ankle/arm blood pressure index; non-HDL-C: non-HDL, cholesterol; ApoB: apolipoprotein B; Lp(a): lipoprotein(a); TG: triglycerides.

## 6 Reference standards for appropriate blood lipid levels

Key points.

1. The reference level for LDL-C is only for those at low overall risk for ASCVD.
2. The overall risk of ASCVD should be evaluated when determining the level of LDL-C control in patients in clinical practice.

Among the commonly used lipid markers, the one that is causally related to the risk of ASCVD development, and which is the primary clinical therapeutic target is LDL-C. For different populations at risk for ASCVD, the target level of LDL-C and the criteria for determining elevated LDL-C vary, as do the LDL-C level and the therapeutic target of LDL-C for initiating lipid-lowering drug therapy (Boren et al., 2020; Mach et al., 2020). Since most Chinese adults aged ≥18 years are at low risk for ASCVD (Zhang et al., 2018), Table 2 lists the reference standards of major lipid indicators applicable to the low-risk population for ASCVD, which can help medical professionals and the public to have basic knowledge of lipid levels. Due to the increasing use of non-HDL-C and Lp(a) data in clinical practice, reference values for their target levels are also listed in the table (Table 2).

**TABLE 2:** Reference standards for major lipid indicators in low-risk groups for primary prevention of ASCVD in China^a^.

Classification	TC	LDL-C	HDL-C	TG	Non-HDL-C	Lp(a)
Ideal level	-	<2.6	-	-	<3.4	-
Reasonable level	<5.2	<3.4	-	<1.7	<4.1	<300
Slight elevation	≥5.2&<6.2	≥3.4&<4.1	-	≥1.7&<2.3	≥4.1&<4.9	-
Elevation	≥6.2	≥4.1	-	≥2.3	≥4.9	≥300
Reduction	-	-	<1.0	-	-	-

TC: total cholesterol; LDL-C: low-density lipoprotein cholesterol; HDL-C: high-density lipoprotein cholesterol; TG: triglycerides; Lp(a): lipoprotein(a).^a^: Reference standard for ASCVD, primary prevention low-risk population only. Values listed in the table are lipid levels measured at 12-h fasting before the intervention. -: None. Lp(a) units are mg/L, the rest are mmol/L.

## 7 Classification of dyslipidemia

Key points.

The classification of dyslipidemia is complicated, and there are 2 commonly used ones, the etiological classification, and the clinical classification. The most practical one is the clinical classification.

Dyslipidemia usually refers to elevated serum cholesterol and/or TG levels, commonly known as hyperlipidemia. Dyslipidemia may also refer to a wide range of lipid irregularities, such as low HDL-C. There are 2 common classifications: etiological classification and clinical classification. The most practical is the clinical classification (Zhao, 2003; Expert Dyslipidemia Panel, 2013; Jacobson et al., 2015a).

### 7.1 Etiological classification of dyslipidemia

#### 7.1.1 Primary (hereditary) dyslipidemia

Primary dyslipidemia is diagnosed only after secondary dyslipidemia has been excluded. Most of the primary dyslipidemia is due to single or multiple gene mutations, and it has family aggregation and obvious hereditary tendency, especially for single gene mutations, so it is also clinically known as hereditary or familial hyperlipidemia.

Familial hypercholesterolemia (FH) is a monogenic, autosomal inherited abnormality of cholesterol metabolism that is most often dominantly inherited. It is rarely recessively inherited. Currently, there are three dominant genes recognized as causative for FH: LDL receptor (LDLR), ApoB, and PCSK9; and one recessive gene: LDL receptor adaptor protein 1 (LDLRAP1). LDLR pathogenic mutations are responsible for ≥90% of FH patients, followed by ApoB pathogenic mutations, with the latter having a higher proportion in Chinese FH patients (Sun et al., 2018). With the development of gene sequencing technology, more and more genes, such as signal transducing adaptor protein 1 (STAP1), lysosomal acid lipase (LIPA), ATP binding cassette subfamily 6 member 5 (ABCG and ApoE, have been suggested to be possibly associated with the pathogenesis of FH as well) (Cao et al., 2021).

FH genotypes can be divided into heterozygous FH (HeFH) (compound heterozygous FH and double heterozygous FH) and homozygous FH (HoFH). HeFH is the most common FH genotype. The prevalence of HeFH is estimated to be 1/250 to 1/200 and while that of HoFH is estimated to be 1/(160,000 to 320,000). The risk of ASCVD is significantly higher in FH patients because they are exposed to high serum LDL-C levels from birth (Nordestgaard et al., 2013; Sharifi et al., 2016).

Familial hypertriglyceridemia is the result of a single gene mutation, usually in the lipoprotein lipase (LPL) or ApoC2 or ApoA5 genes involved in TG metabolism (Willer et al., 2013), and manifests as severe hypertriglyceridemia (TG > 10 mmol/L) with an incidence of about 1 in 1 million. Mild to moderate hypertriglyceridemia is usually characterized by multiple gene mutations (Hegele et al., 2014) (Table 3).

**TABLE 3:** Primary (hereditary) lipoprotein metabolism-related diseases.

Disease name	Estimated prevalence	Pathogenic genes	Effect on lipoproteins
HeFH	1/250–1/200	LDLR, ApoB, PCSK9	LDL-C ↑
HoFH	1/320,000–1/160,000	LDLR, ApoB, PCSK9, LDLRAP1	LDL-C↑↑
Mixed familial hyperlipidemia	1/200–1/100	Upstream transcription factor 1+ modifier gene	LDL-C↑, VLDL-C↑, ApoB↑
Familial dysβ-lipoproteinemia	1/5000	ApoE	IDL and VLDL residual particles (βVLDL) ↑↑
Familial lipoprotein lipase deficiency (familial chylomicronemia syndrome)	2/1 000 000	LPL, APOC2, ApoA5, GPIHBP1, LMF1	Chylomicron and VLDL-C↑↑
Tangier Disease (absence of α lipoproteinemia)	1/1 000 000	ABCA1	HDL-C↓↓
Familial LCAT	1/1 000 000	LCAT	HDL-C↓

HeFH: heterozygous familial hypercholesterolemia; HoFH: homozygous familial hypercholesterole-mia; LCAT: lecithin cholesterol acyltransferase.

#### 7.1.2 Secondary (acquired) dyslipidemia

Secondary dyslipidemia is usually defined as dyslipidemia caused by underlying systemic diseases that lead to alterations in serum lipid and lipoprotein metabolism; altered metabolic status; unhealthy diet, and certain medications. Secondary dyslipidemia may have similar consequences as primary dyslipidemia.

Diets such as those rich in saturated fatty acids and cholesterol can cause elevated cholesterol levels, whereas excessive alcohol can lead to hypertriglyceridemia. Medications, such as glucocorticoids, estrogens, retinoids, cyclosporine, antidepressants, vascular endothelial growth factor inhibitors, aromatase inhibitors, *etc.*, Can also cause secondary dyslipidemia.

The most common diseases that cause dyslipidemia are obesity, diabetes, nephrotic syndrome, hypothyroidism, renal failure, liver disease, systemic lupus erythematosus, glycogen accumulation, myeloma, lipodystrophy, acute porphyria, polycystic ovary syndrome, *etc.*

### 7.2 Clinical classification of dyslipidemia

The clinical classification of dyslipidemia is summarized in Table 4.

**TABLE 4:** Clinical classification of dyslipidemia.

Classification	TC	TG	HDL-C	Equivalent to WHO phenotype (Beaumont et al., 1970)
Hypercholesterolemia	Increase	**-**	**-**	Ⅱa
Hypertriglyceridemia	**-**	Increase	**-**	Ⅳ, Ⅰ
Mixed hyperlipidemia	Increase	Increase	**-**	Ⅱb, Ⅲ, Ⅳ, Ⅴ
Low HDL-C	**-**	**-**	Decrease	**-**

TC: cholesterol; TG: triglycerides; HDL-C: high-density lipoprotein cholesterol; WHO: world health organization; -: none.

## 8 Blood lipid screening

Key points.

1. Lipid testing is fundamental for detecting dyslipidemia, assessing ASCVD risk, and identifying intervention strategies.
2. Lipid screening is an effective way to improve the early detection and awareness of dyslipidemia.
3. The frequency of lipid testing should be based on age, ASCVD risk, and the need for monitoring of therapeutic interventions.

Early detection of dyslipidemia and monitoring of changes in lipid levels is an important basis for assessing ASCVD risk and implementing effective ASCVD prevention and treatment measures. Routine medical services and health checkups are crucial for early detection of dyslipidemia. Although most medical institutions in China are equipped with routine lipid testing, the detection rate and awareness rate of dyslipidemia in adults remain low. It is recommended to increase the detection and awareness of dyslipidemia by: 1) raising public awareness of the importance of regular lipid testing; 2) increasing the availability of lipid testing for patients in routine medical services; 3) encouraging health screening services to include lipid testing as a routine item; and 4) including lipid testing for children and adolescents as a routine item in primary, middle and high school entry physical examinations.

The frequency of lipid screening and testing indices are recommended below.

1. Lipid testing (including TC, LDL-C, HDL-C and TG) should be performed every 2–5 years in adults <40 years of age and at least once a year in adults ≥40 years of age (Healthy China Initiative Promotion Committee, 2019);
2. People at high risk for ASCVD (see the section on overall cardiovascular risk assessment) should have lipid testing based on the need for individualized control;
3. At least 1 Lp(a) test should be included in the lipid testing of the high risk population (Mach et al., 2020; Beijing Society of Cardiology, 2021);
4. Lipid testing should be included as a routine item in primary, middle and high school physical examinations;
5. First- and second-degree relatives of people with preexisting FH should be screened for lipids to increase the early detection rate of FH.

Lipid screening is paramount in: 1) People with a history of ASCVD. 2) People with multiple ASCVD risk factors (e.g., hypertension, diabetes, obesity, smoking). 3) People with a family history of early-onset ASCVD (defined as ischemic cardiovascular disease before age 55 for first-degree immediate family members in men, or 65 years of age for first-degree immediate family members in women), or people with familial hyperlipidemia. 4) Patients with xanthomas of the skin or in the tendons over the knuckles or in the Achilles tendons.

## 9 Principles of dyslipidemia treatment

Key points.

1. LDL-C is the primary target of intervention against ASCVD, and non-HDL-C is the secondary target of intervention.
2. The corresponding LDL-C and non-HDL-C target values are determined according to the individual’s ASCVD risk.
3. A heart-healthy lifestyle is fundamental for lowering LDL-C and non-HDL-C.
4. LDL-C-lowering therapy is initiated with moderate doses of statins.
5. Combination of cholesterol absorption inhibitors and/or PCSK9 inhibitors should be considered when LDL-C targets are not met after statin therapy.
6. High-risk ASCVD patients with elevated TG despite statin therapy may be combined with high-purity eicosapentaenoic or high-purity omega-3 fatty acids or fibrates to reduce ASCVD risk.

### 9.1 Lipid intervention targets and management

Clinical evidence from studies related to lipids, epidemiology, genetics, and clinical interventions have been evaluated and summarized in order to propose primary and secondary targets of intervention and management recommendations for lipid management (Table 5).

**TABLE 5:** Lipid intervention targets and management recommendations.

Recommendation	Recommended classification	Evidence level
LDL-C as a primary target for ASCVD risk intervention (Cholesterol Treatment Trialists Collaboration, 2015; Silverman et al., 2016; Navarese et al., 2018)	Ⅰ	A
Non-HDL-C as a target for ASCVD risk intervention in patients with diabetes, metabolic syndrome, high TG, and VLDL-C (Boekholdt et al., 2012; Thanassoulis et al., 2014)	Ⅰ	B
ApoB as a secondary target for ASCVD risk intervention in patients with diabetes, metabolic syndrome, high TG, and VLDL-C (Boekholdt et al., 2012; Thanassoulis et al., 2014)	Ⅱa	C
High TG as an indicator for management of patients at high risk of ASCVD after LDL-C reaching the target (Keech et al., 2005; Jun et al., 2010; Bhatt et al., 2019)	Ⅱa	B
High Lp(a) as an indicator for the management of patients at high risk of ASCVD (Emerging Risk Factors Collaboration, 2009; Willeit et al., 2014)	Ⅱa	C
HDL-C is not recommended as a target for intervention	Ⅲ	A

LDL-C: low-density lipoprotein cholesterol; ASCVD: atherosclerotic cardiovascular disease; HDL-C: high-density lipoprotein cholesterol; ApoB: apolipoprotein B; TG: triglycerides; Lp(a): lipoprotein(a).

#### 9.1.1 LDL-C: Primary lipid-lowering target

Conventional lipid markers to assess ASCVD risk include TC, LDL-C, HDL-C, and TG. LDL-C has been used in the majority of lipid-lowering intervention studies to observe the relationship between the effect of lipid lowering and the reduction in ASCVD risk. Meta-analyses have indicated that for every 1 mmol/L reduction in LDL-C, there is a 20%–23% reduction in ASCVD events (Cholesterol Treatment Trialists Collaboration, 2015; Silverman et al., 2016; Navarese et al., 2018). Therefore, the majority of national or regional/international guidelines for lipid management recommend LDL-C as the primary goal of lipid-lowering therapy.

#### 9.1.2 Non-HDL-C: Secondary lipid-lowering target

All ApoB-containing lipoprotein particles have potentially atherogenic effects. In the context of increased TRL ratios, such as hypertriglyceridemia, diabetes, metabolic syndrome, obesity, and VLDL-C, *etc.*, LDL-C has limitations as a primary target, whereas non-HDL-C represents the cholesterol in the full range of atherogenic lipoprotein particles. Some studies have confirmed that non-HDL-C is a better predictor of ASCVD risk than LDL-C, regardless of whether or not one is treated with statins (Boekholdt et al., 2012; Thanassoulis et al., 2014). Although the lipid-lowering target of interest in statin studies was LDL-C, and statins can mildly lower TG and elevate HDL-C, in a meta-analysis of statin studies, ASCVD reduction was found to correlate better with non-HDL-C reduction than with LDL-C reduction. In addition, non-HDL-C is easier to calculate, and the results are stable and less affected by TG fluctuations and after meals. Non-HDL-C is suitable as a lipid-lowering target for special populations with mild to moderate TG elevation, diabetes, metabolic syndrome, obesity, and VLDL-C.

#### 9.1.3 Other intervention indicators

(1) ApoB: All atherogenic lipoprotein particles, regardless of particle size, contain one molecule of ApoB. Therefore, ApoB test is theoretically a more accurate indicator of the number of atherogenic lipoprotein particles. Some studies also suggest that ApoB is a better predictor of ASCVD risk than LDL-C or non-HDL-C (Boekholdt et al., 2012; Thanassoulis et al., 2014). However, ApoB measurement is not yet widespread, the cost of the test is relatively high, and evidence from relevant clinical intervention studies is lacking, so it is not recommended as a primary target for lipid-lowering therapy at this time.
(2) TG: It is an independent risk factor for ASCVD and is also identified as a risk enhancer for ASCVD risk in the risk stratification. Patients with high TG even after reaching LDL-C target should be treated with concomitant TG-lowering therapy in order to further reduce ASCVD risk. In addition, in patients with severe high TG, lowering TG can reduce the risk of pancreatitis.
(3) Lp(a): Numerous epidemiological and genetic studies have shown that Lp(a) is strongly associated with ASCVD and aortic valve calcification (Emerging Risk Factors Collaboration, 2009; Willeit et al., 2014). Large clinical studies to reduce cardiovascular outcomes of Lp(a) are underway.
(4) HDL-C: Low HDL-C is an independent risk factor for ASCVD, but increasing HDL-C through medications has not reduced the risk of ASCVD, so HDL-C is not currently considered a target for lipid intervention.

### 9.2 Target values for lipid intervention targets

Based on the results of large-scale clinical studies, target values for LDL-C and non-HDL-C are proposed for individuals in different risk classes to effectively reduce ASCVD risk (Table 6).

**TABLE 6:** Target values of lipid-lowering targets.

Risk level	LDL-C recommended target values (mmol/L)	Recommended classification	Evidence level
Low risk	<3.4 (Cholesterol Treatment Trialists Collaborators, 2012)	Ⅱa	B
Medium and high risk^a^	<2.6 (Ridker et al., 2008; Cholesterol Treatment Trialists Collaboration, 2010; Cholesterol Treatment Trialists Collaboration, 2015; Yusuf et al., 2016a)	I	A
Very high risk	<1.8 and >50% reduction from baseline (Cholesterol Treatment Trialists Collaboration, 2010; Cannon et al., 2015; Bangalore et al., 2016; Ridker et al., 2016; Sabatine et al., 2017; Schwartz et al., 2018)	I	A
Ultra-high risk	<1.4 and >50% reduction from baseline (Cholesterol Treatment Trialists Collaboration, 2010; Cannon et al., 2015; Bangalore et al., 2016; Ridker et al., 2016; Sabatine et al., 2017; Schwartz et al., 2018)	I	A

LDL-C: low-density lipoprotein cholesterol.^a^ See the section on diabetes in special populations for lipid targets in ASCVD, high-risk patients with comorbid diabetes. Non-HDL-C, target level = LDL-C + 0.8 mmol/L.

The principle for setting target values for lipids in ASCVD prevention and treatment was not only derived from the results of large-scale RCTs and meta-analyses, but also from Mendelian randomization studies and observational studies. Although these studies did not systematically explore specific target values for LDL-C, the results of the meta-analyses of these studies consistently showed that the greater the LDL-C reduction and the longer the duration, the greater the reduction in ASCVD risk.

Several clinical studies of primary prevention with statins have shown that LDL-C reduction to below 2.6 mmol/L with moderate-intensity statins significantly reduces the risk of ASCVD or all-cause mortality compared with placebo in both moderate-risk and high-risk patients (Ridker et al., 2008; Cholesterol Treatment Trialists Collaboration, 2010; Cholesterol Treatment Trialists Collaboration, 2015). Results from secondary prevention clinical studies in very high-risk patients suggest that LDL-C reduction to less than 1.8 mmol/L further significantly reduces the risk of ASCVD (Cholesterol Treatment Trialists Collaboration, 2010). A meta-analysis of secondary prevention studies showed that for patients who reached an LDL-C of 1.8 mmol/L or less after high-dose statin therapy, an LDL-C reduction of ≥50% further reduced ASCVD risk compared with <50%, suggesting that an LDL-C reduction of ≥50% could be a target for intensive lipid lowering (Bangalore et al., 2016; Ridker et al., 2016). Studies of statins combined with ezetimibe or PCSK9 monoclonal antibodies have shown that LDL-C reduction below 1.4 mmol/L still further reduces ASCVD risk, and the higher the baseline risk, the greater the absolute ASCVD risk reduction (Cannon et al., 2015; Sabatine et al., 2017; Schwartz et al., 2018). Post hoc analysis of a randomized controlled trial showed that ASCVD event reduction was linearly and negatively correlated with LDL-C levels even when LDL-C was below 1 mmol/L (Sabatine et al., 2017).

However, the cost-effectiveness of lipid reduction should be considered when determining LDL-C treatment goals. To ensure appropriate cost-effectiveness, following factors need to be considered: the absolute LDL-C reduction value after treatment and the baseline risk of treated subjects. Therefore, it is appropriate to set different LDL-C targets according to the different baseline ASCVD risks of patients, which means that the higher the baseline risk, the lower the LDL-C target value should be.

### 9.3 Strategies for lowering lipids to meet targets

Strategies for lipid-lowering treatment include lifestyle interventions and pharmacotherapy (Table 7).

**TABLE 7:** Recommended strategies for achieving lipid reduction.

Recommendation	Recommended classification	Evidence level
1. Lifestyle interventions are the foundation of lipid-lowering treatment	I	B
2. Moderate-intensity statins as initiation therapy for achieving lipid-lowering target (Scandinavian Simvastatin Survival Study Group, 1994; Shepherd et al., 1995; Sacks et al., 1996; Downs et al., 1998; Collins et al., 2003; Sever et al., 2003; Colhoun et al., 2004; Nakamura et al., 2006; Zhao et al., 2014; Yusuf et al., 2016a; Diaz et al., 2021)	I	A
3. Combination of cholesterol absorption inhibitors for those who cannot reach LDL-C target with moderate-intensity statin therapy (Cannon et al., 2015; Kim et al., 2022)	I	A
4. Moderate-intensity statins combined with cholesterol absorption inhibitors LDL-C still cannot achieve the target, combined with PCSK9 inhibitors (Sabatine et al., 2017; Schwartz et al., 2018)	I	A
5. Ultra-high-risk patients with high baseline LDL-C levels^a^ who are expected to have difficulty achieving the target with a statin combined with a cholesterol uptake inhibitor may be initiated directly on statin combined with PCSK9 inhibitor therapy (Sabatine et al., 2017; Schwartz et al., 2018)	IIa	A
6. Patients who cannot tolerate statins should consider cholesterol absorption inhibitors or PCSK9 inhibitors (Moriarty et al., 2015; Nissen et al., 2016; Schreml and Gouni-Berthold, 2018)	IIa	C

LDL-C: low-density lipoprotein cholesterol; PCSK9: proprotein convertase chymotrypsin 9. ^a^LDL-C ≥ 2.6 mmol/L in those taking statins and LDL-C ≥ 4.9 mmol/L in those not taking statins.

The first recommendation in lipid-lowering therapy is a heart-healthy lifestyle; including a proper diet, moderate increase in physical activity, weight management, smoking cessation and alcohol restriction. Of these, a proper diet has a greater impact on blood lipids (Table 8). Regarding the dietary recommendations in ASCVD prevention, there is a more consistent understanding to limit the intake of saturated fatty acids and trans fats, and increase the intake of fruits and vegetables, whole grain potatoes, dietary fiber and fish (Table 9).

**TABLE 8:** Effect of lifestyle on blood lipids.

Lifestyle effects on blood lipids	Recommended classification	Evidence level
Lower TC and LDL-C		
Weight management	I	A
Increase physical activity	IIa	B
Lower TG		
Reduce alcohol consumption	I	A
Increase physical activity	I	A
Weight management	I	A
Elevate HDL-C		
Increase physical activity	I	A
Weight management	I	A
Smoking cessation	IIa	B

TC: total cholesterol; LDL-C: low-density lipoprotein cholesterol; TG: triglycerides; HDL-C: high-density lipoprotein cholesterol.

**TABLE 9:** Recommendations for lipid-lowering dietary therapy.

Recommendation	Recommended classification	Evidence level
1. Total fat intake should be limited to 20–25 g per day. Unsaturated fatty acids (vegetable oils) are used instead of saturated fatty acids (animal oils, palm oil, *etc.*) (Mozaffarian et al., 2009; Mozaffarian et al., 2010; Clifton and Keogh, 2017)	IIa	A
2. Avoid trans fats (hydrogenated vegetable oils, *etc.*) (Mozaffarian et al., 2009; Mozaffarian et al., 2010)	Ⅲ	A
3. Lowering dietary cholesterol intake should be considered in people at moderate risk of ASCVD or above or in patients with combined hypercholesterolemia (Ginsberg et al., 1994; Ginsberg et al., 1995; Tanasescu et al., 2004; Djousse et al., 2009; Wang et al., 2022)	IIa	B

ASCVD: atherosclerotic cardiovascular disease.

The healthy dietary patterns recommended by international guidelines are mainly to control hypertension as in “DASH” diets (United States) and “Mediterranean diets” (Europe). As the dietary habits of Chinese residents are unique, Chinese scholars have proposed a Chinese heart-healthy dietary pattern. The results of a randomized double-blind parallel-controlled dietary trial has shown that the Chinese heart-healthy diet significantly reduces blood pressure compared with the traditional diet (Wang et al., 2022). This provides a reference for the development of future dietary recommendations for lipid management in the Chinese population. Although studies have shown that dietary cholesterol intake significantly affects serum cholesterol levels (Wang et al., 2022), consistent conclusions regarding the relationship between dietary cholesterol and cardiovascular events have not been achieved due to multiple known and unknown confounding factors. However, in terms of serum cholesterol being a risk factor for ASCVD, elevated serum cholesterol levels from any cause can increase the risk of ASCVD. Therefore, based on the recommended Chinese heart-healthy dietary pattern, special emphasis should be placed on reducing dietary cholesterol intake to less than 300 mg per day for those at high risk of ASCVD and patients with hypercholesterolemia (Ginsberg et al., 1994; Ginsberg et al., 1995; Tanasescu et al., 2004; Djousse et al., 2009; Zhong et al., 2019).

When lifestyle interventions fail to achieve lipid-lowering goals, the addition of lipid-lowering drugs should be considered. Statins are the basis of cholesterol-lowering therapy, but their dose doubling increases the LDL-C lowering effect by only 6% and has potential side effects such as liver function impairment, myopathy, and new-onset diabetes. The China Intensive Lipid Lowering with statins in Acute coronary syndrome Study (CHILLAS) suggests that increasing the dose of statins by 1–2 times did not further reduce cardiovascular events (Zhao et al., 2014). Combined with the fact that the tolerance of high-dose statins in China is poorer than that in Europe and the United States, the use of high-intensity high-dose statins treatment is not recommended, and as such it is recommended to start with conventional doses or moderate-intensity statins.

For statin intolerant individuals, the natural lipid-lowering agent Xuezhikang (XZK) (Chinese Traditional Medicine) can be used as initial lipid-lowering therapy. XZK has good safety and has shown clinical benefit in secondary prevention studies in the Chinese population (Collaborative Group on Xuezhikang for Secondary Prevention of Coronary Artery Disease, 2005; Lu et al., 2008; Li et al., 2010; Venero et al., 2010). When LDL-C targets are not achieved with statins or XZK, non-statin lipid-lowering agents such as cholesterol absorption inhibitors (Kim et al., 2022) or PCSK9 inhibitors (Sabatine et al., 2017; Schwartz et al., 2018) can be added as a combination. Recent studies in Asian populations have shown that in patients with ASCVD, moderate-intensity statins combined with ezetimibe resulted in more participants achieving LDL-C target and had better tolerability than high-intensity statins, with a trend towards lower ASCVD events (Kim et al., 2022). This suggests that moderate-intensity statins combined with non-statin drugs can replace high-intensity statins with better efficacy and safety. In ultra-high-risk patients, when baseline LDL-C is high (LDL-C ≥ 4.9 mmol/L in patients not on statins or LDL-C ≥ 2.6 mmol/L in patients on statins) and LDL-C target is not expected to be achieved with statins combined with cholesterol absorption inhibitors, direct use of statins combined with PCSK9 inhibitors may be considered to ensure early and rapid LDL-C target achievement in patients. Early use of PCSK9 monoclonal antibodies has been shown to reduce ASCVD risk earlier and more significantly, with good safety for prolonged use (≥7 years) (O'Donoghue et al., 2022).

### 9.4 Interventions for other lipid parameters

Numerous epidemiological studies suggest that elevated TG is associated with an increased risk of ASCVD. In addition, Mendelian RCTs also support a causal association of TG with coronary heart disease. A recent Mendelian randomization study (Ference et al., 2019) has found that when both LPL, which promotes TG hydrolysis, and LDLR, which is involved in LDL metabolism, have genetic variants that result in the same magnitude of ApoB changes, they have the same effect on ASCVD risk. This result suggests that the causal association of TRL and its residual particles with ASCVD is determined by the amount of ApoB lipoprotein particles.

Elevated TG is closely related to poor lifestyle and diet. Exercise and diet control can reduce obesity and insulin resistance, thus effectively lowering TG. Alcohol consumption is a very important factor in TG elevation, and individuals with elevated TG need to strictly limit alcohol intake. In addition to limiting the intake of fatty acids in the diet, special attention should be paid to reducing the intake of refined carbohydrates and increasing the intake of fiber-rich low-sugar diet such as whole grain or coarse grains.

Current TG-lowering drugs include niacin analogs, fibrates and high-purity omega-3 polyunsaturated fatty acids (omega-3 fatty acids). All three classes of drugs can be used in patients with severe hypertriglyceridemia to reduce the occurrence of pancreatitis (Table 10). However, the results of clinical trials of the three classes of drugs for ASCVD prevention have been inconsistent. The clinical studies of nicotinic acid analogs were all negative, and they are no longer recommended as TG-lowering agents for the prevention of ASCVD. The primary endpoint outcome of the fibrate intervention studies was neutral, but the results of stratified analyses of single studies or meta-analyses suggested that for those with baseline TG > 2.3 mmol/L, the reduction in ASCVD risk in the fibrate treatment group approached statistical significance (*p* = 0.057). Pemafibrate, a highly selective peroxidase proliferator activated receptor α (PPARα) agonist, showed potent TG reduction. The clinical study Pemafibrate to Reduce Cardiovascular Outcomes by Reducing Triglycerides in Patients with Diabetes (PROMINENT) conducted in and mild to moderate TG elevation at baseline (200–499 mg/dL) and HDL-C ≤40 mg/dL patients with diabetes randomized to placebo or pemafibrate included patients achieving LDL-C target after statin therapy who did not show a difference in clinical events between the two groups, which raises the question of whether TG reduction by fibrates reduces ASCVD risk (Das Pradhan et al., 2022).

**TABLE 10:** Management of high TG.

Recommendation	Recommended classification	Evidence level
When TG > 5.6 mmol/L, treatment with fibrates, high-purity omega-3 fatty acids, or niacin may be used to reduce the risk of pancreatitis (Christian et al., 2012)	I	C
High-dose IPE (2 g twice daily) (Yokoyama et al., 2007; Bhatt et al., 2019) should be considered to reduce ASCVD risk in ASCVD patients and those at high risk of ASCVD who receive moderate doses of statins with TG > 2.3 mmol/L	IIa	B
ASCVD patients and those at high risk of ASCVD who receive moderate doses of statins with TG > 2.3 mmol/L may be given high-purity omega-3 fatty acids (Bhatt et al., 2019; O'Donoghue et al., 2022) or fenofibrate or benzofibrate to further reduce ASCVD risk (Keech et al., 2005; Accord Study Group et al., 2010)	IIb	C

TG: triglycerides; ASCVD: atherosclerotic cardiovascular disease; IPE: icosapent ethyl.

Omega-3 fatty acids refer to fish oil preparations containing primarily eicosapentaenoic acid (EPA) and/or docosahexaenoic acid (DHA). Icosapent Ethyl (IPE) is ethylated EPA. The results of clinical endpoint studies of high-purity omega-3 fatty acids for TG reduction are highly variable. Reduction of Cardiovascular Events with Icosapent Ethyl-Intervention Trial (REDUCE-IT) and the Japan Eicosapentaenoic Acid Lipid Intervention Study (JELIS) showed that combining high-purity IPE or EPA with a statin significantly reduced ASCVD risk (Yokoyama et al., 2007; Bhatt et al., 2019), while studies of statins combined with high-purity omega-3 fatty acids (EPA + DHA) showed a trend toward ASCVD reduction only in the meta-analysis (Hu et al., 2019).

### 9.5 Monitoring of treatment process

The purpose of monitoring in lipid-lowering therapy are: 1) to observe whether the lipid-lowering target value is achieved; 2) to understand the potential adverse effects of drugs. For those who take non-pharmacological treatment such as diet control, the lipid level should be reviewed in the first 3–6 months. If the lipid control reaches the recommended target value, the non-pharmacological treatment should be continued, but should be reviewed every 6 months to 1 year, whilst the long-term standard can be reviewed once a year. If individuals are taking lipid-lowering drugs for the first time, they should review their lipids, liver enzymes and creatine kinase (CK) within 4–6 weeks of taking the drug. If the lipid parameters can reach the target value and there are no adverse drug reactions, gradually change to re-examination every 3–6 months. If the target value of lipid is not reached after 1–3 months of treatment, the dose or type of lipid-lowering drugs or the combination of lipid-lowering drugs with different mechanisms should be adjusted promptly. Whenever the type or dose of lipid-lowering drug is adjusted, it should be reviewed within 4–6 weeks of treatment. Therapeutic lifestyle changes and lipid-lowering drug therapy must be maintained over time to provide better clinical benefit.

## 10 lipid-lowering drug therapy

Key Points.

1. Statins are the cornerstone of lipid-lowering drug therapy for dyslipidemia.
2. Moderate-intensity statins are the preferred strategy for lipid-lowering therapy in the Chinese population.
3. Combination of lipid-lowering drugs is the basic trend of dyslipidemia treatment strategy.
4. Lipid-lowering therapy should be followed up regularly to observe the efficacy and adverse effects and to adjust the treatment plan, and the concept of long-term target achievement should be carefully implemented.

There are many types of lipid-lowering drugs clinically available, and lipid-lowering drugs usually reduce both cholesterol and other lipid components. However, according to their main effects, they are divided into drugs that mainly lower cholesterol and drugs that mainly lower TG. The decision to initiate a combination of lipid-lowering drugs in clinical practice is usually based on the type of dyslipidemia, the baseline level, and the target value to be achieved.

### 10.1 Major cholesterol-lowering drugs

The main mechanism of action of these drugs is to inhibit cholesterol synthesis in hepatocytes and/or increase LDLR in hepatocytes, or to reduce cholesterol absorption in the intestine, or to accelerate LDL catabolism, including statins, cholesterol absorption inhibitors, PCSK9 inhibitors, probucol, bile acid chelators, and other lipid-lowering drugs (lipobitol, polyhexadecanoate).

#### 10.1.1 Statins

Statins, also known as 3hydroxy-3-methylglutaryl coenzyme A reductase inhibitors, inhibit the cholesterol synthesis rate-limiting enzyme, 3hydroxy-3-methylglutaryl coenzyme A reductase, to reduce cholesterol synthesis, while upregulating cell surface LDLR and accelerating serum LDL catabolism. Therefore, statins can significantly reduce serum TC, LDL-C and ApoB levels, and mildly reduce serum TG levels and increase HDL-C levels.

The introduction of statins is a milestone in the history of ASCVD prevention and treatment in humans. Substantial evidence-based evidence confirms that statins significantly reduce cardiovascular events in patients with ASCVD (Scandinavian Simvastatin Survival Study Group, 1994; Willerson, 1996; Long-Term Intervention with Pravastatin in Ischaemic Disease Study Group, 1998; Schwartz et al., 2001; Heart Protection Study Collaborative Group, 2002; Serruys et al., 2002; Cannon et al., 2004; de Lemos et al., 2004; LaRosa et al., 2005; Pedersen et al., 2005; Nissen et al., 2006; Cholesterol Treatment Trialists Collaboration, 2010), and also have a role in primary prevention in people at high risk for ASCVD (Shepherd et al., 1995; Downs et al., 1998; Colhoun et al., 2004). A recent meta-analysis found that statin therapy reduced all-cause mortality by 9%, myocardial infarction by 29%, and stroke by 14% (Byrne et al., 2022). In addition, a recent study found that the application of a moderate dose statin (rosuvastatin 10 mg/d) in combination with ezetimibe for the ASCVD population was not inferior to the high-dose statin group (rosuvastatin 20 mg/d) in reducing cardiovascular events, and the incidence of adverse events was lower than in the high-dose statin group (Kim et al., 2022), suggesting the advantages of combination therapy.

Statins are indicated for the prevention and treatment of hypercholesterolemia, mixed hyperlipidemia and ASCVD. Currently, lovastatin, simvastatin, pravastatin, fluvastatin, atorvastatin, rosuvastatin and pitavastatin are available in clinical practice in China. There are some differences in the cholesterol-lowering rate of different types and doses of statins, but the further reduction of LDL-C is only about 6% when the dose of any statin is multiplied, which is called the “6% effect of statin efficacy”. Statins can also reduce TG levels by 7%–30% and increase HDL-C levels by 5%–15%.

The clinical benefit of statin therapy comes primarily from a reduction in LDL-C levels. An individual assessment of overall cardiovascular risk and determination of treatment goals needs to be performed first, and patients are encouraged to participate in cardiovascular risk management decisions to select a statin regimen for patients that is expected to achieve LDL-C targets. If the target is not achieved based on the application of moderate-intensity statins, then combination therapy (combined with cholesterol absorption inhibitors and/or PCSK9 inhibitors) is considered. It should also be emphasized that the clinical status of the patient, the combination of drugs, drug tolerance and drug cost should be considered when deciding on the type and dose of statins.

Statins may be taken once daily at any time of day, but LDL-C reduction may slightly increase when taken at night. Statin application should be continued long-term after achieving the desired efficacy and discontinuation should be avoided if tolerated, with the goal of reducing the patient’s lifetime exposure to LDL-C. Some studies suggest that discontinuation of statins has the potential to increase cardiovascular events (Chen et al., 2007) If adverse effects such as increased liver enzymes occur after statin application, they can be managed by switching to a statin of another metabolic pathway, reducing the dose, taking it on alternate days (Li et al., 2012) or switching to a non-statin or a combination of a low-dose statin and a non-statin.

The results of the Cholesterol Treatment Investigators Collaborative Group analysis showed that in populations with different cardiovascular risk stratification, each 1 mmo1/L reduction in LDL-C after statin therapy was associated with a 20%–23% reduction in the relative risk of major cardiovascular events and a 10% reduction in all-cause mortality, while no increase in deaths from non-cardiovascular causes was seen (Cholesterol Treatment Trialists Collaboration, 2010). Existing studies have repeatedly demonstrated that the magnitude of the clinical benefit of statins in reducing ASCVD events is linearly and positively correlated with the magnitude of their LDL-C reduction. The magnitude of LDL-C reduction for different classes and doses of statins is shown in Table 11.

**TABLE 11:** Cholesterol-lowering intensity of statins.

Cholesterol-lowering intensity	Drugs and doses
High intensity (daily dose reduces LDL-C by ≥ 50%)	Atorvastatin 40–80 mg
	Rosuvastatin 20 mg
Moderate intensity (25%–50% reduction in LDL-C at daily dose)	Atorvastatin 10–20 mg
	Rosuvastatin 5–10 mg
	Fluvastatin 80 mg
	Lovastatin 40 mg
	Pitavastatin 1∼4 mg
	Pravastatin 40 mg
	Simvastatin 20–40 mg
	XZK 1.2 g

LDL-C: low-density lipoprotein cholesterol. Atorvastatin 80 mg is inexperienced in China, please use with caution.

Although XZK is classified as lipid-lowering Chinese medicine, its lipid-lowering mechanism is similar to that of statins. It is refined by adding special red currant to rice bio-fermentation, following the modern pharmaceutical production quality management standard. The main ingredients are 13 kinds of natural compound statins, which are lovastatin and its similar substances without crystal structure, and contain ergosterol, various trace elements and flavonoids. The common dose is 0.6g, 2 times/d. China coronary heart disease secondary prevention study, CCSPS and other clinical studies (Zhao et al., 2004; Collaborative Group on Xuezhikang for Secondary Prevention of Coronary Artery Disease, 2005; Lu et al., 2008; Li et al., 2010) confirmed that these capsules can lower LDL-C and significantly reduce total mortality, coronary heart disease mortality, and cardiovascular events in patients with coronary heart disease with few adverse effects.

Adverse effects of statins are a frequent concern in clinical practice. The most often reported so far include abnormal liver function, statin-related muscle complications, new-onset diabetes mellitus, and other adverse reactions.

Liver enzyme abnormalities are mainly manifested as elevated transaminases, with an incidence of about 0.5%–3.0% in a dose-dependent manner (McKenney et al., 2006; Bays et al., 2014). If liver enzyme abnormalities are considered to be caused by statins, the clinical management needs to be individualized, for example, if serum alanine aminotransferase and/or aspartate aminotransferase is elevated ≥3 times the upper limit of normal (ULN) and combined with elevated total bilirubin, the dose should be reduced or discontinued as appropriate. For those whose transaminases are elevated within 3 times the upper limit of normal, they can be observed on the basis of the original dose or dose reduction, or they can be switched to another metabolic pathway of statins, and some patients' transaminases can be normalized by this treatment. Loss of compensated cirrhosis and acute liver failure are contraindications to the use of statins.

Statin-associated muscle complications include myalgia, myositis, myopathy, and rhabdomyolysis (Maki et al., 2014; Stroes et al., 2015), with an incidence of 1%–5% (RCT results) or 5%–10% (observational study results), with rhabdomyolysis being rare. When muscle discomfort and/or weakness occurs during statin administration with or without CK elevation, other causes of the above situation should be identified and corrected first. If the clinical consideration is indeed statin-related muscle symptoms and the CK is progressively elevated by continuous testing, the statin dose should be reduced or discontinued, and the symptoms and CK level should be monitored regularly. When CK < 4×ULN, if there are no symptoms, consider continuing statin therapy and monitor closely; if there are symptoms, discontinue statins, monitor symptoms and CK levels, and consider restarting statins after symptoms disappear and CK returns to normal, and suggest switching to another metabolic pathway of statins. When CK > 4×ULN, it is recommended to stop the statin and closely monitor the symptoms and CK level; if CK > 10×ULN, it is necessary to be alert to the possibility of rhabdomyolysis, to detect the presence of hemoglobinuria and renal function impairment, and to immediately stop the statin and give hydration therapy and continuously monitor CK to normal level. For these patients it is recommended to combine drugs or switch to non-statin drugs.

Long-term use of statins has an increased risk of new-onset diabetes (Bays et al., 2014), with an incidence of about 9%–12%, a statin effect. Some studies have shown that pitavastatin has a low probability of causing new-onset diabetes (Vallejo-Vaz et al., 2015). The overall benefit of statins on ASCVD far outweighs the risk of new diabetes, and those with indications for statin therapy should adhere to such medications, whether they are at high risk for diabetes or diabetic patients.

The meta-analysis by the Cholesterol Treatment Investigators Collaborative Group of the Stroke Prevention by Aggressive Reduction in Cholesterol Levels (SPARCL) and Controlled Rosuvastatin Multinational Trial in Heart Failure (CORONA)showed a 21% increase in risk of hemorrhagic stroke for every 1 mmol/L reduction in LDL-C (Cholesterol Treatment Trialists Collaboration, 2010). Other meta-analyses have suggested that none of the LDL-C decreases significantly increased the risk of hemorrhagic stroke (Hackam et al., 2011; McKinney and Kostis, 2012). The overall benefits of statins for other stroke subtypes greatly outweigh the risks (Cholesterol Treatment Trialists Collaboration, 2010; Cholesterol Treatment Trialists Collaborators, 2012), but it remains to be explored which populations are susceptible to hemorrhagic stroke during statin therapy.

Other adverse effects of statins include headache, insomnia, depression, and gastrointestinal symptoms such as dyspepsia, diarrhea, abdominal pain, and nausea. In addition, the results of the meta-analysis showed no adverse effects of statins on renal function (Geng et al., 2014).

During the application of statins, it is necessary to pay attention to the interactions with other drugs. Statins are mostly metabolized by cytochrome (CY) P450 (including CYP3A4, CYP2C8, CYP2C9, CYP2C19, CYP2C6), which is the main metabolizing enzyme in the liver. When statins metabolized *via* the CYP3A4 pathway are combined with immune-suppressant drugs (e.g., cyclosporine, *etc.*), antifungal drugs, macrolides, calcium antagonists, other drugs (including amiodarone, gemfibrozil, *etc.*), and grapefruit juice (Wiggins et al., 2016), the risk of myopathy or rhabdomyolysis may be increased, so high-dose statins should be avoided and monitored for adverse effects.

Statin intolerance refers to the development of statin-related clinical adverse reactions and/or laboratory abnormalities after statin application and is generally defined as the simultaneous fulfillment of the following 4 conditions: 1) clinical presentation: subjective symptoms and/or abnormal objective blood tests; 2) inability to tolerate ≥2 statins, one of which is administered at the lowest dose; 3) presence of a causal relationship; and 4) exclusion of other causes (Li J J et al., 2020).

#### 10.1.2 Cholesterol absorption inhibitor

Cholesterol absorption inhibitors, such as ezetimibe and hyzetimibe inhibit the absorption of dietary and biliary cholesterol in the intestine by interacting with Niemann-Pick C1 at the level of the intestinal brush border without affecting the absorption of fat-soluble nutrients. Studies have demonstrated a further reduction in LDL-C levels of 18%–20% when ezetimibe is combined with a statin compared to placebo. The Improved Reduction of Outcomes Vytorin Efficacy International Trial (IMPROVE-IT) showed that the addition of ezetimibe to simvastatin in patients with ACS further reduced cardiovascular events (Cannon et al., 2015). The study of Heart and Renal Protection (SHARP) showed that the combination of ezetimibe and simvastatin improved the prognosis of ASCVD in patients with CKD (Sharp Collaborative Group, 2010). The recommended dose of ezetimibe is 10 mg/d, which can be taken in the morning or in the evening and is safe and well tolerated. No dose adjustment is required in patients with mild hepatic insufficiency or mild to severe renal insufficiency, and life-threatening hepatic failure is extremely rare (Phan et al., 2012). Adverse effects are mild and transient, mainly headache and gastrointestinal symptoms. Adverse effects such as increased transaminases and myalgia can also occur in combination with statins and are contraindicated during pregnancy and lactation.

Another cholesterol absorption inhibitor, hyzetimibe, is a new class I drug recently marketed in China, and its mechanism of action, usage and lipid-lowering efficacy are similar to ezetimibe (Ruan et al., 2014; Chen et al., 2015; Liao et al., 2021).

#### 10.1.3 proprotein convertase subtilisin/kexin type 9 (PCSK9) inhibitors

PCSK9 is a secreted serine protease synthesized by the liver that binds to and degrades LDLR, thereby reducing the clearance of serum LDL-C by LDLR. By inhibiting PCSK9, LDLR degradation can be prevented, and LDL-C clearance can be promoted. The main PCSK9 inhibitors that have been marketed are PCSK9 monoclonal antibodies. Inclisiran, a synthetic, double stranded, small interfering RNA (SiRna) has been approved for marketing in Europe and the United States

The mechanism of action of PCSK9 monoclonal antibodies is based on targeting the PCSK9 protein (Abifadel et al., 2003). PCSK9 antibodies bind to plasma PCSK9 and reduce the catabolism of LDLR on the cell surface, thereby reducing circulating LDL-C levels (Norata et al., 2014). There are currently two human monoclonal antibodies approved for marketing, evolocumab and alirocumab.

Studies have confirmed that evolocumab and alirocumab significantly reduce mean LDL-C levels by 50%–70%. A study evaluating cardiovascular outcomes of alirocumab for acute coronary syndromes was completed mainly in East Asian countries such as China (Alirocumab efficacy and safety vs. ezetimibe in high cardiovascular risk patients with hypercholesterolemia and on maximally tolerated statin in China, India, and Thailand, ODYSSEY EAST) randomized 615 patients with high-risk ASCVD with hyperlipidemia to treatment with alirocumab or ezetimibe for 6 months, reduced LDL-C by 56% and 20.3%, respectively (*p* < 0.0001) (Han et al., 2020). ODYSSEY EAST Chinese subgroup analysis showed that at week 24, the proportion of patients achieving LDL-C < 1.81 mmol/L (85.3% *versus* 42.2%) and <1.42 mmol/L (70.5% *versus* 17.0%) was significantly higher in the alirocumab group than in the ezetimibe group (both *p* < 0.001) (Han et al., 2022). In the study completed in China, evolocumab was found to significantly reduce LDL-C and other atherogenic lipid components in patients with type 2 diabetes combined with hyperlipidemia or mixed dyslipidemia treated with atorvastatin background, with good tolerability and no significant effect on glycemic indexes (Chen et al., 2019). Evolocumab and alirocumab are effective in the majority of patients including HeFH as well as HoFH patients with residual LDLR function, with those with receptor deficient HoFH responding poorly to treatment (Thedrez et al., 2018). Evolocumab reduced TG levels by 26% and increased HDL-C levels by 9%, with similar effects seen with alirocumab (Robinson et al., 2015; Stein and Turner, 2017). Both evolocumab and alirocumab reduced Lp(a) levels by about 30% (Gaudet et al., 2014; Cao Y X et al., 2019a). “Further Cardiovascular Outcomes Research With PCSK9 Inhibition in Subjects With Elevated Risk (FOURIER)” and “Evaluation of Cardiovascular Outcomes After an Acute Coronary Syndrome During Treatment With Alirocumab (ODYSSEY OUTCOMES) trial” suggested a 15% reduction in the relative risk of the primary cardiovascular event composite endpoint with PCSK9 monotherapy compared with placebo (Sabatine et al., 2015; Schwartz et al., 2018).

Evolocumab 140 mg or alirocumab 75 mg administered subcutaneously once every 2 weeks was safe and well tolerated, with the most common side effects including injection site itching and flu-like symptoms (Cicero et al., 2014). The Evaluating PCSK9 Binding Antibody Effects on Cognitive Health in High Cardiovascular Risk Subjects Influence on Cognitive Health in High Cardiovascular Risk Subjects (EBBINGHAUS) trial (Giugliano et al., 2017) did not find an effect of PCSK9 monoclonal antibodies on neurocognitive function.

Although the application of PCSK9 monoclonal antibodies often reduces LDL-C to lower levels in patients, the duration of application regarding PCSK9 monoclonal antibodies is a clinical concern. The latest FOURIER-Open label Extension (FOURIER-OLE) study suggests that ASCVD patients on evolocumab for up to 8.4 years (median 5 years) with a median LDL-C level of 0.78 mmol/L had serious adverse events, muscle-related events, new-onset diabetes mellitus The incidence of adverse events such as hemorrhagic stroke and neurocognitive events was similar to that of the placebo group (O'Donoghue et al., 2022).

Inclisiran is a PCSK9 small interfering RNA, and studies have shown that its LDL-C reduction rate is comparable to that of PCSK9 monoclonal antibodies while its effects are longer lasting, and the efficacy of one injection can be maintained for 6 months (Fitzgerald et al., 2017), which is an ultra-long-acting PCSK9 inhibitor. Increasing patient compliance with treatment is its main advantage. It has been approved for use in primary hypercholesterolemia patients in Europe and the United States. ORION-4, a large-scale Phase III international multicenter RCT of inclisiran with cardiovascular outcomes as the primary endpoint, is currently underway.

#### 10.1.4 Probucol

Probucol affects lipoprotein metabolism by incorporating into the core of LDL particles, making LDL easy to be cleared by non-receptor pathways. The commonly used dose is 0.5g/dose, 2 times/d. It is mainly indicated for patients with FH, especially HoFH and Achilles’ tendon xanthomas, and has the effect of reducing cutaneous xanthomas (Yamashita et al., 2008). Common adverse reactions are gastrointestinal reactions, which can also cause dizziness, headache, insomnia, and rash, *etc.* An extremely rare serious adverse reaction is QT interval prolongation. Ventricular arrhythmias, prolonged QT interval, and low potassium are contraindicated. Currently, it is mainly used in combination with other lipid-lowering drugs for the treatment of patients with FH to reduce the occurrence and severity of cutaneous xanthomas.

#### 10.1.5 Bile acid chelators

Bile acid chelators are alkaline anion exchange resins that block the reabsorption of cholesterol from bile acids in the intestine (Knapp et al., 2001). Clinical use: cauleenamine 5 g/d, 3 times/d; cauletipo 5 g/d, 3 times/d; cauleveram 1.875 g/d, 2 times/d. Combined with statins, it can significantly improve lipid-lowering efficacy. Common adverse reactions include gastrointestinal discomfort, constipation, and interference with the absorption of certain drugs. Absolute contraindications for these drugs are abnormal β-lipoproteinemia and serum TG > 4.5 mmol/L.

#### 10.1.6 Other lipid-lowering drugs

Zhibitai is a compounded preparation of red currant and Chinese herbs (hawthorn, zedoary, atractylodes). The commonly used dose is 0.24–0.48 g/d, 2 times/d, which has cholesterol-lowering effect (Xu et al., 2010a; Xu et al., 2010b). Adverse effects of this drug are rare. Polyhexanol is a mixture of eight higher fatty primary alcohols purified from sugarcane wax, commonly used at doses of 10–20 mg/d, with a weak and slow onset of lipid-lowering action and rare adverse effects (Hu, 2008; Liu et al., 2012).

### 10.2 Major triglyceride-lowering drugs

#### 10.2.1 Fibrates

Fibrates reduce serum TG levels and increase HDL-C levels by activating PPARα and activating LPL (Rubins et al., 1999; Chu et al., 2002; Keech et al., 2005; Accord Study Group et al., 2010; Jun et al., 2010). Commonly used fibrates are (with extended-release formulations): fenofibrate tablets 0.1 g/d, 3 times/d; micronized fenofibrate 0.2 g/d, 1 time/d; benzofibrate 0.2 g/d, 3 times/d; benzofibrate extended-release tablets 0.4 g/d, 1 time/d; and gemfibrozil 0.6 g/d, 2 times/d. Common adverse effects are similar to those of statins and include liver, muscle, and nephrotoxicity The incidence of elevated serum CK and alanine aminotransferase levels were <1%. The results of clinical trials and meta-analyses suggest that fibrates can significantly lower TG and increase HDL-C, but the cardiovascular benefit is uncertain, with only subgroups of patients with elevated TG combined with low HDL-C suggesting improved cardiovascular prognosis (Keech et al., 2005; Accord Study Group et al., 2010; Jun et al., 2010).

Pemafibrate is a novel PPARα agonist that modulates PPARα expression by selectively binding to PPARα receptors, thereby reducing serum TG levels (Fruchart, 2017). It is used for the treatment of hypertriglyceridemia in adults. The recommended dose is 0.1–0.2 mg twice/d. The PROMINENT study, a large international multicenter RCT of pemafibrate with cardiovascular outcomes as the primary endpoint, was terminated early due to futility, presumably related to its concurrent elevation of LDL-C (12.3%) and ApoB (4.8%) (Das Pradhan et al., 2022).

#### 10.2.2 High purity omega-3 fatty acids

Omega-3 fatty acids reduce serum TG concentrations by reducing TG synthesis and secretion and TG incorporation into VLDL and enhancing TG clearance from VLDL particles (Leaf and Weber, 1988). Studies have shown that omega-3 fatty acids (4 g/d) reduce TG levels by approximately 20%–30% and ≥30% in patients with TG of 2.3–5.6 mmol/L and ≥5.6 mmol/L, respectively (Skulas-Ray et al., 2019), and omega-3 fatty acid products of different composition have similar efficacy in reducing TG (Kelley and Adkins, 2012) and are mainly used for the treatment of hypertriglyceridemia (Harris, 1996; 1997). Omega −3 fatty acid carboxylic acid preparations (containing DHA and EPA), and omega-3 fatty acid ethyl esterified preparations (containing DHA and EPA, and EPA-only IPE), are approved by the US FDA for use in adult patients very high TG (≥5.6 mmol/L).

The results of the REDUCE-IT study showed that IPE 4 g/d further reduced the relative risk of major adverse cardiovascular events by up to 25% on top of statins (Bhatt et al., 2019). The US Food and Drug Administration (FDA) has approved IPE for cardiovascular risk reduction indications and is currently applying for marketing approval in China. A meta-analysis suggests that omega-3 fatty acids containing EPA as well as DHA may also reduce cardiovascular events, but with less benefit than IPE (Khan et al., 2021). The latest randomized trial Evaluation in Secondary Prevention Efficacy of Combination Therapy - Statin and EPA (RESPECT-EPA) suggests that for patients with chronic stable coronary artery disease, the difference in the reduction of the primary endpoint-MACE by statins combined with highly purified EPA (1.8 g/d) was close to statistically significant (*p* = 0.055), but the difference in the reduction of the secondary endpoint-compound risk of coronary events was statistically significant (*p* = 0.031), suggesting that EPA has some coronary vascular protective effect (Nishizaki et al., 2022).

#### 10.2.3 Niacin analogs

Niacin analogs have been shown to lower TC, LDL-C and TG and raise HDL-C at high doses. The lipid-lowering effects are associated with inhibition of hormone-sensitive Lipase activity in adipose tissue, resulting in reduction of free fatty acid entry into the liver, and reduction of VLDL secretion. The most common adverse effect is flushing of the face; others include pruritus, rash, liver damage, hyperuricemia, hyperglycemia, acanthosis, and gastrointestinal discomfort, and are contraindicated in chronic active liver disease, active peptic ulcer, and severe gout. 2 large RCTs (Lavigne and Karas, 2013; Hps Thrive Collaborative Group, 2014) of nicotinic acid analogs (one with extended-release nicotinic acid analogs and the other with the nicotinic acid analog galaropilan) did not show cardiovascular benefit, and the adverse effects were increased.

### 10.3 New lipid-lowering drugs

Lipid-lowering drugs that act on new targets continue to emerge. Among them, lomitapide, a microsomal TG transfer protein inhibitor, and mipomersen, an ApoB100 synthesis inhibitor, were approved by the US FDA for the treatment of FH as early as 2012 and 2013 (Raal et al., 2010; Cuchel et al., 2013), but have not yet entered the Chinese market. In recent years, several new lipid-lowering drugs have been approved or pending approval for clinical use abroad, but none of them have yet been marketed in China (Table 12).

**TABLE 12:** New lipid-lowering drugs AND Combined application of lipid-lowering drugs.

New lipid-lowering drugs			
Name	Lipid lowering target	Main lipid-lowering mechanism	Indication (or proposed approval)
Lomitapide	Microsomal triglyceride transfer protein	Reduce VLDL production	≥18 years old HoFH
Mipomersen	ApoB100	Reduce VLDL production	≥12 years old HoFH
Bempedoic acid	ATP citrate lyase	Facilitate VLDL and LDL metabolism	HeFH, ASCVD
Evinacumab	Angiopoietin-like 3	Facilitate VLDL and LDL metabolism	≥12 years old HoFH
Volanesorsen	ApoC3	Facilitate CM and VLDL metabolism	≥18 years old FCS
Pelacarsen	Apo(a)	Reduce Lp(a) generation	ASCVD with increased Lp(a)

Combined application of lipid-lowering drugs			
Joint strategy^a^	Applicable situation	Rate of lipid reduction or MACE	Safety concerns
Statin + cholesterol absorption inhibitor	LDL-C does not meet the standard with single drug	LDL-C 50%–60%	Routine monitoring
Statin + PCSK9 monoclonal antibody	LDL-C does not meet the standard with single drug	LDL-C ≈75%	Routine monitoring
Statin + cholesterol absorption inhibitor + PCSK9 monoclonal antibody	LDL-C does not meet the standard with single drug	LDL-C ≈85%	Routine monitoring
Statin + high purity IPE 4 g/d	LDL-C meets the standard, TG 2.3–5.7 mmol/L	25% reduction in MACE risk	Atrial fibrillation, bleeding
Statin + fenofibrate or omega-3 fatty acids	LDL-C meets the standard, TG 2.3–5.7 mmol/L	Reduction in MACE risk	Renal function, atrial fibrillation, bleeding
Fibrates + omega-3 fatty acid	TG ≥ 5.7 mmol/L with single drug	TG 60.8%–71.3%	Routine monitoring
Fibrates + niacin analogs	TG ≥ 5.7 mmol/L with single drug	Lack of data	Routine monitoring
Omega-3 fatty acid + niacin analogs	Fibrates intolerance and TG ≥ 5.7 mmol/L with single drug	TG>33%	Routine monitoring

^a^ Statin in the combination strategy refers to medium-intensity statins (see Table 10 for specific types and doses), and “omega-3, fatty acids” refer to medical prescription grade at 4 g/d.

Apo: apolipoprotein; ATP: adenosine triphosphate; VLDL: very low-density lipoprotein; LDL: low-density lipoprotein; CM: chylomicron; Lp(a): lipoprotein(a); HoFH: homozygous familial hypercholesterolemia; HeFH: heterozygous familial hypercholesterolemia; ASCVD: atherosclerotic cardiovascular disease; FCS: familial chylomicronemia syndrome; PCSK9: preprotein convertase chymotrypsin 9; IPE: icosapent ethyl; LDL-C: low-density lipoprotein cholesterol. TG: triglycerides; MACE: major adverse cardiovascular events.

#### 10.3.1 Adenosine triphosphate citrate lyase inhibitor

Bempedoic acid, an adenosine triphosphate citrate lyase inhibitor, is also a cholesterol synthesis inhibitor. Clinical trial data show that bempedoic acid reduces LDL-C by about 30% when used orally alone, and further reduces LDL-C by 17%–22% when combined with a statin, and further reduces LDL-C by 28.5% when combined with ezetimibe, for a total reduction of 48%, with good overall safety and tolerability (Goldberg et al., 2019; Ray et al., 2019). Bempedoic acid alone and a fixed combination tablet of bempedoic acid/ezetimibe (180/10 mg) have been marketed overseas for the treatment of patients with heterozygous FH or ASCVD who do not meet LDL-C targets (Ballantyne et al., 2018).

#### 10.3.2 Angiopoietin-like 3 inhibitor

Angiopoietin-like 3 is a key protein that regulates VLDL metabolism by inhibiting LPL activity. Phase II and III clinical trials with the angiopoietin-like 3 human monoclonal antibody evinacumab enrolled HoFH patients and showed that evinacumab further reduced LDL-C by nearly 50% in HoFH patients on top of existing lipid-lowering therapy (Raal et al., 2020). It is approved in the United States for use in children ≥12 years of age or adults with HoFH at the recommended dose of 15 mg/kg IV infusion once every 4 weeks.

#### 10.3.3 Apolipoprotein C3 inhibitor

ApoC3 is a key Apo that regulates CM and VLDL metabolism by inhibiting LPL and hepatic lipase activity. Phase III clinical trial data for ApoC3 2nd generation antisense oligonucleotide volanesorsen showed a TG reduction of up to 77% but was not approved by the US FDA due to a 48.5% reduction in platelet count (<100,000/mL). It was approved for marketing by the European Medicines Agency, but only for the treatment of adult patients with familial chylomicronemia syndrome in whom diet and other lipid-lowering drugs were not effective (Witztum et al., 2019).

#### 10.3.4 New Lipoprotein(a)-lowering drugs

New Lp(a)-lowering drugs include Apo(a) antisense oligonucleotide (pelacarsen) and Apo(a) small interfering RNA (SLN360), both of which have shown significant Lp(a) reduction up to 98% in Phase I clinical trials (Nissen et al., 2022). Pelacarsen’s Phase II trial included 286 patients with ASCVD with Lp(a) ≥ 60 mg/dL and confirmed its clear Lp(a) lowering efficacy and good safety profile (Tsimikas et al., 2020). HORIZON, a large-scale, international, multicenter clinical study, to assess the impact of lipoprotein(a) lowering with pelacarsen on major cardiovascular events in patients with known cardiovascular disease is currently underway.

### 10.4 Combinations of lipid-lowering drugs

The combination application of lipid-lowering drugs is an important trend in dyslipidemia intervention strategies, with the main aim of improving lipid target rates, further reducing cardiovascular risk, and reducing the incidence of adverse effects of lipid-lowering drugs. The main combination application options currently available are as follows (Table 12).

#### 10.4.1 Combination of lipid-lowering drugs to reduce cardiovascular risk

##### 10.4.1.1 Statins in combination with cholesterol absorption inhibitors

These two classes of drugs affect the synthesis and absorption of cholesterol separately and can produce good synergistic effects. The RCT meta-analysis showed that the combination of ezetimibe with different types of statins resulted in a further decrease in LDL-C by 15%–23% compared to statins alone, and the combination of ezetimibe with medium-to high-intensity statins resulted in an LDL-C decrease of >50% without increasing the adverse effects of statins (Morrone et al., 2012; Masana et al., 2015). The IMPROVE-IT and SHARP studies showed that the combination of statins and ezetimibe in patients with ACS and CKD, respectively, significantly reduced cardiovascular events (Sharp Collaborative Group, 2010; Cannon et al., 2015). Hybutimibe/hyzetimibe is a cholesterol absorption inhibitor developed in China, and data of the Chinese population show that hybutimibe/hyzetimibe 10 mg/d day alone can reduce LDL-C by about 15% (compared to placebo) (Qi et al., 2022b), and 20 mg/d combined with statins further reduces LDL-C by about 16% compared to statins alone, with good safety and tolerability (Qi et al., 2022a).

##### 10.4.1.2 Statins in combination with PCSK9 inhibitors

PCSK9 inhibitors increase plasma LDL clearance by reducing LDLR degradation and increasing LDLR quantity, complementing and synergizing with statins and cholesterol uptake inhibitors in terms of lipid-lowering mechanisms. The results of the FOURIER study and the ODYSSEY OUTCOMES study showed that the combination of evolocumab with statins ( ± ezetimibe) further reduced LDL-C by up to 59% and the combination of alirocumab reduced LDL-C by 55%, both of which significantly reduced the relative risk of MACE by 15% (Sabatine et al., 2017; Schwartz et al., 2018). This combination strategy resulted in rapid LDL-C target achievement, good overall safety and tolerability, and strong evidence of cardiovascular benefit.

##### 10.4.1.3 Statin in combination with high purity IPE

The results of the REDUCE-IT study showed that the combination of high-purity IPE 4 g/d significantly reduced the relative risk of MACE by up to 25% in ASCVD patients with mild-to-moderate TG elevation or in diabetic patients with at least 1 cardiovascular risk factor who were already on statin therapy with LDL-C at target (Bhatt et al., 2019). Therefore, this combination can be used to further reduce ASCVD risk in patients with LDL-C <2.6 mmol/L after statin therapy but with mild-to-moderate elevations in TG, and its regimen does not increase the respective adverse effects overall. However, there is some risk of bleeding and new-onset atrial fibrillation with EPA 4 g/d, as well as increased caloric intake in diabetic and obese patients, and the choice of this regimen should be weighed on an individual basis.

##### 10.4.1.4 Others

Lipid-lowering traditional Chinese medicine combined with statins or ezetimibe: Chinese single and multicenter RCTs as well as RCT meta-analyses showed that the LDL-C-lowering efficacy of statins combined with Zhibitai was comparable to that of high-dose statins alone, with better safety (Zhao et al., 2017; Xu et al., 2018; Li M et al., 2020); Chinese small-scale clinical trials confirmed the effectiveness and safety of the LDL-C-lowering combination of XZK and ezetimibe (Guo et al., 2021).

Statins in combination with fibrates or high-purity omega-3 fatty acids: The cardiovascular benefit of statins in combination with fibrates or high-purity omega-3 fatty acids (containing EPA and DHA) is controversial. The Action to Control Cardiovascular Risk in Diabetes (ACCORD) subgroup study suggests that the combination of fenofibrate may further reduce cardiovascular risk in diabetic patients treated with statin therapy who basically meet LDL-C target but have TG > 2.3 mmol/L and HDL-C < 0.9 mmol/L (*p* = 0.057) (Accord Study Group et al., 2010; Elam et al., 2017). However, the newly published PROMINENT study failed to confirm a further benefit of statin in combination with pemafibrate in a similar population (Das Pradhan et al., 2022). In addition, the safety of combining statins with fenofibrate in the Chinese population is acceptable, but the safety of longer-term combinations remains to be further validated (Ren et al., 2005; Zhao et al., 2016). The risk of myopathy is relatively high with the combination of gemfibrozil and statins, and it is recommended to avoid the combination of the two as much as possible.

#### 10.4.2 Combination of lipid-lowering drugs in severe hypertriglyceridemia

When TG is severely elevated (≥5.6 mmol/L) and TG levels are not well controlled by lifestyle and single lipid-lowering drugs, a combination of two or more between fibrates, high-dose (2–4 g/d) high-purity omega-3 fatty acids, and niacin analogs may be used (Roth et al., 2009; Shearer et al., 2012). The combination of high-purity omega-3 fatty acids and niacin basically does not further increase the risk of hepatic and renal safety of fibrates alone. Common adverse effects include gastrointestinal reactions, bleeding, atrial fibrillation (positively correlated with the applied dose of omega-3 fatty acids), and facial flushing (correlated with niacin).

## 11 Other measures for lipid-lowering treatment

Key points.

Lipoprotein apheresis, liver transplantation, partial ileal bypass surgery and portal vein bypass are used as adjuvant therapeutic measures in patients with FH. The results of lipoprotein plasma replacement are positive.

### 11.1 Lipoprotein apheresis

Lipoprotein apheresis (LA) is an important adjuvant therapy for patients with FH, especially HoFH, and can reduce LDL-C levels by 55%–70% (Cuchel et al., 2014; Zhao et al., 2022). UK and German guidelines recommend LA for patients with progressive coronary artery disease with Lp(a) > 150 nmol/L (Heigl et al., 2015; Cegla et al., 2019). Long-term treatment may result in regression of cutaneous xanthomas. The optimal frequency of treatment is once a week, but every 2 weeks is commonly used. LA can still be applied during pregnancy. However, as the treatment is expensive, time-consuming and with risk of infection, application of this treatment is limited in clinical use, especially in China. Adverse effects include hypotension, abdominal pain, nausea, hypocalcemia, iron deficiency anemia, and anaphylactic reactions, but the incidence is low.

### 11.2 Liver transplantation and surgery

Liver transplantation can result in significant improvement in LDL-C levels and is advocated before cardiovascular involvement to avoid cardiovascular complications. Although liver transplantation alone or combined with heart transplantation is a successful treatment strategy, it is rarely used clinically because of the high number of post-transplant complications and mortality, lack of donors, and the need for lifelong immunosuppressive drugs. Previously reported partial ileal bypass surgery and portal vein bypass can also be applied in the treatment of patients with very severe HoFH, but few existing guidelines or consensus recommend it (Harada-Shiba et al., 2018a).

## 12 Lipid management for specific populations

Key points.

Specific populations are patients with certain coexisting diseases (e.g., hypertension, diabetes, CKD, stroke), special physiological states (pregnancy), children, elderly people of advanced age, and special lipid metabolism abnormalities (familial hypercholesterolemia). Their lipid metabolism status and response to drug therapy are somewhat specific, so a more individualized lipid management strategy is needed.

### 12.1 Hypertension

Hypertension is an important risk factor for atherosclerosis, and both endothelial cell dysfunction and intimal thickening in arteries with hypertension can accelerate the development of atherosclerosis. There are three studies that specifically address the primary prevention of hypertension. The Anglo-Scandinavian Cardiac Outcomes Trial - Lipid Lowering Arm (ASCOT-LLA) included hypertensive patients with a high risk of coronary heart disease, and compared with placebo, atorvastatin reduced ASCVD endpoint events by 35%, and mean LDL-C was reduced to 2.2 mmol/L in the treatment group (Sever et al., 2003). The Heart Outcomes Prevention Evaluation (HOPE)-3 study enrolled hypertensive patients at moderate risk for ASCVD and compared to placebo, rosuvastatin reduced ASCVD endpoint events by 24% and the mean LDL-C was reduced to 2.4 mmol/L in the treatment group (Yusuf et al., 2016a; Yusuf et al., 2016b). The lipid-lowering trial component of the Antihypertensive and Lipid-Lowering Trial to Prevent Heart Attack Trial (ALLHAT-LLT) included patients with moderate hypertension combined with hypercholesterolemia, and compared with placebo, pravastatin failed to significantly reduce the risk of ASCVD, with a 17% reduction in LDL-C in the treatment group and a mean of 3.1 mmol/L (ALLHAT Officers and Coordinators for the ALLHAT Collaborative Research Group, and the Antihypertensive and Lipid-Lowering Treatment to Prevent Heart Attack Trial, 2002). Most secondary prevention study populations with statins alone or in combination with non-statin lipid-lowering drugs contained varying proportions of hypertensive patients, and they all benefited significantly from intensive lipid lowering. Therefore, the presence or absence of hypertension is specifically listed when performing a population ASCVD risk assessment (Figure 1) to emphasize the importance of lipid management in patients with hypertension. The corresponding LDL-C target values for hypertensive individuals should be determined according to risk stratification and treated with cholesterol-lowering therapy (Hypertensive Group of Chinese Society of Cardiology of Chinese Medical Association and Editorial Board of Chinese Journal of Cardiology, 2021).

### 12.2 Diabetes

Diabetes is an important independent risk factor for ASCVD, and some studies suggest that dyslipidemia has the greatest impact on ASCVD risk in diabetic patients. Dyslipidemia in diabetic patients is characterized by elevated TG, reduced HDL-C, and normal or mildly elevated LDL-C. However, their LDL particles have small and dense characteristics, which have stronger atherogenic effects. Diabetes combined with high TG reveals an elevated proportion of TRL cholesterol, and the use of LDL-C as a lipid-lowering target at this time may underestimate the patient’s ASCVD risk, whereas non-HDL-C contains both LDL-C and TRL cholesterol, which can better reflect the patient’s atherogenic lipoprotein profile. Therefore, both LDL-C and non-HDL-C are recommended as lipid-lowering targets in diabetic patients. Patients ≥40 years of age with diabetes (especially “Long Duration” ≥10 years for type 2 diabetes mellitus) are considered high risk, and type 1 diabetes duration ≥20 years can be considered high risk. In addition, Albuminuria ≥30 mcg of albumin/mg creatinine, Retinopathy, Neuropathy, and ABI <0.9, may indicate important organ damage. While diabetic patients <40 years of age should have ASCVD risk determined in combination with other ASCVD factors (hypertension, smoking, HDL-C) and/or target organ damage; patients should also be considered high risk for ASCVD if they have ≥3 risk factors or combined target organ damage. For diabetic patients with moderate or low risk of ASCVD, all should control LDL-C below 2.6 mmol/L (Supplementary Table S4) (Cholesterol Treatment Trialists Collaborators, 2008).

Numerous studies of primary and secondary lipid-lowering interventions for prevention have shown that statins significantly reduce the risk of ASCVD in patients with diabetes. Studies of statins combined with cholesterol absorption inhibitors or/and PCSK9 monoclonal antibodies have shown that ASCVD patients with comorbid diabetes benefit more from intensive lipid lowering. The results of studies with statin-based combinations of fibrates were neutral overall, but a subgroup with combined elevated TG and reduced HDL-C could benefit from fenofibrate therapy. The results of intervention studies of statins combined with high-purity omega-3 fatty acids on ASCVD risk in diabetic patients were inconsistent, with 4 g IPE daily significantly reducing ASCVD risk (Bhatt et al., 2019). Therefore, it is recommended that patients at high risk of diabetes choose statins as the basic lipid-lowering therapy, and if LDL-C is not achieved, a combination of cholesterol absorption inhibitor or PCSK9 inhibitor is required (Cannon et al., 2015; Sabatine et al., 2017). If the LDL-C standard is met and TG is still increased or non-HDL-C is not met, the combination of high-purity IPE, omega-3 fatty acids or fibrates is considered (Keech et al., 2005; Accord Study Group et al., 2010; Bhatt et al., 2019).

### 12.3 Chronic kidney disease

The risk of death is significantly higher in CVD patients with combined CKD. Patients with CKD stage 3–5 are directly classified as high risk for ASCVD. These patients are further characterized by a significant increase in TG, a decrease in HDL-C and a significant increase in sdLDL particles. Patients with CKD have significantly higher Lp(a) levels due to the effect on Lp(a) metabolism. However, the reduction of ASCVD risk by statin therapy was affected by the patients' renal function status. In patients with mild to moderate renal insufficiency, statin therapy significantly reduced their CVD risk (Baigent et al., 2011; Cholesterol Treatment Trialists Collaboration, 2016). However, in patients with severe renal insufficiency, two studies of statin interventions in patients with severe CKD treated with dialysis failed to show an effect (Supplementary Table S4) (Wanner et al., 2005; Fellstrom et al., 2009).

### 12.4 Stroke

In secondary prevention of ischemic stroke, statins are associated with a 12% reduction in the risk of stroke recurrence for every 1 mmol/L reduction in LDL-C, as well as a reduction in the risk of myocardial infarction and cardiovascular death (Amarenco et al., 2006). A statin meta-analysis showed a 21.1% reduction in the risk of stroke for every 1 mmol/L reduction in LDL-C (Amarenco and Labreuche, 2009). A stratified analysis of the IMPROVE-IT study showed that in ACS patients with a combined history of stroke, combining cholesterol absorption inhibitor based on statins significantly reduced the risk of ischemic stroke recurrence by 48% and the risk of all types of stroke recurrence by 40% compared with statins alone (Bohula et al., 2017b). In the FOURIER study and the ODYSSEY-OUTCOMES study, evolocumab and alirocumab reduced the risk of stroke by 25% and 27%, respectively, in ASCVD patients compared with the control groups (Sabatine et al., 2017; Schwartz et al., 2018).

There is disagreement about the relationship between lipid-lowering therapy and cerebral hemorrhage; in the SPARCL study in patients with ischemic stroke, 80 mg atorvastatin treatment significantly reduced fatal or non-fatal stroke by 16%, but with a mildly increased risk of cerebral hemorrhage. Overall, the benefits of LDL-C reduction far outweighed the harms of potential hemorrhagic stroke (Supplementary Table S5) (Amarenco et al., 2006; Amarenco and Labreuche, 2009).

### 12.5 Elderly people aged 75 and above

There is limited evidence for statins in elderly patients aged ≥75 years. The Prospective Study of Pravastatin in the Elderly at Risk (PROSPER) was conducted in elderly patients aged 70–82 years, 50% of whom were at high risk for ASCVD and 50% had ASCVD. The results showed a significant 15% reduction in MACE events in the pravastatin 40 mg group compared to placebo, providing partial evidence for primary prevention with statins in older patients (Shepherd et al., 2002). A recent meta-analysis of 28 statin studies showed that all 4 age groups, ≥75 years and <75 years, benefited from statin therapy, but there was a downward trend in the benefit in the older age group (70–75 years, ≥75 years) without concomitant ASCVD (Cholesterol Treatment Trialists Collaboration, 2019). A stratified analysis of the IMPROVE-IT study showed that in older patients with ACS aged 75 years and older, statin combined with ezetimibe had a 20% lower risk of cardiovascular events than statins alone (Bach et al., 2019). The age range of patients enrolled in the two secondary prevention studies of PCSK9 inhibitors was 40–80 years (Sabatine et al., 2017) and ≥18 years (Schwartz et al., 2018), respectively, both of which included those ≥75 years, and a stratified analysis in the FOURIER study showed that those ≥69 years benefited from lipid-lowering therapy with PCSK9 monoclonal antibodies in consistent with those <69 years (Sever et al., 2021). Overall, this evidence suggests that ASCVD patients ≥75 years of age can be treated with the same lipid-lowering principles as patients <75 years of age.

A primary prevention study of ezetimibe in elder patients ≥75 years of age (Ezetimibe Lipid-Lowering Trial on Prevention of Atherosclerotic Cardiovascular Disease in 75 or Older, EWTOPIA 75) showed that patients ≥75 years of age without coronary artery disease taking 10 mg of ezetimibe significantly reduced the risk of sudden cardiac death, myocardial infarction, coronary revascularization, and stroke by 34%. This supports ezetimibe as a safe and effective primary prevention of ASCVD in the elderly (Ouchi et al., 2019).

Special attention is paid to the elderly aged ≥75 years who often suffer from a variety of chronic diseases and need to take a variety of drugs, drug interactions and adverse reactions should be aware. Most of the senior patients have different degrees of hepatic and renal decompensation, and the selection of lipid-lowering drug dose needs to be individualized. The starting dose should not be too high, and the dose of lipid-lowering drugs should be adjusted according to the treatment effect. The liver and kidney function and CK should also be monitored. For primary prevention of ASCVD in elderly people aged ≥75 years, in addition to considering the risk of ASCVD development, it is also necessary to assess the functional status, cognitive status, co-morbidities, multiple drug use and other geriatric syndrome characteristics, and more individualized selection is needed to achieve the lifelong benefit goal of extending healthy life expectancy and shortening disability life. Because there is no RCT of statin treatment targets in elderly patients, no specific recommendation is made for statin treatment targets in elderly patients (Supplementary Table S5).

Results from a new multicenter observational study of acute myocardial infarction in elderly Chinese (≥80 years) (Liu et al., 2022) showed that patients with baseline LDL-C levels <70 mg/dL did not provide further cardiovascular benefit compared with patients with LDL-C ≥70 mg/dL, and there was a trend toward increased primary endpoint events during follow-up, suggesting that the optimal intervention cut point for LDL-C in elderly patients needs further study.

### 12.6 Pregnancy

Pregnancy leads to physiologic elevation of lipids, with LDL-C rising between 40% and 50% in healthy pregnant women and TG tending to rise around 14 weeks of gestation. The effects of hyperlipidemia on pregnancy are related to the composition and severity of the elevated lipids. The harms associated with hypercholesterolemia arise slowly, but severe hypertriglyceridemia can lead to acute pancreatitis and maternal mortality of up to 20% (Jacobson et al., 2015b; Mehta et al., 2020).

For lipid management in pregnancy the focus is on screening. The drug options are very limited. Statins are not usually recommended for patients with hypercholesterolemia and there is no evidence for cholesterol absorption inhibitors or PCSK9 inhibitors. Bile acid sequestrants may be considered for patients with combined ACS in pregnancy, and LA therapy may be considered for familial hypercholesterolemia combined with ASCVD. High purity omega-3 fatty acids may be considered for patients with severe hypertriglyceridemia (>5.6 mmol/L). For severe hypertriglyceridemia, fibrates may be used with caution. LA therapy may be considered for patients with combined pancreatitis (Jacobson et al., 2015b; Mehta et al., 2020).

Data on statins causing fetal malformations are mainly from animal studies and case reports, with most of the case reports being about fat-soluble statins. Two recent randomized controlled studies of pravastatin and cohort studies did not find an increased risk of birth defects with statin therapy (Costantine et al., 2021; Dobert et al., 2021; Yang et al., 2022). A meta-analysis has suggested that statins do not increase the risk of birth defects but are associated with an increased risk of spontaneous abortion (Vahedian-Azimi et al., 2021a; Vahedian-Azimi et al., 2021b). Therefore, on 20 July 2021, the US FDA ordered the removal of the “pregnancy category X″ label for statins (category X is prohibited) based on new data on the safety of statins during pregnancy (Mauricio and Khera, 2022). However, patients who are pregnant with high or very high-risk features for ASCVD, such as FH or previous acute coronary events, may choose statins with caution based on a multidisciplinary consultation and a full assessment of the pros and cons.

### 12.7 Children and adolescents

The incidence of dyslipidemia in children and adolescents in China is on the rise, with detection rates as high as 20.3%–28.5% due to changes in dietary patterns, reduced physical activity, and poor lifestyles brought about by economic and social development. There is a lack of unified standards for dyslipidemia in children and adolescents, and according to the standards for dyslipidemia in children in the United States (Elkins et al., 2019), national standards for screening and defining dyslipidemia in children and adolescents in China have been proposed (The Editorial Board of Chinese Journal of Pediatrics et al., 2009; The Subspecialty Group of Rare Diseases et al., 2022).

#### 12.7.1 Blood lipid screening

Lipid screening is recommended for children and adolescents with the following conditions.

1) Myocardial infarction, angina pectoris, stroke, CABG, stent placement, angioplasty, sudden death in a first- or second-degree relative <65 years of age in females or <55 years of age in males;
2) Parent with TC ≥ 6.2 mmol/L or known history of lipid abnormalities;
3) Having cutaneous xanthomas or tendon xanthomas or lipid corneal arches;
4) Having diabetes, hypertension, obesity (2–8 years) or overweight (12–16 years) or smoking behavior; dyslipidemia genetic screening should be performed for subjects with suspected FH.

#### 12.7.2 Dyslipidemia criteria

Chinese reference standards (The Subspecialty Group of Rare Diseases et al., 2022) for lipid screening and dyslipidemia in children and adolescents are shown in Supplementary Table S6 (Harada-Shiba et al., 2018b; Raal et al., 2018; Ishigaki et al., 2019; Lee et al., 2019; Luirink et al., 2019; Ramaswami et al., 2019; Vuorio et al., 2019; Di Taranto et al., 2021; The Medical Letter, 2021; Watts et al., 2021; Zhao et al., 2021).

#### 12.7.3 Dyslipidemia intervention

In children with dyslipidemia, lifestyle including exercise and diet is the cornerstone of dyslipidemia treatment. No less than 1 h of moderate to vigorous exercise per day and no more than 2 h of sedentary time per day are recommended. Dietary intervention plays an important role for the treatment of dyslipidemia in children and adolescents, where mild to moderate dyslipidemia can be normalized, even in HoFH. Dietary intervention should improve dyslipidemia, while ensuring adequate nutritional intake without affecting growth and development. Pharmacological treatment can refer to the relevant consensus (The Subspecialty Group of Rare Diseases et al., 2022). Those with suspected FH refer to the FH section.

### 12.8 Familial hypercholesterolemia

**FIGURE 2:**
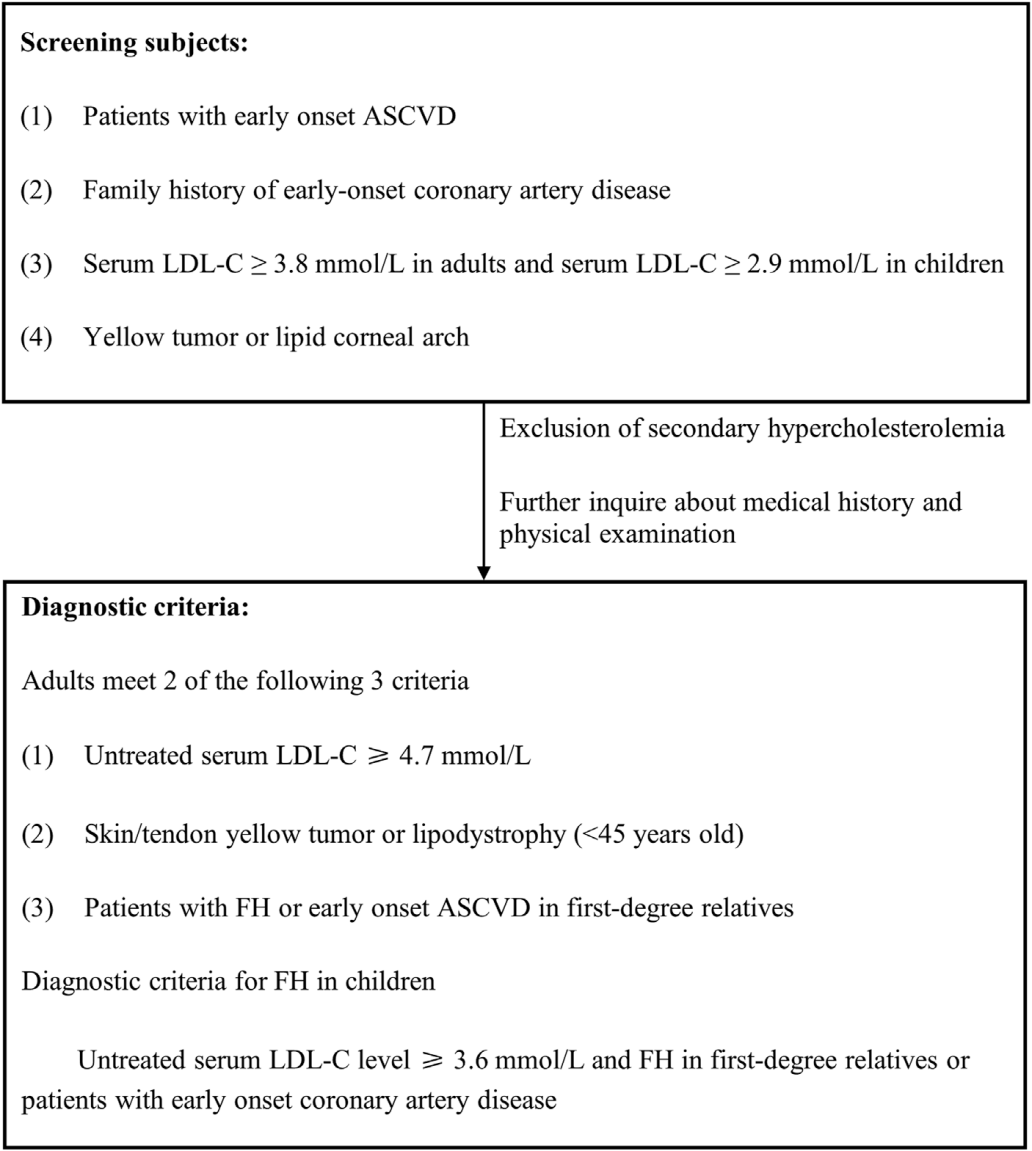
Screening and clinical diagnosis process of FH. ASCVD: atherosclerotic cardiovascular disease; LDL-C: low-density lipoprotein cholesterol; FH: familial hypercholesterolemia. “Early onset” refers to age of diagnosis < 55 years for men and < 65 years for women).

The main clinical features of FH are significantly elevated plasma LDL-C levels, early onset coronary artery disease, and both have familial aggregation. The more commonly used international clinical diagnostic criteria for adult HeFH include the Dutch Lipid Clinical Network criteria and the British Simon Broome criteria. China’s FH screening and diagnosis can be based on the Chinese FH expert consensus criteria (Figure 2) (Atherosclerosis and Coronary Heart Disease Group of the Chinese Society of Cardiology of Chinese Medical Association and Editorial Board of Chinese Journal of Cardiology, 2018) or the simplified Chinese FH criteria derived from the national FH cohort, which have similar sensitivity and specificity to the Simon Broome criteria and the Dutch Lipid Clinical Network criteria (Cao Y.X et al., 2019b). Early detection and diagnosis for early initiation and lifelong adherence to cholesterol-lowering therapy are fundamental therapeutic measures to prevent cardiovascular complications in patients with FH (Supplementary Table S6). It is worth pointing out that genetic diagnosis of FH can be expanded with genetic tests for lysosomal acid lipase, signal transducing adaptor protein 1, ApoE, ABCG5 and ABCG8 in addition to routine LDLR, ApoB, PCSK9 and LDLRAP1 genetic tests, which can help in diagnosis and differential diagnosis (Cao et al., 2018; Cao et al., 2021).

## 13 Joint Committee on the 2023 Chinese guideline for lipid management

### 13.1 Organizations involved in the guideline revision

National Expert Committee for Cardiovascular Diseases, Chinese Society of Cardiology, Chinese Society of Endocrinology, Chinese Diabetes Society, Chinese Society of Laboratory Medicine, Chinese Stroke Association.

### 13.2 Guideline revision steering committee (sorted by last name character in Chinese)

Chengbin Wang (PLA Medical Laboratory Quality Control Center of Chinese PLA General Hospital, Chairman of Chinese Society of Laboratory Medicine), Yongjun Wang (Beijing Tiantan Hospital, Capital Medical University, President of Chinese Stroke Association), Guang Ning (Ruijin Hospital, Shanghai Jiaotong University School of Medicine), Dalong Zhu (Nanjing Drum Tower Hospotal, the Affiliated Hospital of Nanjing University Medical School, Chairman of Chinese Diabetes Society), Yun Zhang (Qilu Hospital of Shandong University), Jiajun Zhao (Shangdong Provincial Hospital, Chairman of Chinese Society of Endocrinology), Dayi Hu (Peking University People’s Hospital), Weiping Jia (Shanghai Sixth People’s Hospital), Dongfeng Gu (National Center for Cardiovascular Diseases, FuWai Hospital, Chinese Academy of Medical Sciences), Junbo Ge (Zhongshan Hospital, Fudan University), Yaling Han (General Hospital of Northern Theater Command of Chinese People’s Liberation Army, Chairman of Chinese Society of Cardiology).

### 13.3 Head of the expert group for the guideline revision (sorted by last name character in Chinese)

#### 13.3.1 Coordinator

Runlin Gao (National Center for Cardiovascular Diseases, FuWai Hospital, Chinese Academy of Medical Sciences).

#### 13.3.2 Member

Jianjun Li (National Center for Cardiovascular Diseases, FuWai Hospital, Chinese Academy of Medical Sciences), Guoping Lu (Ruijin Hospital, Shanghai Jiaotong University School of Medicine), Dong Zhao (Beijing Anzhen Hospital, Capital Medical University), Shuiping Zhao (The Second Xiangya Hospital of Central South University).

#### 13.3.3 Expert group for the guideline revision (sorted by last name character in Chinese)

Bo Yu (The 2nd Affiliated Hospital of Harbin Medical University), Changsheng Ma (Beijing Anzhen Hospital, Capital Medical University), Yitong Ma (First Affiliated Hospital of Xinjiang Medical University), Zengwu Wang (National Center for Cardiovascular Diseases, FuWai Hospital, Chinese Academy of Medical Sciences), Ping Ye (Chinese PLA General Hospital), Xubo Shi (Beijing Tongren Hospital, CMU), Wei Xiang (Hainan Maternal and Child Health Hospital), Meilin Liu (Peking University First Hospital), Yihong Sun (China-Japan Friendship Hospital), Linong Ji (Peking University People’s Hospital), Xiaowei Yan (Peking Union Medical College Hospital), Yong Li (Huashan Hospital, Fudan University), Jing Li (National Center for Cardiovascular Diseases, FuWai Hospital, Chinese Academy of Medical Sciences), Xiaoying Li (Chinese PLA General Hospital), Guangwei Li (Fuwai Hospital Chinese Academy of Medical Sciences), Naqiong Wu (FuWai Hospital Chinese Academy of Medical Sciences), Dajin Zou (Shanghai Changhai Hospital), Jian Zhang (National Institute for Nutrition and Health of Chinese Center for Disease Control and Prevention), Ruiyan Zhang (Ruijin Hospital, Shanghai Jiaotong University School of Medicine), Hong Chen (Peking University People’s Hospital), Zhenyue Chen (Ruijin Hospital, Shanghai Jiaotong University School of Medicine), Yundai Chen (Chinese PLA General Hospital), Yangfeng Wu (School of Public Health of Peking University), Zhou Zhou (FuWai Hospital Chinese Academy of Medical Sciences), Wenhua Zhao (National Institute for Nutrition and Health of Chinese Center for Disease Control and Prevention), Ye Zhu (West China Hospital of Sichuan University), Zhiming Zhu (Daping Hospital, Army Medical University of PLA), Zuyi Yuan (The First Affiliated Hospital of Xi’an Jiaotong University), Wei Gao (Peking University Third Hospital), Yifang Guo (Hebei General Hospital), Yuanlin Guo (FuWai Hospital Chinese Academy of Medical Sciences), Yida Tang (Peking University Third Hospital), Chun Liang (Shanghai Changzheng Hospital), Daoquan Peng (The Second Xiangya Hospital of Central South University), Yugang Dong (The First Affiliated Hospital, Sun Yat-sen University), Xiang Cheng (Union Hospital, Tongji Medical College, Huazhong University of Science and Technology), Zhengpei Zeng (Peking Union Medical College Hospital), Shengkai Yan (Affiliated Hospital of Zunyi Medical University, School of Laboratory Medicine of Zunyi Medical University), Siyan Zhan (School of Public Health of Peking University), Kefei Dou (FuWai Hospital Chinese Academy of Medical Sciences), Yuhua Liao (Union Hospital, Tongji Medical College, Huazhong University of Science and Technology), Yong Huo (Peking University First Hospital).

#### 13.3.4 Experts contributing to writing of the guideline revision (sorted by last name character in Chinese)

Zengwu Wang (National Center for Cardiovascular Diseases, FuWai Hospital Chinese Academy of Medical Sciences), Jing Liu (Beijing Anzhen Hospital, Capital Medical University), Jianjun Li (National Center for Cardiovascular Diseases, FuWai Hospital Chinese Academy of Medical Sciences), Naqiong Wu (FuWai Hospital Chinese Academy of Medical Sciences), Guoping Lu (Ruijin Hospital, Shanghai Jiaotong University School of Medicine), Zhenyue Chen (Ruijin Hospital, Shanghai Jiaotong University School of Medicine), Dong Zhao (Beijing Anzhen Hospital, Capital Medical University), Shuiping Zhao (The Second Xiangya Hospital of Central South University), Runlin Gao (National Center for Cardiovascular Diseases, FuWai Hospital Chinese Academy of Medical Sciences), Yuanlin Guo (FuWai Hospital Chinese Academy of Medical Sciences), Daoquan Peng (The Second Xiangya Hospital of Central South University), Shengkai Yan (Affiliated Hospital of Zunyi Medical University, School of Laboratory Medicine of Zunyi Medical University)

### 13.4 Secretary group for the guideline revision (sorted by last name character in Chinese)

#### 13.4.1 Head of the group

Zengwu Wang (National Center for Cardiovascular Diseases, FuWai Hospital Chinese Academy of Medical Sciences).

#### 13.4.2 Member

Liyuan Ma (National Center for Cardiovascular Diseases, FuWai Hospital Chinese Academy of Medical Sciences), Ying Gao (FuWai Hospital Chinese Academy of Medical Sciences).

## References

[B1] Abifadel M. Varret M. Rabes J. P. Allard D. Ouguerram K. Devillers M. (2003). Mutations in PCSK9 cause autosomal dominant hypercholesterolemia. Nat. Genet. 34 (2), 154–156. 10.1038/ng1161 12730697

[B2] ALLHAT Officers and Coordinators for the ALLHAT Collaborative Research Group, and the Antihypertensive and Lipid-Lowering Treatment to Prevent Heart Attack Trial (2002). Major outcomes in moderately hypercholesterolemic, hypertensive patients randomized to pravastatin vs usual care: the Antihypertensive and Lipid-Lowering Treatment to Prevent Heart Attack Trial (ALLHAT-LLT). JAMA 288 (23), 2998–3007. 10.1001/jama.288.23.2998 12479764

[B3] Amarenco P. Bogousslavsky J. Callahan A. 3rd Goldstein L. B. Hennerici M. Rudolph A. E. (2006). High-dose atorvastatin after stroke or transient ischemic attack. N. Engl. J. Med. 355 (6), 549–559. 10.1056/NEJMoa061894 16899775

[B4] Amarenco P. Labreuche J. (2009). Lipid management in the prevention of stroke: review and updated meta-analysis of statins for stroke prevention. Lancet Neurol. 8 (5), 453–463. 10.1016/S1474-4422(09)70058-4 19375663

[B5] Atherosclerosis and Coronary Heart Disease Group of the Chinese Society of Cardiology of Chinese Medical Association, and Editorial Board of Chinese Journal of CardiologyEditorial Board of Chinese Journal of Cardiology (2018). Chinese expert consensus on screening, diagnosis and treatment of familial hypercholesterolemia (in Chinese). Chin. J. Cardiol. 46 (2), 99–103. 10.3760/cma.j.issn.0253-3758.2018.02.006

[B6] Atherosclerosis and Coronary Heart Disease Working Group of Chinese Society of CardiologyEditorial Board of Chinese Journal of Cardiology (2020). Chinese expert consensus on lipid management of very high-risk atherosclerotic cardiovascular disease patients (in Chinese). Chin. J. Cardiol. 48 (4), 280–286. 10.3760/cma.j.cn112148-20200121-00036 32370478

[B7] Bach R. G. Cannon C. P. Giugliano R. P. White J. A. Lokhnygina Y. Bohula E. A. (2019). Effect of simvastatin-ezetimibe compared with simvastatin monotherapy after acute coronary syndrome among patients 75 Years or older: a secondary analysis of a randomized clinical trial. JAMA Cardiol. 4 (9), 846–854. 10.1001/jamacardio.2019.2306 31314050 PMC6647004

[B8] Cholesterol Treatment Trialists Collaboration Baigent C. Blackwell L. Emberson J. Holland L. E. Reith C. Bhala N. (2010). Efficacy and safety of more intensive lowering of LDL cholesterol: a meta-analysis of data from 170,000 participants in 26 randomised trials. Lancet 376 (9753), 1670–1681. 10.1016/S0140-6736(10)61350-5 21067804 PMC2988224

[B9] Baigent C. Landray M. J. Reith C. Emberson J. Wheeler D. C. Tomson C. (2011). The effects of lowering LDL cholesterol with simvastatin plus ezetimibe in patients with chronic kidney disease (study of heart and renal protection): a randomised placebo-controlled trial. Lancet 377 (9784), 2181–2192. 10.1016/S0140-6736(11)60739-3 21663949 PMC3145073

[B10] Ballantyne C. M. Banach M. Mancini G. B. J. Lepor N. E. Hanselman J. C. Zhao X. (2018). Efficacy and safety of bempedoic acid added to ezetimibe in statin-intolerant patients with hypercholesterolemia: a randomized, placebo-controlled study. Atherosclerosis 277, 195–203. 10.1016/j.atherosclerosis.2018.06.002 29910030

[B11] Bangalore S. Fayyad R. Kastelein J. J. Laskey R. Amarenco P. DeMicco D. A. (2016). 2013 cholesterol guidelines revisited: percent LDL cholesterol reduction or attained LDL cholesterol level or both for prognosis? Am. J. Med. 129 (4), 384–391. 10.1016/j.amjmed.2015.10.024 26551986

[B12] Bays H. Cohen D. E. Chalasani N. Harrison S. A. The National Lipid Association's Statin Safety Task Force The National Lipid Association's Statin Safety Task, F (2014). An assessment by the statin liver safety task force: 2014 update. J. Clin. Lipidol. 8 (3), S47–S57. 10.1016/j.jacl.2014.02.011 24793441

[B13] Beaumont J. L. Carlson L. A. Cooper G. R. Fejfar Z. Fredrickson D. S. Strasser T. (1970). Classification of hyperlipidaemias and hyperlipoproteinaemias. Bull. World Health Organ 43 (6), 891–915. 4930042 PMC2427808

[B14] Beijing Society of Cardiology (2021). Expert statement on the relationship between lipoprotein (a) and cardiovascular disease risk and clinical management (in Chinese). Chin. Circ. J. 36 (12), 1158–1167. 10.3969/j.issn.1000-3614.2021.12.003

[B15] Bhatt D. L. Steg P. G. Miller M. Brinton E. A. Jacobson T. A. Ketchum S. B. (2019). Cardiovascular risk reduction with icosapent ethyl for hypertriglyceridemia. N. Engl. J. Med. 380 (1), 11–22. 10.1056/NEJMoa1812792 30415628

[B16] Blaha M. J. Cainzos-Achirica M. Greenland P. McEvoy J. W. Blankstein R. Budoff M. J. (2016). Role of coronary artery calcium score of zero and other negative risk markers for cardiovascular disease: the multi-ethnic study of atherosclerosis (MESA). Circulation 133 (9), 849–858. 10.1161/CIRCULATIONAHA.115.018524 26801055 PMC4775391

[B17] Boekholdt S. M. Arsenault B. J. Mora S. Pedersen T. R. LaRosa J. C. Nestel P. J. (2012). Association of LDL cholesterol, non-HDL cholesterol, and apolipoprotein B levels with risk of cardiovascular events among patients treated with statins: a meta-analysis. JAMA 307 (12), 1302–1309. 10.1001/jama.2012.366 22453571

[B18] Bohula E. A. Morrow D. A. Giugliano R. P. Blazing M. A. He P. Park J. G. (2017a). Atherothrombotic risk stratification and ezetimibe for secondary prevention. J. Am. Coll. Cardiol. 69 (8), 911–921. 10.1016/j.jacc.2016.11.070 28231942

[B19] Bohula E. A. Wiviott S. D. Giugliano R. P. Blazing M. A. Park J. G. Murphy S. A. (2017b). Prevention of stroke with the addition of ezetimibe to statin therapy in patients with acute coronary syndrome in IMPROVE-IT (improved reduction of outcomes: vytorin efficacy international trial). Circulation 136 (25), 2440–2450. 10.1161/CIRCULATIONAHA.117.029095 28972004

[B20] Boren J. Chapman M. J. Krauss R. M. Packard C. J. Bentzon J. F. Binder C. J. (2020). Low-density lipoproteins cause atherosclerotic cardiovascular disease: pathophysiological, genetic, and therapeutic insights: a consensus statement from the European atherosclerosis society consensus Panel. Eur. Heart J. 41 (24), 2313–2330. 10.1093/eurheartj/ehz962 32052833 PMC7308544

[B21] Byrne P. Demasi M. Jones M. Smith S. M. O'Brien K. K. DuBroff R. (2022). Evaluating the association between low-density lipoprotein cholesterol reduction and relative and absolute effects of statin treatment: a systematic review and meta-analysis. JAMA Intern Med. 182 (5), 474–481. 10.1001/jamainternmed.2022.0134 35285850 PMC8922205

[B22] Cannon C. P. Blazing M. A. Giugliano R. P. McCagg A. White J. A. Theroux P. (2015). Ezetimibe added to statin therapy after acute coronary syndromes. N. Engl. J. Med. 372 (25), 2387–2397. 10.1056/NEJMoa1410489 26039521

[B23] Cannon C. P. Braunwald E. McCabe C. H. Rader D. J. Rouleau J. L. Belder R. (2004). Intensive versus moderate lipid lowering with statins after acute coronary syndromes. N. Engl. J. Med. 350 (15), 1495–1504. 10.1056/NEJMoa040583 15007110

[B24] Cao Y Y. Yan L. Guo N. Yu N. Wang Y. Cao X. (2019). Non-high-density lipoprotein cholesterol and risk of cardiovascular disease in the general population and patients with type 2 diabetes: a systematic review and meta-analysis. Diabetes Res. Clin. Pract. 147, 1–8. 10.1016/j.diabres.2018.11.002 30448450

[B25] Cao Y. X. Liu H. H. Li S. Li J. J. (2019a). A meta-analysis of the effect of PCSK9-monoclonal antibodies on circulating lipoprotein (a) levels. Am. J. Cardiovasc Drugs 19 (1), 87–97. 10.1007/s40256-018-0303-2 30229525

[B26] Cao Y. X. Sun D. Liu H. H. Jin J. L. Li S. Guo Y. L. (2019b). A novel modified system of simplified Chinese criteria for familial hypercholesterolemia (SCCFH). Mol. Diagn Ther. 23 (4), 547–553. 10.1007/s40291-019-00405-1 31172370

[B27] Cao Y. X. Sun D. Liu H. H. Jin J. L. Li S. Guo Y. L. (2021). Improvement of definite diagnosis of familial hypercholesterolemia using an expanding genetic analysis. JACC Asia 1 (1), 82–89. 10.1016/j.jacasi.2021.04.001 36338372 PMC9627923

[B28] Cao Y. X. Wu N. Q. Sun D. Liu H. H. Jin J. L. Li S. (2018). Application of expanded genetic analysis in the diagnosis of familial hypercholesterolemia in patients with very early-onset coronary artery disease. J. Transl. Med. 16 (1), 345. 10.1186/s12967-018-1737-7 30526649 PMC6288904

[B29] Cegla J. Neely R. D. G. France M. Ferns G. Byrne C. D. Halcox J. (2019). HEART UK consensus statement on lipoprotein(a): a call to action. Atherosclerosis 291, 62–70. 10.1016/j.atherosclerosis.2019.10.011 31704552

[B30] Center for Disease Control and Prevention of National Health Commission of the People's Republic of China (2015). Report on nutrition and chronic diseases of Chinese residents 2015 (in Chinese). Beijing: People's Medical Press.

[B31] Center for Disease Control and Prevention of National Health Commission of the People's Republic of China (2020). Report on nutrition and chronic diseases of Chinese residents 2020 (in Chinese). Beijing: People's Medical Press.

[B32] Chait A. Ginsberg H. N. Vaisar T. Heinecke J. W. Goldberg I. J. Bornfeldt K. E. (2020). Remnants of the triglyceride-rich lipoproteins, diabetes, and cardiovascular disease. Diabetes 69 (4), 508–516. 10.2337/dbi19-0007 32198194 PMC7085249

[B33] Chapman M. J. Ginsberg H. N. Amarenco P. Andreotti F. Boren J. Catapano A. L. (2011). Triglyceride-rich lipoproteins and high-density lipoprotein cholesterol in patients at high risk of cardiovascular disease: evidence and guidance for management. Eur. Heart J. 32 (11), 1345–1361. 10.1093/eurheartj/ehr112 21531743 PMC3105250

[B34] Chen H. Ren J. Wu B. Liu X. Wang R. Li L. (2007). The effects after withdrawal of simvastatin on brachial artery endothelial function in patient with coronary heart disease or risk factors (in Chinese). Chin. J. Cardiol. 35 (6), 531–535. 10.3760/j.issn:0253-3758.2007.06.009 17711713

[B35] Chen J. Lou H. Jiang B. Shao R. Ruan Z. Wang J. (2015). Simultaneous determination of hyzetimibe and its main active metabolite in plasma by LC-MS/MS and its application in PK study. Bioanalysis 7 (15), 1857–1867. 10.4155/bio.15.114 26295987

[B36] Chen Y. Yuan Z. Lu J. Eliaschewitz F. G. Lorenzatti A. J. Monsalvo M. L. (2019). Randomized study of evolocumab in patients with type 2 diabetes and dyslipidaemia on background statin: pre-specified analysis of the Chinese population from the BERSON clinical trial. Diabetes Obes. Metab. 21 (6), 1464–1473. 10.1111/dom.13700 30851062 PMC6594089

[B37] China Hypertension Survey and Research Group (2019). Status of dyslipidemia among adults aged 35 Years and above in China (in Chinese). Chin. Circ. J. 34, 681–687. 10.3969/jssn.1000-3614.2019.07.011

[B38] Chinese Cardiovascular Journal Editorial Board Dyslipidemia Prevention and Treatment Countermeasures Special Group (1997). Recommendations for prevention and treatment of dyslipidemia (in Chinese). Chin. J. Cardiol. 25 (3), 169–172.

[B39] Chinese Society of Cardiology of Chinese Medical AssociationCardiovascular Disease Prevention and Rehabilitation Committee of Chinese Association of Rehabilitation MedicineCardiovascular Disease Committee of Chinese Association of Gerontology and GeriatricsThrombosis Prevention and Treatment Committee of Chinese Medical Doctor Association (2020). Chinese guideline on the primary prevention of cardiovascular diseases (in Chinese). Chin. J. Cardiol. 48 (12), 1000–1038. 10.3760/cma.j.cn112148-20201009-00796

[B40] Cholesterol Treatment Trialists Collaboration (2019). Efficacy and safety of statin therapy in older people: a meta-analysis of individual participant data from 28 randomised controlled trials. Lancet 393 (10170), 407–415. 10.1016/S0140-6736(18)31942-1 30712900 PMC6429627

[B41] Christian J. B. Arondekar B. Buysman E. K. Johnson S. L. Seeger J. D. Jacobson T. A. (2012). Clinical and economic benefits observed when follow-up triglyceride levels are less than 500 mg/dL in patients with severe hypertriglyceridemia. J. Clin. Lipidol. 6 (5), 450–461. 10.1016/j.jacl.2012.08.007 23009781

[B42] Chu J. Ye P. Kou W. Qi W. Ma H. (2002). Efficacy and tolerability study of micronized fenofibrate in the treatment of dyslipidemia (in Chinese). Chin. J. Cardiol. 30 (3), 27–30. 10.3760/j:issn:0253-3758.2002.03.007

[B43] Cicero A. F. Tartagni E. Ertek S. (2014). Safety and tolerability of injectable lipid-lowering drugs: a review of available clinical data. Expert Opin. Drug Saf. 13 (8), 1023–1030. 10.1517/14740338.2014.932348 24961142

[B44] Clifton P. M. Keogh J. B. (2017). A systematic review of the effect of dietary saturated and polyunsaturated fat on heart disease. Nutr. Metab. Cardiovasc Dis. 27 (12), 1060–1080. 10.1016/j.numecd.2017.10.010 29174025

[B45] Colhoun H. M. Betteridge D. J. Durrington P. N. Hitman G. A. Neil H. A. Livingstone S. J. (2004). Primary prevention of cardiovascular disease with atorvastatin in type 2 diabetes in the collaborative atorvastatin diabetes study (CARDS): multicentre randomised placebo-controlled trial. Lancet 364 (9435), 685–696. 10.1016/S0140-6736(04)16895-5 15325833

[B46] Collaborative Group on Xuezhikang for Secondary Prevention of Coronary Artery Disease (2005). China coronary secondary prevention study (CCSPS) (in Chinese). Chin. J. Cardiol. 33 (2), 109–115. 10.3760/j:issn:0253-3758.2005.02.003

[B47] Collins R. Armitage J. Parish S. Sleigh P. Peto R. Heart Protection Study Collaborative G. (2003). MRC/BHF heart protection study of cholesterol-lowering with simvastatin in 5963 people with diabetes: a randomised placebo-controlled trial. Lancet 361 (9374), 2005–2016. 10.1016/s0140-6736(03)13636-7 12814710

[B48] Costantine M. M. West H. Wisner K. L. Caritis S. Clark S. Venkataramanan R. (2021). A randomized pilot clinical trial of pravastatin versus placebo in pregnant patients at high risk of preeclampsia. Am. J. Obstet. Gynecol. 225 (6), 666.e1–666666.e15. 10.1016/j.ajog.2021.05.018 PMC8611118 34033812

[B49] Cuchel M. Bruckert E. Ginsberg H. N. Raal F. J. Santos R. D. Hegele R. A. (2014). Homozygous familial hypercholesterolaemia: new insights and guidance for clinicians to improve detection and clinical management. A position paper from the consensus Panel on familial hypercholesterolaemia of the European atherosclerosis society. Eur. Heart J. 35 (32), 2146–2157. 10.1093/eurheartj/ehu274 25053660 PMC4139706

[B50] Cuchel M. Meagher E. A. du Toit Theron H. Blom D. J. Marais A. D. Hegele R. A. (2013). Efficacy and safety of a microsomal triglyceride transfer protein inhibitor in patients with homozygous familial hypercholesterolaemia: a single-arm, open-label, phase 3 study. Lancet 381 (9860), 40–46. 10.1016/S0140-6736(12)61731-0 23122768 PMC4587657

[B51] Das Pradhan A. Glynn R. J. Fruchart J. C. MacFadyen J. G. Zaharris E. S. Everett B. M. (2022). Triglyceride lowering with pemafibrate to reduce cardiovascular risk. N. Engl. J. Med. 387 (21), 1923–1934. 10.1056/NEJMoa2210645 36342113

[B52] de Lemos J. A. Blazing M. A. Wiviott S. D. Lewis E. F. Fox K. A. White H. D. (2004). Early intensive vs a delayed conservative simvastatin strategy in patients with acute coronary syndromes: phase Z of the A to Z trial. JAMA 292 (11), 1307–1316. 10.1001/jama.292.11.1307 15337732

[B53] Emerging Risk Factors Collaboration Di Angelantonio E. Gao P. Pennells L. Kaptoge S. Caslake M. Thompson A. (2012). Lipid-related markers and cardiovascular disease prediction. JAMA 307 (23), 2499–2506. 10.1001/jama.2012.6571 22797450 PMC4211641

[B54] Di Taranto M. D. Giacobbe C. Palma D. Iannuzzo G. Gentile M. Calcaterra I. (2021). Genetic spectrum of familial hypercholesterolemia and correlations with clinical expression: implications for diagnosis improvement. Clin. Genet. 100 (5), 529–541. 10.1111/cge.14036 34297352 PMC9291778

[B55] Diaz R. Li Q. H. Bhatt D. L. Bittner V. A. Baccara-Dinet M. T. Goodman S. G. (2021). Intensity of statin treatment after acute coronary syndrome, residual risk, and its modification by alirocumab: insights from the ODYSSEY OUTCOMES trial. Eur. J. Prev. Cardiol. 28 (1), 33–43. 10.1177/2047487320941987 33755145

[B56] Ding W. Cheng H. Yan Y. Zhao X. Chen F. Huang G. (2016). 10-Year trends in serum lipid levels and dyslipidemia among children and adolescents from several schools in beijing, China. J. Epidemiol. 26 (12), 637–645. 10.2188/jea.JE20140252 27397598 PMC5121432

[B57] Ding W. Dong H. Mi J. (2015). Prevalence of dyslipidemia in Chinese children and adolescents: a Meta-analysis. Chin. J. Epidemiol. 36 (1), 71–77. 10.3760/cma.j.issn.0254-6450.2015.01.017 25876870

[B58] Djousse L. Gaziano J. M. Buring J. E. Lee I. M. (2009). Egg consumption and risk of type 2 diabetes in men and women. Diabetes Care 32 (2), 295–300. 10.2337/dc08-1271 19017774 PMC2628696

[B59] Dobert M. Varouxaki A. N. Mu A. C. Syngelaki A. Ciobanu A. Akolekar R. (2021). Pravastatin versus placebo in pregnancies at high risk of term preeclampsia. Circulation 144 (9), 670–679. 10.1161/CIRCULATIONAHA.121.053963 34162218

[B60] Downs J. R. Clearfield M. Weis S. Whitney E. Shapiro D. R. Beere P. A. (1998). Primary prevention of acute coronary events with lovastatin in men and women with average cholesterol levels: results of AFCAPS/TexCAPS. Air force/Texas coronary atherosclerosis prevention study. JAMA 279 (20), 1615–1622. 10.1001/jama.279.20.1615 9613910

[B61] Echocardiography Group of Chinese Society of Ultrasound in Medicine (2016). Echocardiography measurement guidelines for Chinese adults (in Chinese). Chin. J. Ultrason. 25 (8), 645–666. 10.3760/cma.j.issn.1004-4477.2016.08.001

[B62] Elam M. B. Ginsberg H. N. Lovato L. C. Corson M. Largay J. Leiter L. A. (2017). Association of fenofibrate therapy with long-term cardiovascular risk in statin-treated patients with type 2 diabetes. JAMA Cardiol. 2 (4), 370–380. 10.1001/jamacardio.2016.4828 28030716 PMC5470410

[B63] Elkins C. Fruh S. Jones L. Bydalek K. (2019). Clinical practice recommendations for pediatric dyslipidemia. J. Pediatr. Health Care 33 (4), 494–504. 10.1016/j.pedhc.2019.02.009 31227123

[B64] Emerging Risk Factors Collaboration Erqou S. Kaptoge S. Perry P. L. Di Angelantonio E. Thompson A. White I. R. (2009). Lipoprotein(a) concentration and the risk of coronary heart disease, stroke, and nonvascular mortality. JAMA 302 (4), 412–423. 10.1001/jama.2009.1063 19622820 PMC3272390

[B65] Expert Dyslipidemia Panel (2013). An international atherosclerosis society position paper: global recommendations for the management of dyslipidemia. J. Clin. Lipidol. 7 (6), 561–565. 10.1016/j.jacl.2013.10.001 24314355

[B66] Fellstrom B. C. Jardine A. G. Schmieder R. E. Holdaas H. Bannister K. Beutler J. (2009). Rosuvastatin and cardiovascular events in patients undergoing hemodialysis. N. Engl. J. Med. 360 (14), 1395–1407. 10.1056/NEJMoa0810177 19332456

[B67] Ference B. A. Ginsberg H. N. Graham I. Ray K. K. Packard C. J. Bruckert E. (2017). Low-density lipoproteins cause atherosclerotic cardiovascular disease. 1. Evidence from genetic, epidemiologic, and clinical studies. A consensus statement from the European Atherosclerosis Society Consensus Panel. Eur. Heart J. 38 (32), 2459–2472. 10.1093/eurheartj/ehx144 28444290 PMC5837225

[B68] Ference B. A. Kastelein J. J. P. Ray K. K. Ginsberg H. N. Chapman M. J. Packard C. J. (2019). Association of triglyceride-lowering LPL variants and LDL-C-lowering LDLR variants with risk of coronary heart disease. JAMA 321 (4), 364–373. 10.1001/jama.2018.20045 30694319 PMC6439767

[B69] Fitzgerald K. White S. Borodovsky A. Bettencourt B. R. Strahs A. Clausen V. (2017). A highly durable RNAi therapeutic inhibitor of PCSK9. N. Engl. J. Med. 376 (1), 41–51. 10.1056/NEJMoa1609243 27959715 PMC5778873

[B70] Ford E. S. Ajani U. A. Croft J. B. Critchley J. A. Labarthe D. R. Kottke T. E. (2007). Explaining the decrease in U.S. deaths from coronary disease, 1980-2000. N. Engl. J. Med. 356 (23), 2388–2398. 10.1056/NEJMsa053935 17554120

[B71] Fruchart J. C. (2017). Pemafibrate (K-877), a novel selective peroxisome proliferator-activated receptor alpha modulator for management of atherogenic dyslipidaemia. Cardiovasc Diabetol. 16 (1), 124. 10.1186/s12933-017-0602-y 28978316 PMC5628452

[B72] Cholesterol Treatment Trialists Collaboration Fulcher J. O'Connell R. Voysey M. Emberson J. Blackwell L. Mihaylova B. (2015). Efficacy and safety of LDL-lowering therapy among men and women: meta-analysis of individual data from 174,000 participants in 27 randomised trials. Lancet 385 (9976), 1397–1405. 10.1016/S0140-6736(14)61368-4 25579834

[B73] FuWai Hospital (2010). Prediction for ASCVD risk. Online. Available at: https://www.cvdrisk.com.cn/ASCVD/Eval Accessed.

[B74] Gaudet D. Kereiakes D. J. McKenney J. M. Roth E. M. Hanotin C. Gipe D. (2014). Effect of alirocumab, a monoclonal proprotein convertase subtilisin/kexin 9 antibody, on lipoprotein(a) concentrations (a pooled analysis of 150 mg every two weeks dosing from phase 2 trials). Am. J. Cardiol. 114 (5), 711–715. 10.1016/j.amjcard.2014.05.060 25060413

[B75] Geng Q. Ren J. Song J. Li S. Chen H. (2014). Meta-analysis of the effect of statins on renal function. Am. J. Cardiol. 114 (4), 562–570. 10.1016/j.amjcard.2014.05.033 25001155

[B76] Accord Study Group Ginsberg H. N. Elam M. B. Lovato L. C. Crouse J. R. 3rd Leiter L. A. Linz P. (2010). Effects of combination lipid therapy in type 2 diabetes mellitus. N. Engl. J. Med. 362 (17), 1563–1574. 10.1056/NEJMoa1001282 20228404 PMC2879499

[B77] Ginsberg H. N. Karmally W. Siddiqui M. Holleran S. Tall A. R. Blaner W. S. (1995). Increases in dietary cholesterol are associated with modest increases in both LDL and HDL cholesterol in healthy young women. Arterioscler. Thromb. Vasc. Biol. 15 (2), 169–178. 10.1161/01.atv.15.2.169 7749822 PMC3287065

[B78] Ginsberg H. N. Karmally W. Siddiqui M. Holleran S. Tall A. R. Rumsey S. C. (1994). A dose-response study of the effects of dietary cholesterol on fasting and postprandial lipid and lipoprotein metabolism in healthy young men. Arterioscler. Thromb. 14 (4), 576–586. 10.1161/01.atv.14.4.576 8148356 PMC3292202

[B79] Giugliano R. P. Mach F. Zavitz K. Kurtz C. Im K. Kanevsky E. (2017). Cognitive function in a randomized trial of evolocumab. N. Engl. J. Med. 377 (7), 633–643. 10.1056/NEJMoa1701131 28813214

[B80] Goldberg A. C. Leiter L. A. Stroes E. S. G. Baum S. J. Hanselman J. C. Bloedon L. T. (2019). Effect of bempedoic acid vs placebo added to maximally tolerated statins on low-density lipoprotein cholesterol in patients at high risk for cardiovascular disease: the CLEAR wisdom randomized clinical trial. JAMA 322 (18), 1780–1788. 10.1001/jama.2019.16585 31714986 PMC6865290

[B81] Gotto A. M. Jr. Brinton E. A. (2004). Assessing low levels of high-density lipoprotein cholesterol as a risk factor in coronary heart disease: a working group report and update. J. Am. Coll. Cardiol. 43 (5), 717–724. 10.1016/j.jacc.2003.08.061 14998606

[B82] Grundy S. M. Stone N. J. Bailey A. L. Beam C. Birtcher K. K. Blumenthal R. S. (2019). 2018 AHA/ACC/AACVPR/AAPA/ABC/ACPM/ADA/AGS/APhA/ASPC/NLA/PCNA guideline on the management of blood cholesterol: a report of the American College of Cardiology/American heart association task force on clinical practice guidelines. Circulation 139 (25), e1082–e1143. 10.1161/CIR.0000000000000625 30586774 PMC7403606

[B83] Guo L. Zhao S. Zhao W. (2021). The clinical effect of Xuezhikang combined with ezetimibe in the treatment of coronary heart disease in lipid-lowering treatment and its influence on blood lipid level. Panminerva Med. 2021, 4624–4633. 10.23736/S0031-0808.21.04624-3 34931509

[B84] Hackam D. G. Woodward M. Newby L. K. Bhatt D. L. Shao M. Smith E. E. (2011). Statins and intracerebral hemorrhage: collaborative systematic review and meta-analysis. Circulation 124 (20), 2233–2242. 10.1161/CIRCULATIONAHA.111.055269 22007076

[B85] Han Y. Chen J. Chopra V. K. Zhang S. Su G. Ma C. (2020). Odyssey EAST: alirocumab efficacy and safety vs ezetimibe in high cardiovascular risk patients with hypercholesterolemia and on maximally tolerated statin in China, India, and Thailand. J. Clin. Lipidol. 14 (1), 98–108. e108. 10.1016/j.jacl.2019.10.015 31882376

[B86] Han Y. Ma Y. Su G. Li Y. Li Y. Liu D. (2022). Efficacy and safety of alirocumab versus ezetimibe in high cardiovascular risk Chinese patients with hyperlipidemia: odyssey east study-Chinese sub-population analysis. Chin. J. Cardiol. 48 (7), 593–599. 10.3760/cma.j.cn112148-20191216-00755 32842270

[B87] Harada-Shiba M. Arai H. Ishigaki Y. Ishibashi S. Okamura T. Ogura M. (2018a). Guidelines for diagnosis and treatment of familial hypercholesterolemia 2017. J. Atheroscler. Thromb. 25 (8), 751–770. 10.5551/jat.CR003 29877295 PMC6099072

[B88] Harada-Shiba M. Ohta T. Ohtake A. Ogura M. Dobashi K. Nohara A. (2018b). Guidance for pediatric familial hypercholesterolemia 2017. J. Atheroscler. Thromb. 25 (6), 539–553. 10.5551/jat.CR002 29415907 PMC6005224

[B89] Harris W. S. (1996). n-3 fatty acids and lipoproteins: comparison of results from human and animal studies. Lipids 31 (3), 243–252. 10.1007/BF02529870 8900453

[B90] Harris W. S. (1997). n-3 fatty acids and serum lipoproteins: human studies. Am. J. Clin. Nutr. 65 (5), 1645S–1654S. 10.1093/ajcn/65.5.1645S 9129504

[B91] He Z. Yan S. (2021). LDL-C measurement: status and development (in Chinese). Exp Lab Med 39 (6), 1327–1332. 10.3969/j.issn.1674-1129.2021.06.001

[B92] Healthy China Initiative Promotion Committee (2019). Healthy China initiative (2019-2030). [Online]. Available at: http://www.gov.cn/xinwen/2019-07/15/content_5409694.htm Accessed.

[B93] Heart Protection Study Collaborative Group (2002). MRC/BHF heart protection study of cholesterol lowering with simvastatin in 20,536 high-risk individuals: a randomised placebo-controlled trial. Lancet 360 (9326), 7–22. 10.1016/S0140-6736(02)09327-3 12114036

[B94] Hegele R. A. Ginsberg H. N. Chapman M. J. Nordestgaard B. G. Kuivenhoven J. A. Averna M. (2014). The polygenic nature of hypertriglyceridaemia: implications for definition, diagnosis, and management. Lancet Diabetes Endocrinol. 2 (8), 655–666. 10.1016/S2213-8587(13)70191-8 24731657 PMC4201123

[B95] Heigl F. Hettich R. Lotz N. Reeg H. Pflederer T. Osterkorn D. (2015). Efficacy, safety, and tolerability of long-term lipoprotein apheresis in patients with LDL- or Lp(a) hyperlipoproteinemia: findings gathered from more than 36,000 treatments at one center in Germany. Atheroscler. Suppl. 18, 154–162. 10.1016/j.atherosclerosissup.2015.02.013 25936320

[B96] Cholesterol Treatment Trialists Collaboration Herrington W. G. Emberson J. Mihaylova B. Blackwell L. Reith C. Solbu M. D. (2016). Impact of renal function on the effects of LDL cholesterol lowering with statin-based regimens: a meta-analysis of individual participant data from 28 randomised trials. Lancet Diabetes Endocrinol. 4 (10), 829–839. 10.1016/S2213-8587(16)30156-5 27477773

[B97] Hu D. (2008). Expert consensus on the clinical application of polyhexanol: a new lipid-regulating phytopharmaceutical (in Chinese). Chin. J. Intern Med. 47 (11), 961–963. 10.3321/j.issn:0578-1426.2008.11.032

[B98] Hu Y. Hu F. B. Manson J. E. (2019). Marine omega-3 supplementation and cardiovascular disease: an updated meta-analysis of 13 randomized controlled trials involving 127 477 participants. J. Am. Heart Assoc. 8 (19), e013543. 10.1161/JAHA.119.013543 31567003 PMC6806028

[B99] Hypertensive Group of Chinese Society of Cardiology of Chinese Medical Association, and Editorial Board of Chinese Journal of CardiologyEditorial Board of Chinese Journal of Cardiology (2021). Expert consensus on the comprehensive management of blood pressure and dyslipidemia in Chinese hypertensive patients (in Chinese). Chin. J. Cardiol. 49 (6), 554–563. 10.3760/cma.j.cn112148-20210202-00128 34126722

[B100] Ishigaki Y. Kawagishi N. Hasegawa Y. Sawada S. Katagiri H. Satomi S. (2019). Liver transplantation for homozygous familial hypercholesterolemia. J. Atheroscler. Thromb. 26 (2), 121–127. 10.5551/jat.RV17029 30555131 PMC6365147

[B101] Jacobson T. A. Ito M. K. Maki K. C. Orringer C. E. Bays H. E. Jones P. H. (2015a). National lipid association recommendations for patient-centered management of dyslipidemia: part 1-full report. J. Clin. Lipidol. 9 (2), 129–169. 10.1016/j.jacl.2015.02.003 25911072

[B102] Jacobson T. A. Maki K. C. Orringer C. E. Jones P. H. Kris-Etherton P. Sikand G. (2015b). National lipid association recommendations for patient-centered management of dyslipidemia: part 2. J. Clin. Lipidol. 9 (6), S1–S122.e1. 10.1016/j.jacl.2015.09.002 26699442

[B103] Jiang Z. Zhan S. Jia X. Fang H. Zuo L. Gao R. (2016). Basic methods and procedures for the development/revision of clinical guidelines (in Chinese). Natl. Med. J. China 96 (4), 250–253. 10.3760/cma.j.issn.0376-2491.2016.04.004

[B104] Joint Committee on Chinese Guideline for the Management of Dyslipidemia in Adults (2016). 2016 Chinese guideline for the management of dyslipidemia in adults (in Chinese). Chin. Circ. J. 31 (10), 937–953. 10.3969/j.issn.1000-3614.2016.10.001

[B105] Joint Committee on Chinese Guidelines on Prevention and Treatment of Dyslipidemia in Adults (2007). Chinese guidelines on prevention and treatment of dyslipidemia in adults (in Chinese). Chin. J. Cardiol. 35 (5), 390–419. 10.3760/j.issn:0253-3758.2007.05.003 17711682

[B106] Jun M. Foote C. Lv J. Neal B. Patel A. Nicholls S. J. (2010). Effects of fibrates on cardiovascular outcomes: a systematic review and meta-analysis. Lancet 375 (9729), 1875–1884. 10.1016/S0140-6736(10)60656-3 20462635

[B107] Kaasenbrood L. Boekholdt S. M. van der Graaf Y. Ray K. K. Peters R. J. Kastelein J. J. (2016). Distribution of estimated 10-year risk of recurrent vascular events and residual risk in a secondary prevention population. Circulation 134 (19), 1419–1429. 10.1161/CIRCULATIONAHA.116.021314 27682883

[B108] Cholesterol Treatment Trialists Collaborators Kearney P. M. Blackwell L. Collins R. Keech A. Simes J. Peto R. (2008). Efficacy of cholesterol-lowering therapy in 18,686 people with diabetes in 14 randomised trials of statins: a meta-analysis. Lancet 371 (9607), 117–125. 10.1016/S0140-6736(08)60104-X 18191683

[B109] Keech A. Simes R. J. Barter P. Best J. Scott R. Taskinen M. R. (2005). Effects of long-term fenofibrate therapy on cardiovascular events in 9795 people with type 2 diabetes mellitus (the FIELD study): randomised controlled trial. Lancet 366 (9500), 1849–1861. 10.1016/S0140-6736(05)67667-2 16310551

[B110] Kelley D. S. Adkins Y. (2012). Similarities and differences between the effects of EPA and DHA on markers of atherosclerosis in human subjects. Proc. Nutr. Soc. 71 (2), 322–331. 10.1017/S0029665112000080 22369859

[B111] Khan S. U. Lone A. N. Khan M. S. Virani S. S. Blumenthal R. S. Nasir K. (2021). Effect of omega-3 fatty acids on cardiovascular outcomes: a systematic review and meta-analysis. EClinicalMedicine 38, 100997. 10.1016/j.eclinm.2021.100997 34505026 PMC8413259

[B112] Kim B. K. Hong S. J. Lee Y. J. Hong S. J. Yun K. H. Hong B. K. (2022). Long-term efficacy and safety of moderate-intensity statin with ezetimibe combination therapy versus high-intensity statin monotherapy in patients with atherosclerotic cardiovascular disease (RACING): a randomised, open-label, non-inferiority trial. Lancet 400 (10349), 380–390. 10.1016/S0140-6736(22)00916-3 35863366

[B113] Knapp H. H. Schrott H. Ma P. Knopp R. Chin B. Gaziano J. M. (2001). Efficacy and safety of combination simvastatin and colesevelam in patients with primary hypercholesterolemia. Am. J. Med. 110 (5), 352–360. 10.1016/s0002-9343(01)00638-6 11286949

[B114] Laboratory Medicine Society of Chinese Medical AssociationBranch of Laboratory Physicians of Chinese Medical Doctor AssociationLipids and Lipoproteins CommitteeChinese Society of Biochemistry and Molecular Biology, National Health Commission Clinical Laboratory CenterEditorial Board of Chinese Journal of Laboratory Medicine (2022). China guideline for clinical lipid profile testing (in Chinese). Chin. J. Lab. Med. 45 (10), 1017–1033. 10.3760/cma.j.cn114452-20220829-00497

[B115] Hps2-Thrive Collaborative Group Landray M. J. Haynes R. Hopewell J. C. Parish S. Aung T. Tomson J. (2014). Effects of extended-release niacin with laropiprant in high-risk patients. N. Engl. J. Med. 371 (3), 203–212. 10.1056/NEJMoa1300955 25014686

[B116] LaRosa J. C. Grundy S. M. Waters D. D. Shear C. Barter P. Fruchart J. C. (2005). Intensive lipid lowering with atorvastatin in patients with stable coronary disease. N. Engl. J. Med. 352 (14), 1425–1435. 10.1056/NEJMoa050461 15755765

[B117] Lavigne P. M. Karas R. H. (2013). The current state of niacin in cardiovascular disease prevention: a systematic review and meta-regression. J. Am. Coll. Cardiol. 61 (4), 440–446. 10.1016/j.jacc.2012.10.030 23265337

[B118] Leaf A. Weber P. C. (1988). Cardiovascular effects of n-3 fatty acids. N. Engl. J. Med. 318 (9), 549–557. 10.1056/NEJM198803033180905 3277056

[B119] Lee S. Akioyamen L. E. Aljenedil S. Riviere J. B. Ruel I. Genest J. (2019). Genetic testing for familial hypercholesterolemia: impact on diagnosis, treatment and cardiovascular risk. Eur. J. Prev. Cardiol. 26 (12), 1262–1270. 10.1177/2047487319829746 30755017

[B120] Li J. J. Liu H. H. Wu N. Q. Yeo K. K. Tan K. Ako J. (2020). Statin intolerance: an updated, narrative review mainly focusing on muscle adverse effects. Expert Opin. Drug Metab. Toxicol. 16 (9), 837–851. 10.1080/17425255.2020.1802426 32729743

[B121] Li J. J. Lu Z. L. Kou W. R. Chen Z. Wu Y. F. Yu X. H. (2010). Impact of Xuezhikang on coronary events in hypertensive patients with previous myocardial infarction from the China Coronary Secondary Prevention Study (CCSPS). Ann. Med. 42 (3), 231–240. 10.3109/07853891003652534 20350253

[B122] Li J. J. Ma C. S. Zhao D. Yan X. W. Beijing Heart S. Expert C. (2022). Lipoprotein(a) and cardiovascular disease in Chinese population: a beijing heart society expert scientific statement. JACC Asia 2 (6), 653–665. 10.1016/j.jacasi.2022.08.015 36444328 PMC9700018

[B123] Li J. J. Yang P. Liu J. Jia Y. J. Li Z. C. Guo Y. L. (2012). Impact of 10 mg rosuvastatin daily or alternate-day on lipid profile and inflammatory markers. Clin. Chim. Acta 413 (1-2), 139–142. 10.1016/j.cca.2011.09.006 21983163

[B124] Li M. Li Y. Chen M. Lin H. Wu Y. Zhang Z. (2020). Meta-analysis of Zhibitai Capsules combined with statin in reducing blood lipid levels in patients with coronary heart disease. Chin. J. Chin. Mater. Medica 45 (12), 2966–2974. 10.19540/j.cnki.cjcmm.20200211.502 32627474

[B125] Li S. Liu H. H. Guo Y. L. Zhu C. G. Wu N. Q. Xu R. X. (2021). Improvement of evaluation in Chinese patients with atherosclerotic cardiovascular disease using the very-high-risk refinement: a population-based study. Lancet Reg. Health West Pac 17, 100286. 10.1016/j.lanwpc.2021.100286 34734202 PMC8551815

[B126] Liao J. Wang X. Li Z. Ouyang D. (2021). Pharmacokinetic study of oral (14)C-radiolabeled hyzetimibe, A new cholesterol absorption inhibitor. Front. Pharmacol. 12, 665372. 10.3389/fphar.2021.665372 34122085 PMC8194275

[B127] Liu H. H. Zhang M. Chen R. Z. Zhou J. Y. Qian J. Dou K. F. (2022). Low-density lipoprotein cholesterol in oldest old with acute myocardial infarction: is lower the better? Age Ageing 51 (9), afac202. 10.1093/ageing/afac202 36088600

[B128] Liu J. Hong Y. D'Agostino R. B. Sr. Wu Z. Wang W. (2004). Predictive value for the Chinese population of the Framingham CHD risk assessment tool compared with the Chinese Multi-Provincial Cohort Study. JAMA 291 (21), 2591–2599. 10.1001/jama.291.21.2591 15173150

[B129] Liu L. Zhao S. (2021). Expert consensus on the non-fasting blood lipid detection and clinical application (in Chinese). Chin. J. Intern Med. 60 (5), 400–405. 10.3760/cma.j.cn112138-20200429-00436 33906270

[B130] Liu S. Tan M. Zhao S. Rong H. (2012). Effects of policosanol on serum lipids and heme oxygenase-1 in patients with hyperlipidemia (in Chinese). Chin. J. Cardiol. 40 (10), 840–843. 10.3760/cma.j.issn.0253-3758.2012.10.008 23302671

[B131] Long-Term Intervention with Pravastatin in Ischaemic Disease Study Group (1998). Prevention of cardiovascular events and death with pravastatin in patients with coronary heart disease and a broad range of initial cholesterol levels. N. Engl. J. Med. 339 (19), 1349–1357. 10.1056/NEJM199811053391902 9841303

[B132] Lorenz M. W. Markus H. S. Bots M. L. Rosvall M. Sitzer M. (2007). Prediction of clinical cardiovascular events with carotid intima-media thickness: a systematic review and meta-analysis. Circulation 115 (4), 459–467. 10.1161/CIRCULATIONAHA.106.628875 17242284

[B133] Lu Z. Kou W. Du B. Wu Y. Zhao S. Brusco O. A. (2008). Effect of Xuezhikang, an extract from red yeast Chinese rice, on coronary events in a Chinese population with previous myocardial infarction. Am. J. Cardiol. 101 (12), 1689–1693. 10.1016/j.amjcard.2008.02.056 18549841

[B134] Luirink I. K. Wiegman A. Kusters D. M. Hof M. H. Groothoff J. W. de Groot E. (2019). 20-Year follow-up of statins in children with familial hypercholesterolemia. N. Engl. J. Med. 381 (16), 1547–1556. 10.1056/NEJMoa1816454 31618540

[B135] Mach F. Baigent C. Catapano A. L. Koskinas K. C. Casula M. Badimon L. (2020). 2019 ESC/EAS guidelines for the management of dyslipidaemias: lipid modification to reduce cardiovascular risk. Eur. Heart J. 41 (1), 111–188. 10.1093/eurheartj/ehz455 31504418

[B136] Madsen C. M. Varbo A. Nordestgaard B. G. (2018). Unmet need for primary prevention in individuals with hypertriglyceridaemia not eligible for statin therapy according to European society of Cardiology/European atherosclerosis society guidelines: a contemporary population-based study. Eur. Heart J. 39 (7), 610–619. 10.1093/eurheartj/ehx659 29182745

[B137] Maki K. C. Ridker P. M. Brown W. V. Grundy S. M. Sattar N. The Diabetes Subpanel of the National Lipid Association Expert Panel The Diabetes Subpanel of the National Lipid Association Expert, P (2014). An assessment by the statin diabetes safety task force: 2014 update. J. Clin. Lipidol. 8 (3), S17–S29. 10.1016/j.jacl.2014.02.012 24793439

[B138] Masana L. Pedro-Botet J. Civeira F. (2015). IMPROVE-IT clinical implications. Should the "high-intensity cholesterol-lowering therapy" strategy replace the "high-intensity statin therapy. Atherosclerosis 240 (1), 161–162. 10.1016/j.atherosclerosis.2015.03.002 25795557

[B139] Mauricio R. Khera A. (2022). Statin use in pregnancy: is it time for a paradigm shift? Circulation 145 (7), 496–498. 10.1161/CIRCULATIONAHA.121.058983 35157518

[B140] McKenney J. M. Davidson M. H. Jacobson T. A. Guyton J. R. National Lipid Association Statin Safety Assessment Task Force National Lipid Association Statin Safety Assessment Task, F (2006). Final conclusions and recommendations of the national lipid association statin safety assessment task force. Am. J. Cardiol. 97 (8A), 89C–94C. 10.1016/j.amjcard.2006.02.030 16581336

[B141] McKinney J. S. Kostis W. J. (2012). Statin therapy and the risk of intracerebral hemorrhage: a meta-analysis of 31 randomized controlled trials. Stroke 43 (8), 2149–2156. 10.1161/STROKEAHA.112.655894 22588266

[B142] Mehta A. Vasquez N. Ayers C. R. Patel J. Hooda A. Khera A. (2022). Independent association of lipoprotein(a) and coronary artery calcification with atherosclerotic cardiovascular risk. J. Am. Coll. Cardiol. 79 (8), 757–768. 10.1016/j.jacc.2021.11.058 35210030 PMC10966924

[B143] Mehta L. S. Warnes C. A. Bradley E. Burton T. Economy K. Mehran R. (2020). Cardiovascular considerations in caring for pregnant patients: a scientific statement from the American heart association. Circulation 141 (23), e884–e903. 10.1161/CIR.0000000000000772 32362133

[B144] Cholesterol Treatment Trialists Collaborators Mihaylova B. Emberson J. Blackwell L. Keech A. Simes J. Barnes E. H. (2012). The effects of lowering LDL cholesterol with statin therapy in people at low risk of vascular disease: meta-analysis of individual data from 27 randomised trials. Lancet 380 (9841), 581–590. 10.1016/S0140-6736(12)60367-5 22607822 PMC3437972

[B145] Moran A. Gu D. Zhao D. Coxson P. Wang Y. C. Chen C. S. (2010). Future cardiovascular disease in China: markov model and risk factor scenario projections from the coronary heart disease policy model-China. Circ. Cardiovasc Qual. Outcomes 3 (3), 243–252. 10.1161/CIRCOUTCOMES.109.910711 20442213 PMC2937540

[B146] Moriarty P. M. Thompson P. D. Cannon C. P. Guyton J. R. Bergeron J. Zieve F. J. (2015). Efficacy and safety of alirocumab vs ezetimibe in statin-intolerant patients, with a statin rechallenge arm: the ODYSSEY ALTERNATIVE randomized trial. J. Clin. Lipidol. 9 (6), 758–769. 10.1016/j.jacl.2015.08.006 26687696

[B147] Morrone D. Weintraub W. S. Toth P. P. Hanson M. E. Lowe R. S. Lin J. (2012). Lipid-altering efficacy of ezetimibe plus statin and statin monotherapy and identification of factors associated with treatment response: a pooled analysis of over 21,000 subjects from 27 clinical trials. Atherosclerosis 223 (2), 251–261. 10.1016/j.atherosclerosis.2012.02.016 22410123

[B148] Mozaffarian D. Aro A. Willett W. C. (2009). Health effects of trans-fatty acids: experimental and observational evidence. Eur. J. Clin. Nutr. 63 (2), S5–S21. 10.1038/sj.ejcn.1602973 19424218

[B149] Mozaffarian D. Micha R. Wallace S. (2010). Effects on coronary heart disease of increasing polyunsaturated fat in place of saturated fat: a systematic review and meta-analysis of randomized controlled trials. PLoS Med. 7 (3), e1000252. 10.1371/journal.pmed.1000252 20351774 PMC2843598

[B150] Nakamura H. Arakawa K. Itakura H. Kitabatake A. Goto Y. Toyota T. (2006). Primary prevention of cardiovascular disease with pravastatin in Japan (MEGA study): a prospective randomised controlled trial. Lancet 368 (9542), 1155–1163. 10.1016/S0140-6736(06)69472-5 17011942

[B151] National Center for Cardiovascular Diseases (2022). Report on cardiovascular health and diseases in China 2021 (in Chinese). Beijing: Science Press.

[B152] National Society of Cardiometabolic Medicine (2022). Chinese expert recommendations on lipid management options for community hospitals (2022 edition) (in Chinese). Chin. Circ. J. 37 (12), 1181–1185. 10.3969/j.issn.1000-3614.2022.12.002

[B153] Navarese E. P. Robinson J. G. Kowalewski M. Kolodziejczak M. Andreotti F. Bliden K. (2018). Association between baseline LDL-C level and total and cardiovascular mortality after LDL-C lowering: a systematic review and meta-analysis. JAMA 319 (15), 1566–1579. 10.1001/jama.2018.2525 29677301 PMC5933331

[B154] NCD Risk Factor Collaboration (2020). Repositioning of the global epicentre of non-optimal cholesterol. Nature 582 (7810), 73–77. 10.1038/s41586-020-2338-1 32494083 PMC7332422

[B155] Nishizaki Y. Miyauchi K. Iwata H. Inoue T. Hirayama A. Kimura K. (2022). Study protocol and baseline characteristics of randomized trial for evaluation in secondary prevention efficacy of combination therapy-statin and eicosapentaenoic acid: respect-epa, the combination of a randomized control trial and an observational biomarker study. Am. Heart J. 257, 1–8. 10.1016/j.ahj.2022.11.008 36372250

[B156] Nissen S. E. Nicholls S. J. Sipahi I. Libby P. Raichlen J. S. Ballantyne C. M. (2006). Effect of very high-intensity statin therapy on regression of coronary atherosclerosis: the ASTEROID trial. JAMA 295 (13), 1556–1565. 10.1001/jama.295.13.jpc60002 16533939

[B157] Nissen S. E. Stroes E. Dent-Acosta R. E. Rosenson R. S. Lehman S. J. Sattar N. (2016). Efficacy and tolerability of evolocumab vs ezetimibe in patients with muscle-related statin intolerance: the GAUSS-3 randomized clinical trial. JAMA 315 (15), 1580–1590. 10.1001/jama.2016.3608 27039291

[B158] Nissen S. E. Wolski K. Balog C. Swerdlow D. I. Scrimgeour A. C. Rambaran C. (2022). Single ascending dose study of a short interfering RNA targeting lipoprotein(a) production in individuals with elevated plasma lipoprotein(a) levels. JAMA 327 (17), 1679–1687. 10.1001/jama.2022.5050 35368052 PMC8978050

[B159] Norata G. D. Tibolla G. Catapano A. L. (2014). Targeting PCSK9 for hypercholesterolemia. Annu. Rev. Pharmacol. Toxicol. 54, 273–293. 10.1146/annurev-pharmtox-011613-140025 24160703

[B160] Nordestgaard B. G. Chapman M. J. Humphries S. E. Ginsberg H. N. Masana L. Descamps O. S. (2013). Familial hypercholesterolaemia is underdiagnosed and undertreated in the general population: guidance for clinicians to prevent coronary heart disease: consensus statement of the European atherosclerosis society. Eur. Heart J. 34 (45), 3478–390a. 10.1093/eurheartj/eht273 23956253 PMC3844152

[B161] Nordestgaard B. G. (2016). Triglyceride-rich lipoproteins and atherosclerotic cardiovascular disease: new insights from epidemiology, genetics, and biology. Circ. Res. 118 (4), 547–563. 10.1161/CIRCRESAHA.115.306249 26892957

[B162] Nordestgaard B. G. Varbo A. (2014). Triglycerides and cardiovascular disease. Lancet 384 (9943), 626–635. 10.1016/S0140-6736(14)61177-6 25131982

[B163] O'Donoghue M. L. Giugliano R. P. Wiviott S. D. Atar D. Keech A. Kuder J. F. (2022). Long-term evolocumab in patients with established atherosclerotic cardiovascular disease. Circulation 146 (15), 1109–1119. 10.1161/CIRCULATIONAHA.122.061620 36031810

[B164] Ong K. L. McClelland R. L. Allison M. A. Cushman M. Garg P. K. Tsai M. Y. (2021). Lipoprotein (a) and coronary artery calcification: prospective study assessing interactions with other risk factors. Metabolism 116, 154706. 10.1016/j.metabol.2021.154706 33421505 PMC7853621

[B165] Ouchi Y. Sasaki J. Arai H. Yokote K. Harada K. Katayama Y. (2019). Ezetimibe lipid-lowering trial on prevention of atherosclerotic cardiovascular disease in 75 or older (EWTOPIA 75): a randomized, controlled trial. Circulation 140 (12), 992–1003. 10.1161/CIRCULATIONAHA.118.039415 31434507

[B166] Pan L. Yang Z. Wu Y. Yin R. X. Liao Y. Wang J. (2016). The prevalence, awareness, treatment and control of dyslipidemia among adults in China. Atherosclerosis 248, 2–9. 10.1016/j.atherosclerosis.2016.02.006 26978581

[B167] Pedersen T. R. Faergeman O. Kastelein J. J. Olsson A. G. Tikkanen M. J. Holme I. (2005). High-dose atorvastatin vs usual-dose simvastatin for secondary prevention after myocardial infarction: the IDEAL study: a randomized controlled trial. JAMA 294 (19), 2437–2445. 10.1001/jama.294.19.2437 16287954

[B168] Phan B. A. Dayspring T. D. Toth P. P. (2012). Ezetimibe therapy: mechanism of action and clinical update. Vasc. Health Risk Manag. 8, 415–427. 10.2147/VHRM.S33664 22910633 PMC3402055

[B169] Qi L. Chen J. Li X. Qi X. Ding C. Chen X. (2022a). Efficacy and safety of hybutimibe in combination with atorvastatin for treatment of hypercholesteremia among patients with atherosclerotic cardiovascular disease risk equivalent: a multicenter, randomized, double-blinded phase III study. Front. Cardiovasc Med. 9, 888604. 10.3389/fcvm.2022.888604 36072875 PMC9443664

[B170] Qi L. Zhao S. Chen J. Zhang M. Li X. Dong Y. (2022b). Efficacy and safety of hybutimibe on primary hypercholesterolemia: a randomized, double-blinded, placebo and positive-controlled, parallel phase II study. Cardiol. Plus 7, 77–84. 10.1097/CP9.0000000000000012

[B171] Raal F. J. Hovingh G. K. Catapano A. L. (2018). Familial hypercholesterolemia treatments: guidelines and new therapies. Atherosclerosis 277, 483–492. 10.1016/j.atherosclerosis.2018.06.859 30270089

[B172] Raal F. J. Rosenson R. S. Reeskamp L. F. Hovingh G. K. Kastelein J. J. P. Rubba P. (2020). Evinacumab for homozygous familial hypercholesterolemia. N. Engl. J. Med. 383 (8), 711–720. 10.1056/NEJMoa2004215 32813947

[B173] Raal F. J. Santos R. D. Blom D. J. Marais A. D. Charng M. J. Cromwell W. C. (2010). Mipomersen, an apolipoprotein B synthesis inhibitor, for lowering of LDL cholesterol concentrations in patients with homozygous familial hypercholesterolaemia: a randomised, double-blind, placebo-controlled trial. Lancet 375 (9719), 998–1006. 10.1016/S0140-6736(10)60284-X 20227758

[B174] Ramaswami U. Humphries S. E. Priestley-Barnham L. Green P. Wald D. S. Capps N. (2019). Current management of children and young people with heterozygous familial hypercholesterolaemia - HEART UK statement of care. Atherosclerosis 290, 1–8. 10.1016/j.atherosclerosis.2019.09.005 31536851

[B175] Raposeiras-Roubin S. Rossello X. Oliva B. Fernandez-Friera L. Mendiguren J. M. Andres V. (2021). Triglycerides and residual atherosclerotic risk. J. Am. Coll. Cardiol. 77 (24), 3031–3041. 10.1016/j.jacc.2021.04.059 34140107 PMC8215641

[B176] Ray K. K. Bays H. E. Catapano A. L. Lalwani N. D. Bloedon L. T. Sterling L. R. (2019). Safety and efficacy of bempedoic acid to reduce LDL cholesterol. N. Engl. J. Med. 380 (11), 1022–1032. 10.1056/NEJMoa1803917 30865796

[B177] Ren J. Chen H. Luo Y. (2005). Efficacy and safety of combination therapy with simvastatin and fenofibrate for combined hyperlipidemia (in Chinese). Chin. J. Cardiol. 33 (2), 122–126. 10.3760/j:issn:0253-3758.2005.02.005 15924805

[B178] Ridker P. M. Danielson E. Fonseca F. A. Genest J. Gotto A. M. Jr. Kastelein J. J. (2008). Rosuvastatin to prevent vascular events in men and women with elevated C-reactive protein. N. Engl. J. Med. 359 (21), 2195–2207. 10.1056/NEJMoa0807646 18997196

[B179] Ridker P. M. Mora S. Rose L. Group J. T. S. (2016). Percent reduction in LDL cholesterol following high-intensity statin therapy: potential implications for guidelines and for the prescription of emerging lipid-lowering agents. Eur. Heart J. 37 (17), 1373–1379. 10.1093/eurheartj/ehw046 26916794 PMC4852064

[B180] Robinson J. G. Farnier M. Krempf M. Bergeron J. Luc G. Averna M. (2015). Efficacy and safety of alirocumab in reducing lipids and cardiovascular events. N. Engl. J. Med. 372 (16), 1489–1499. 10.1056/NEJMoa1501031 25773378

[B181] Roth E. M. Bays H. E. Forker A. D. Maki K. C. Carter R. Doyle R. T. (2009). Prescription omega-3 fatty acid as an adjunct to fenofibrate therapy in hypertriglyceridemic subjects. J. Cardiovasc Pharmacol. 54 (3), 196–203. 10.1097/FJC.0b013e3181b0cf71 19597368

[B182] Ruan Z. Jiang B. Chen J. Zhang X. Lou H. Xiang M. (2014). Pharmacokinetics, pharmacodynamics, safety, and tolerability of hyzetimibe (HS-25) in healthy Chinese subjects. J. Clin. Pharmacol. 54 (10), 1144–1152. 10.1002/jcph.310 24752831

[B183] Rubins H. B. Robins S. J. Collins D. Fye C. L. Anderson J. W. Elam M. B. (1999). Gemfibrozil for the secondary prevention of coronary heart disease in men with low levels of high-density lipoprotein cholesterol. Veterans Affairs High-Density Lipoprotein Cholesterol Intervention Trial Study Group. N. Engl. J. Med. 341 (6), 410–418. 10.1056/NEJM199908053410604 10438259

[B184] Sabatine M. S. De Ferrari G. M. Giugliano R. P. Huber K. Lewis B. S. Ferreira J. (2018). Clinical benefit of evolocumab by severity and extent of coronary artery disease: analysis from FOURIER. Circulation 138 (8), 756–766. 10.1161/CIRCULATIONAHA.118.034309 29626068

[B185] Sabatine M. S. Giugliano R. P. Keech A. C. Honarpour N. Wiviott S. D. Murphy S. A. (2017). Evolocumab and clinical outcomes in patients with cardiovascular disease. N. Engl. J. Med. 376 (18), 1713–1722. 10.1056/NEJMoa1615664 28304224

[B186] Sabatine M. S. Giugliano R. P. Wiviott S. D. Raal F. J. Blom D. J. Robinson J. (2015). Efficacy and safety of evolocumab in reducing lipids and cardiovascular events. N. Engl. J. Med. 372 (16), 1500–1509. 10.1056/NEJMoa1500858 25773607

[B187] Sacks F. M. Pfeffer M. A. Moye L. A. Rouleau J. L. Rutherford J. D. Cole T. G. (1996). The effect of pravastatin on coronary events after myocardial infarction in patients with average cholesterol levels. Cholesterol and Recurrent Events Trial investigators. N. Engl. J. Med. 335 (14), 1001–1009. 10.1056/NEJM199610033351401 8801446

[B188] Scandinavian Simvastatin Survival Study Group (1994). Randomised trial of cholesterol lowering in 4444 patients with coronary heart disease: the scandinavian simvastatin survival study (4S). Lancet 344 (8934), 1383–1389. 7968073

[B189] Schreml J. Gouni-Berthold I. (2018). Role of anti-PCSK9 antibodies in the treatment of patients with statin intolerance. Curr. Med. Chem. 25 (13), 1538–1548. 10.2174/0929867324666170616111647 28618994

[B190] Schwartz G. G. Olsson A. G. Ezekowitz M. D. Ganz P. Oliver M. F. Waters D. (2001). Effects of atorvastatin on early recurrent ischemic events in acute coronary syndromes: the MIRACL study: a randomized controlled trial. JAMA 285 (13), 1711–1718. 10.1001/jama.285.13.1711 11277825

[B191] Schwartz G. G. Steg P. G. Szarek M. Bhatt D. L. Bittner V. A. Diaz R. (2018). Alirocumab and cardiovascular outcomes after acute coronary syndrome. N. Engl. J. Med. 379 (22), 2097–2107. 10.1056/NEJMoa1801174 30403574

[B192] Serruys P. W. de Feyter P. Macaya C. Kokott N. Puel J. Vrolix M. (2002). Fluvastatin for prevention of cardiac events following successful first percutaneous coronary intervention: a randomized controlled trial. JAMA 287 (24), 3215–3222. 10.1001/jama.287.24.3215 12076217

[B193] Sever P. Gouni-Berthold I. Keech A. Giugliano R. Pedersen T. R. Im K. (2021). LDL-cholesterol lowering with evolocumab, and outcomes according to age and sex in patients in the FOURIER Trial. Eur. J. Prev. Cardiol. 28 (8), 805–812. 10.1177/2047487320902750 34298555

[B194] Sever P. S. Dahlof B. Poulter N. R. Wedel H. Beevers G. Caulfield M. (2003). Prevention of coronary and stroke events with atorvastatin in hypertensive patients who have average or lower-than-average cholesterol concentrations, in the anglo-scandinavian cardiac outcomes trial-lipid lowering arm (ASCOT-LLA): a multicentre randomised controlled trial. Lancet 361 (9364), 1149–1158. 10.1016/S0140-6736(03)12948-0 12686036

[B195] Sharifi M. Rakhit R. D. Humphries S. E. Nair D. (2016). Cardiovascular risk stratification in familial hypercholesterolaemia. Heart 102 (13), 1003–1008. 10.1136/heartjnl-2015-308845 27126396 PMC4941166

[B196] Sharp Collaborative Group (2010). Study of heart and renal protection (SHARP): randomized trial to assess the effects of lowering low-density lipoprotein cholesterol among 9,438 patients with chronic kidney disease. Am. Heart J. 160 (5), 785–794. 10.1016/j.ahj.2010.08.012 21095263

[B197] Shearer G. C. Pottala J. V. Hansen S. N. Brandenburg V. Harris W. S. (2012). Effects of prescription niacin and omega-3 fatty acids on lipids and vascular function in metabolic syndrome: a randomized controlled trial. J. Lipid Res. 53 (11), 2429–2435. 10.1194/jlr.P022392 22892157 PMC3466011

[B198] Sheng Y. Li M. Xu M. Zhang Y. Xu J. Huang Y. (2022). Left ventricular and atrial remodelling in hypertensive patients using thresholds from international guidelines and EMINCA data. Eur. Heart J. Cardiovasc Imaging 23 (2), 166–174. 10.1093/ehjci/jeab216 34718487

[B199] Shepherd J. Blauw G. J. Murphy M. B. Bollen E. L. Buckley B. M. Cobbe S. M. (2002). Pravastatin in elderly individuals at risk of vascular disease (PROSPER): a randomised controlled trial. Lancet 360 (9346), 1623–1630. 10.1016/s0140-6736(02)11600-x 12457784

[B200] Shepherd J. Cobbe S. M. Ford I. Isles C. G. Lorimer A. R. MacFarlane P. W. (1995). Prevention of coronary heart disease with pravastatin in men with hypercholesterolemia. West of Scotland Coronary Prevention Study Group. N. Engl. J. Med. 333 (20), 1301–1307. 10.1056/NEJM199511163332001 7566020

[B201] Silverman M. G. Ference B. A. Im K. Wiviott S. D. Giugliano R. P. Grundy S. M. (2016). Association between lowering LDL-C and cardiovascular risk reduction among different therapeutic interventions: a systematic review and meta-analysis. JAMA 316 (12), 1289–1297. 10.1001/jama.2016.13985 27673306

[B202] Skulas-Ray A. C. Wilson P. W. F. Harris W. S. Brinton E. A. Kris-Etherton P. M. Richter C. K. (2019). Omega-3 fatty acids for the management of hypertriglyceridemia: a science advisory from the American heart association. Circulation 140 (12), e673–e691. 10.1161/CIR.0000000000000709 31422671

[B203] Sniderman A. D. Williams K. Contois J. H. Monroe H. M. McQueen M. J. de Graaf J. (2011). A meta-analysis of low-density lipoprotein cholesterol, non-high-density lipoprotein cholesterol, and apolipoprotein B as markers of cardiovascular risk. Circ. Cardiovasc Qual. Outcomes 4 (3), 337–345. 10.1161/CIRCOUTCOMES.110.959247 21487090

[B204] Song P. K. Man Q. Q. Li H. Pang S. J. Jia S. S. Li Y. Q. (2019). Trends in lipids level and dyslipidemia among Chinese adults, 2002-2015. Biomed. Environ. Sci. 32 (8), 559–570. 10.3967/bes2019.074 31488232

[B205] Stein E. A. Turner T. A. (2017). Are the PCSK9 inhibitors the panacea of atherosclerosis treatment? Expert Rev. Cardiovasc Ther. 15 (7), 491–494. 10.1080/14779072.2017.1348231 28651464

[B206] Stroes E. S. Thompson P. D. Corsini A. Vladutiu G. D. Raal F. J. Ray K. K. (2015). Statin-associated muscle symptoms: impact on statin therapy-European atherosclerosis society consensus Panel statement on assessment, aetiology and management. Eur. Heart J. 36 (17), 1012–1022. 10.1093/eurheartj/ehv043 25694464 PMC4416140

[B207] Sun D. Zhou B. Y. Li S. Sun N. L. Hua Q. Wu S. L. (2018). Genetic basis of index patients with familial hypercholesterolemia in Chinese population: mutation spectrum and genotype-phenotype correlation. Lipids Health Dis. 17 (1), 252. 10.1186/s12944-018-0900-8 30400955 PMC6220500

[B208] Tanasescu M. Cho E. Manson J. E. Hu F. B. (2004). Dietary fat and cholesterol and the risk of cardiovascular disease among women with type 2 diabetes. Am. J. Clin. Nutr. 79 (6), 999–1005. 10.1093/ajcn/79.6.999 15159229

[B209] Thanassoulis G. Williams K. Ye K. Brook R. Couture P. Lawler P. R. (2014). Relations of change in plasma levels of LDL-C, non-HDL-C and apoB with risk reduction from statin therapy: a meta-analysis of randomized trials. J. Am. Heart Assoc. 3 (2), e000759. 10.1161/JAHA.113.000759 24732920 PMC4187506

[B210] The Editorial Board of Chinese Journal of PediatricsThe Subspecialty Group of Child Health Care, T.S.o.P.Chinese Medical AssociationThe Subspecialty Group of Cardiovascular Disease, T.S.o.P.Chinese Medical AssociationThe Subspecialty Group of Atherosclerosis, T.S.o.C.D.Chinese Medical Association (2009). Experts consensus for prevention and treatment of dyslipidemia in children and adolescents (in Chinese). Chin. J. Pediatr. 47 (6), 426–428. 10.3760/cma.j.issn.0578-1310.2009.06.007 19951468

[B211] The Medical Letter (2021). Evinacumab (Evkeeza) for homozygous familial hypercholesterolemia. Med. Lett. Drugs Ther. 63 (1623), 66–67. 1523-2859 (Electronic). 33976097

[B212] The Subspecialty Group of Rare DiseasesT.S.o.P., Chinese Medical AssociationThe Subspecialty Group of Cardiology, T.S.o.P.Chinese Medical AssociationThe Subspecialty Group of Child Health Care, T.S.o.P.Chinese Medical Association, The Subspecialty Group of Endocrinological, H.a.M.D.The Society of PediatricsChinese Medical Association, and The Editorial Board, C.J.o.P. (2022). Expert consensus on diagnosis and management of dyslipidemia in children (in Chinese). Chin. J. Pediatr. 20 (7), 633–639. 10.3760/cma.j.cn112140-20211108-00936 35768349

[B213] Thedrez A. Blom D. J. Ramin-Mangata S. Blanchard V. Croyal M. Chemello K. (2018). Homozygous familial hypercholesterolemia patients with identical mutations variably express the LDLR (Low-Density lipoprotein receptor): implications for the efficacy of evolocumab. Arterioscler. Thromb. Vasc. Biol. 38 (3), 592–598. 10.1161/ATVBAHA.117.310217 29284604 PMC5823753

[B214] Tsimikas S. Karwatowska-Prokopczuk E. Gouni-Berthold I. Tardif J. C. Baum S. J. Steinhagen-Thiessen E. (2020). Lipoprotein(a) reduction in persons with cardiovascular disease. N. Engl. J. Med. 382 (3), 244–255. 10.1056/NEJMoa1905239 31893580

[B215] Vahedian-Azimi A. Bianconi V. Makvandi S. Banach M. Mohammadi S. M. Pirro M. (2021a). A systematic review and meta-analysis on the effects of statins on pregnancy outcomes. Atherosclerosis 336, 1–11. 10.1016/j.atherosclerosis.2021.09.010 34601188

[B216] Vahedian-Azimi A. Makvandi S. Banach M. Reiner Z. Sahebkar A. (2021b). Fetal toxicity associated with statins: a systematic review and meta-analysis. Atherosclerosis 327, 59–67. 10.1016/j.atherosclerosis.2021.05.006 34044205

[B217] Vallejo-Vaz A. J. Fayyad R. Boekholdt S. M. Hovingh G. K. Kastelein J. J. Melamed S. (2018). Triglyceride-rich lipoprotein cholesterol and risk of cardiovascular events among patients receiving statin therapy in the TNT trial. Circulation 138 (8), 770–781. 10.1161/CIRCULATIONAHA.117.032318 29618599

[B218] Vallejo-Vaz A. J. Kondapally Seshasai S. R. Kurogi K. Michishita I. Nozue T. Sugiyama S. (2015). Effect of pitavastatin on glucose, HbA1c and incident diabetes: a meta-analysis of randomized controlled clinical trials in individuals without diabetes. Atherosclerosis 241 (2), 409–418. 10.1016/j.atherosclerosis.2015.06.001 26074315

[B219] Venero C. V. Venero J. V. Wortham D. C. Thompson P. D. (2010). Lipid-lowering efficacy of red yeast rice in a population intolerant to statins. Am. J. Cardiol. 105 (5), 664–666. 10.1016/j.amjcard.2009.10.045 20185013

[B220] Vuorio A. Kuoppala J. Kovanen P. T. Humphries S. E. Tonstad S. Wiegman A. (2019). Statins for children with familial hypercholesterolemia. Cochrane Database Syst. Rev. 2019 (11). 10.1002/14651858.CD006401.pub5 PMC6836374 31696945

[B221] Wang Y. Feng L. Zeng G. Zhu H. Sun J. Gao P. (2022). Effects of cuisine-based Chinese heart-healthy diet in lowering blood pressure among adults in China: multicenter, single-blind, randomized, parallel controlled feeding trial. Circulation 146 (4), 303–315. 10.1161/CIRCULATIONAHA.122.059045 35861850 PMC9311470

[B222] Wang Z. Zou Z. Yang Y. Wang S. Dong Y. Yang Z. (2018). The epidemiological characteristics and related factors of dyslipidemia among children and adolescents aged 6-17 years from 7 provinces in China, 2012. Chin. J. Prev. Med. 52 (8), 798–801. 10.3760/cma.j.issn.0253-9624.2018.08.005 30107712

[B223] Wanner C. Krane V. Marz W. Olschewski M. Mann J. F. Ruf G. (2005). Atorvastatin in patients with type 2 diabetes mellitus undergoing hemodialysis. N. Engl. J. Med. 353 (3), 238–248. 10.1056/NEJMoa043545 16034009

[B224] Watts G. F. Sullivan D. R. Hare D. L. Kostner K. M. Horton A. E. Bell D. A. (2021). Integrated guidance for enhancing the care of familial hypercholesterolaemia in Australia. Heart Lung Circ. 30 (3), 324–349. 10.1016/j.hlc.2020.09.943 33309206

[B225] Wiggins B. S. Saseen J. J. Page R. L. 2nd Reed B. N. Sneed K. Kostis J. B. (2016). Recommendations for management of clinically significant drug-drug interactions with statins and select agents used in patients with cardiovascular disease: a scientific statement from the American heart association. Circulation 134 (21), e468–e495. 10.1161/CIR.0000000000000456 27754879

[B226] Willeit P. Kiechl S. Kronenberg F. Witztum J. L. Santer P. Mayr M. (2014). Discrimination and net reclassification of cardiovascular risk with lipoprotein(a): prospective 15-year outcomes in the bruneck study. J. Am. Coll. Cardiol. 64 (9), 851–860. 10.1016/j.jacc.2014.03.061 25169167

[B227] Willer C. J. Schmidt E. M. Sengupta S. Peloso G. M. Gustafsson S. Kanoni S. (2013). Discovery and refinement of loci associated with lipid levels. Nat. Genet. 45 (11), 1274–1283. 10.1038/ng.2797 24097068 PMC3838666

[B228] Willerson J. T. (1996). Effect of pravastatin on coronary events after myocardial infarction in patients with average cholesterol levels. Circulation 94 (12), 3054. 10.1161/01.cir.94.12.3054 8989104

[B229] Wilson D. P. Jacobson T. A. Jones P. H. Koschinsky M. L. McNeal C. J. Nordestgaard B. G. (2019). Use of lipoprotein(a) in clinical practice: a biomarker whose time has come. A scientific statement from the national lipid association. J. Clin. Lipidol. 13 (3), 374–392. 10.1016/j.jacl.2019.04.010 31147269

[B230] Witztum J. L. Gaudet D. Freedman S. D. Alexander V. J. Digenio A. Williams K. R. (2019). Volanesorsen and triglyceride levels in familial chylomicronemia syndrome. N. Engl. J. Med. 381 (6), 531–542. 10.1056/NEJMoa1715944 31390500

[B231] Wu J. Wang J. Douglas R. (2017). Effect of paranasal anatomical variants on outcomes in patients with limited and diffuse chronic rhinosinusitis. Chin. J. Lab. Med. 40 (6), 417–421. 10.1016/j.anl.2016.08.009 27614778

[B232] Wu Y. Liu X. Li X. Li Y. Zhao L. Chen Z. (2006). Estimation of 10-year risk of fatal and nonfatal ischemic cardiovascular diseases in Chinese adults. Circulation 114 (21), 2217–2225. 10.1161/CIRCULATIONAHA.105.607499 17088464

[B233] Xie W. Liang L. Zhao L. Shi P. Yang Y. Xie G. (2011). Combination of carotid intima-media thickness and plaque for better predicting risk of ischaemic cardiovascular events. Heart 97 (16), 1326–1331. 10.1136/hrt.2011.223032 21653216

[B234] Xu D. Hu J. Wu Q. Du Z. Xue Y. Zhang X. (2018). Efficacy and safety of Zhibitai in combination with atorvastatin for lipid lowering in patients with coronary heart disease. Oncotarget 9 (10), 9489–9497. 10.18632/oncotarget.18329 29507705 PMC5823641

[B235] Xu D. Shu J. Huang Q. Liu L. Zhao S. (2010a). Comparative study of the efficacy and safety of Zhibitai and atorvastatin (in Chinese). Chin. J. Intern Med. 49 (5), 392–395. 10.3760/cma.j.issn.0578-1426.2010.05.008 20646412

[B236] Xu D. Shu J. Huang Q. Y. Wasti B. Chen C. Liu L. (2010b). Evaluation of the lipid lowering ability, anti-inflammatory effects and clinical safety of intensive therapy with Zhibitai, a Chinese traditional medicine. Atherosclerosis 211 (1), 237–241. 10.1016/j.atherosclerosis.2010.01.044 20189174

[B237] Xu R. X. Li S. Li X. L. Zhang Y. Guo Y. L. Zhu C. G. (2015). High-density lipoprotein subfractions in relation with the severity of coronary artery disease: a gensini score assessment. J. Clin. Lipidol. 9 (1), 26–34. 10.1016/j.jacl.2014.11.003 25670357

[B238] Yamashita S. Bujo H. Arai H. Harada-Shiba M. Matsui S. Fukushima M. (2008). Long-term probucol treatment prevents secondary cardiovascular events: a cohort study of patients with heterozygous familial hypercholesterolemia in Japan. J. Atheroscler. Thromb. 15 (6), 292–303. 10.5551/jat.e610 19060422

[B239] Yan S. (2003). Recommendations for clinical lipid profile testing (in Chinese). Chin. J. Lab. Med. 26 (3), 182–184. 10.3760/j:issn:1009-9158.2003.03.019

[B240] Yan S. (2008). The link between lipid profile testing and clinical practice should Be further strengthened (in Chinese). Chin. J. Clin. Lab. Sci. 26 (4), 243–245. 10.13602/j.cnki.jcls.2008.04.001

[B241] Yang W. Xiao J. Yang Z. Ji L. Jia W. Weng J. (2012). Serum lipids and lipoproteins in Chinese men and women. Circulation 125 (18), 2212–2221. 10.1161/CIRCULATIONAHA.111.065904 22492668

[B242] Yang X. Li J. Hu D. Chen J. Li Y. Huang J. (2016). Predicting the 10-year risks of atherosclerotic cardiovascular disease in Chinese population: the China-par project (prediction for ASCVD risk in China). Circulation 134 (19), 1430–1440. 10.1161/CIRCULATIONAHA.116.022367 27682885

[B243] Yang Y. Li J. Sun Y. (2022). Views on FDA′s withdrawal of strongest warning against using cholesterol-lowering statins during pregnancy (in Chinese). Chin. J. Cardiol. 50 (9), 851–852. 10.3760/cma.j.cn112148-20220402-00235 36096701

[B244] Yeboah J. Young R. McClelland R. L. Delaney J. C. Polonsky T. S. Dawood F. Z. (2016). Utility of nontraditional risk markers in atherosclerotic cardiovascular disease risk assessment. J. Am. Coll. Cardiol. 67 (2), 139–147. 10.1016/j.jacc.2015.10.058 26791059 PMC4724058

[B245] Yokoyama M. Origasa H. Matsuzaki M. Matsuzawa Y. Saito Y. Ishikawa Y. (2007). Effects of eicosapentaenoic acid on major coronary events in hypercholesterolaemic patients (JELIS): a randomised open-label, blinded endpoint analysis. Lancet 369 (9567), 1090–1098. 10.1016/S0140-6736(07)60527-3 17398308

[B246] Yusuf S. Bosch J. Dagenais G. Zhu J. Xavier D. Liu L. (2016a). Cholesterol lowering in intermediate-risk persons without cardiovascular disease. N. Engl. J. Med. 374 (21), 2021–2031. 10.1056/NEJMoa1600176 27040132

[B247] Yusuf S. Lonn E. Pais P. Bosch J. Lopez-Jaramillo P. Zhu J. (2016b). Blood-pressure and cholesterol lowering in persons without cardiovascular disease. N. Engl. J. Med. 374 (21), 2032–2043. 10.1056/NEJMoa1600177 27039945

[B248] Zeng Y. Liu J. Liu J. Hao Y. Yang N. Zhou M. (2020). The expanding needs on lipid-lowering treatment in patients with acute coronary syndrome by applying newly issued definition of extreme high-risk by Chinese society of Cardiology (in Chinese). Chin. J. Cardiol. 48 (12), 1039–1046. 10.3760/cma.j.cn112148-20200710-00549 33355748

[B249] Zhang H. Hu L. Wei X. (2020). Prognostic value of left ventricular hypertrophy in hypertensive patients: a meta-analysis of electrocardiographic studies. J. Clin. Hypertens. (Greenwich) 22 (2), 254–260. 10.1111/jch.13795 31955500 PMC8030042

[B250] Zhang J. Peng H. Li G. Su Z. Li P. Wang Z. (2022). Clinical value of remnant lipoproteins and low-density lipoprotein cholesterol particle concentration detected by vertical auto profile on the diagnosis of carotid plaque (in Chinese). Chin. J. Lab. Med. 45 (6), 575–581. 10.3760/cma.j.cn114452-20220109-00020

[B251] Zhang M. Deng Q. Wang L. Huang Z. Zhou M. Li Y. (2018). Prevalence of dyslipidemia and achievement of low-density lipoprotein cholesterol targets in Chinese adults: a nationally representative survey of 163,641 adults. Int. J. Cardiol. 260, 196–203. 10.1016/j.ijcard.2017.12.069 29622441

[B252] Zhao D. Liu J. Wang M. Zhang X. Zhou M. (2019). Epidemiology of cardiovascular disease in China: current features and implications. Nat. Rev. Cardiol. 16 (4), 203–212. 10.1038/s41569-018-0119-4 30467329

[B253] Zhao L. Gao Y. Liu G. Jia C. Zhang J. Dong Q. (2022). Lipoprotein apheresis in patients with familial hypercholesterolemia: a single center research (in Chinese). Chin. J. Cardiol. 50 (6), 585–590. 10.3760/cma.j.cn112148-20210715-00591 35705468

[B254] Zhao L. Wen J. Guo Y. (2021). Research progress of liver transplantation for patients with homozygous familial hypercholesterolemia (in Chinese). Chin. J. Arterioscler. 29 (4), 353–358. 10.3969/j.issn.1007-3949.2021.04.015

[B255] Zhao S. (2003). Lipids lecture 4: clinical manifestations and types of hyperlipidemia (in Chinese). Chin. J. Clin. 31 (12), 23–24. 10.3969/j.issn.1008-1089.2003.12.013

[B256] Zhao S. P. Liu L. Cheng Y. C. Shishehbor M. H. Liu M. H. Peng D. Q. (2004). Xuezhikang, an extract of cholestin, protects endothelial function through antiinflammatory and lipid-lowering mechanisms in patients with coronary heart disease. Circulation 110 (8), 915–920. 10.1161/01.CIR.0000139985.81163.CE 15313947

[B257] Zhao S. P. Yu B. L. Peng D. Q. Huo Y. (2014). The effect of moderate-dose versus double-dose statins on patients with acute coronary syndrome in China: results of the CHILLAS trial. Atherosclerosis 233 (2), 707–712. 10.1016/j.atherosclerosis.2013.12.003 24603217

[B258] Zhao S. Wang F. Dai Y. Lin L. Tong Q. Liao Y. (2016). Efficacy and safety of fenofibrate as an add-on in patients with elevated triglyceride despite receiving statin treatment. Int. J. Cardiol. 221, 832–836. 10.1016/j.ijcard.2016.06.234 27434354

[B259] Zhao Y. Peng R. Zhao W. Liu Q. Guo Y. Zhao S. (2017). Zhibitai and low-dose atorvastatin reduce blood lipids and inflammation in patients with coronary artery disease. Med. Baltim. 96 (7), e6104. 10.1097/MD.0000000000006104 PMC5319516 28207527

[B260] Zhong V. W. Van Horn L. Cornelis M. C. Wilkins J. T. Ning H. Carnethon M. R. (2019). Associations of dietary cholesterol or egg consumption with incident cardiovascular disease and mortality. JAMA 321 (11), 1081–1095. 10.1001/jama.2019.1572 30874756 PMC6439941

