# Supplementary material for: 2023 Chinese guideline for lipid management
Source: Front Pharmacol. 2023 Aug 29;14:1190934. doi: 10.3389/fphar.2023.1190934 (PMC10498001; doi:10.3389/fphar.2023.1190934)
Supplement: Supplementary file 1 [file DataSheet1.pdf]

Supplementary documents

Table S1 Core Issues of the 2023 Guidelines

**Core Issues Solicited from the Expert Committee Members**
Confirmation of writing patterns: Whether the revision includes recommended classifications and levels of evidence, and corresponding supporting evidence in accordance with the standards of international guidelines?
Whether the title needs to be revised?
The statement of whether cholesterol is a causative risk factor for ASCVD needed to be emphasized?
Routine blood lipid testing items, methods and the difference between fasting and non-fasting blood lipids?
How to recommend LDL-C goal and non-HDL-C goal?
ASCVD hazard stratification criteria?
Do LDL-C/non-HDL-C goal values for different populations need to be listed with normal values?
What are the interventions and treatment options for high TG?
Do a recommended limit needed to be added on dietary cholesterol intake?
Medication for Chinese: Statin Dose? Combination non-statin? Evidence of Traditional Chinese Medicine?
Lipid intervention for special populations? FH, CKD, elderly, etc.
How often are blood lipids and safety indicators monitored after drug treatment?

ASCVD: atherosclerotic cardiovascular disease; LDL-C: low-density lipoprotein cholesterol; HDL-C: high-density lipoprotein cholesterol; TC: total cholesterol; FH: familial hypercholesterolemia; CKD: chronic kidney disease

Table S2 New Update 2023 China Guidelines for the Management of Blood Lipids Compared to 2016 Version

2016	2023
1.Blood Lipids and Lipoproteins	1.Epidemic Characteristics of Dyslipidemia
2.Blood Lipid Testing Programs	2.Lipids and Lipoproteins
3.Appropriate Blood Lipid Levels and Abnormal Cut-off Points	3.Lipid Testing Programs
4. Classification of Dyslipidemia	4.Overall Risk Assessment of Atherosclerotic Cardiovascular Disease
5.Dyslipidemia Screening	5.Reference Standard for Appropriate Blood Lipid Levels
6.Overall Cardiovascular Risk Assessment	6.Classification of Dyslipidemia
7.Principles of Treatment for Dyslipidemia	7.Blood Lipid Screening
8.Therapeutic Lifestyle Changes	8.Principles of Dyslipidemia Treatment
9.Lipid Lowering Drug Treatment	9.Lipid-lowering Drug Therapy
10.Other Measures of Dyslipidemia Treatment	10.Other Measures of Lipid Lowering Treatment
11.Management of Dyslipidemia in Special Population	11.Lipid Management for Specific Populations

Table S3 Physical and biological features and functions of lipoproteins

Category	Density (g/ml)	Diameter (nm)	Main lipid components (%)				Apoliprotein		Main source	Function
			TG	Cholesteryl-ester	Phospho-lipid	Cholesterol	Main	Other		
CM	＜ 0.950	80～100	90～95	2～4	2～6	1	B48	A1, A2, A4, A5	Synthesized by the small intestine	Transport of TG and cholesterol from food from the small intestine to other tissues
VLDL	0.950～1.006	30～80	50～65	8～14	12～16	4～7	B100	A1, C2, C3, E, A5	Synthesized by the liver	Transport of endogenous TG to peripheral tissues and release of free fatty acids after hydrolysis by lipase
IDL	1.006～1.019	25～30	25～40	20～35	16～24	7～11	B100	C2, C3, E	TG in VLDL is formed after hydrolysis by lipase	LDL precursor, partially metabolized by the liver
LDL	1.019～1.063	20～25	4～6	34～35	22～26	6～15	B100		TG in VLDL and IDL is formed after hydrolysis by lipase	The main carrier of cholesterol, mediated by LDL receptors and taken up and utilized by peripheral tissues
HDL	1.063～1.210	8～13	7	10～20	55	5	A1	A2, C3, E, M	Mainly synthesized by the liver and small intestine	Facilitates cholesterol removal from peripheral tissues and transports cholesterol to the liver or other tissues for redistribution
Lp (a)	1.055～1.085	25～30	4～8	35～46	17～24	6～9	Apo(a)	B100	Complex formation of Apo(a) with LDL via disulfide bonds in the liver or extra-hepatically	Function has not been fully understood yet.

CM, chylomicron; VLDL, very low-density lipoprotein; IDL, intermediate-density lipoprotein; LDL, low-density lipoprotein; HDL, high-density lipoprotein; Lp(a), lipoprotein (a)

Table S4 Recommended lipid target values for diabetic patients AND Lipid-lowering therapy in patients with CKD stage 3 to 5

Recommendation	Recommended classification	Evidence level
**Recommended lipid target values for diabetic patients**		
Patients with diabetes combined with ASCVD, LDL-C <1.4 mmol/L (Cannon et al., 2015; Sabatine et al., 2017; Schwartz et al., 2018)	I	A
Patients with diabetes at high risk for ASCVD: LDL-C < 1.8 mmol/L (Cholesterol Treatment Trialists Collaborators, 2008)	I	A
Patients with diabetes at low and moderate risk for ASCVD: LDL-C < 2.6 mmol/L (Bhatt et al., 2019)	IIa	C
Diabetic patients with non-HDL-C as a secondary target with corresponding LDL-C target values + 0.8 mmol/L	I	C
**Lipid-lowering therapy in patients with CKD stage 3 to 5**		
For non-dialysis dependent patients with CKD stage 3 to 5, statins or statins combined with cholesterol absorption inhibitors are recommended to lower LDL-C (Baigent et al., 2011; Cholesterol Treatment Trialists Collaboration, 2016)	I	A
For patients with ASCVD combined with CKD stages 3	IIa	C

to 5 who are already receiving statins or statins in combination with cholesterol absorption inhibitors, consider continuing these drugs when starting dialysis treatment (Baigent et al., 2011; Cholesterol Treatment Trialists Collaboration, 2016)		
For non-ASCVD patients dependent on dialysis, statins are not recommended (Wanner et al., 2005; Fellstrom et al., 2009).	III	A

ASCVD: atherosclerotic cardiovascular disease; LDL-C: low-density lipoprotein cholesterol; HDL-C: high-density lipoprotein cholesterol. a Patients at high risk for ASCVD refer to patients with diabetes mellitus aged ≥40 years, and patients aged 20 to 39 years with diabetes combined with ≥3 risk factors, or target organ damage, or type 1 diabetes with a duration >20 years can be considered at high risk for ASCVD. Major risk factors: hypertension, dyslipidemia, smoking, obesity, family history of early-onset coronary heart disease. Target organ damage: proteinuria, renal impairment, left ventricular hypertrophy, or retinopathy (refer to risk stratification section); CKD: chronic kidney disease

Table S5 Lipid-lowering therapy in patients with stroke AND Lipid-lowering regimen options for elder people ≥75 years old

Recommendation	Recommended classification	Evidence level
**Lipid-lowering therapy in patients with stroke**		
For patients with atherosclerotic ischemic stroke or TIA combined with definite CAD or PAD, LDL-C <1.4 mmol/L; non-HDL-C <2.2 mmol/L is recommended (Cannon et al., 2015; Sabatine et al., 2017; Schwartz et al., 2018)	I	A
For patients with pure atherosclerotic ischemic stroke or TIA, LDL-C <1.8 mmol/L; non-HDL-C <2.6 mmol/L is recommended (Amarenco et al., 2006; Amarenco and Labreuche, 2009)	I	A
For atherosclerotic ischemic stroke or TIA, statins are recommended as the treatment of choice (Amarenco et al., 2006; Amarenco and Labreuche, 2009)	I	A
For atherosclerotic ischemic stroke or TIA, cholesterol absorption inhibitors can be added for those who do not meet LDL-C targets with statins (Cannon et al., 2015)	IIa	B
For atherosclerotic ischemic stroke or TIA, statins + cholesterol absorption inhibitors may be added to PCSK9 inhibitors for those who do not achieve LDL-C targets (Sabatine et al., 2017; Schwartz et al., 2018)	IIa	B
**Lipid-lowering regimen options for elder people ≥75 years old**		
Lipid-lowering therapy is recommended for patients ≥75 years with combined ASCVD (Shepherd et al., 2002; Sabatine et al., 2017; Schwartz et al., 2018; Bach et al., 2019; Cholesterol Treatment Trialists Collaboration, 2019; Hypertensive Group of Chinese Society of Cardiology of Chinese Medical Association and Editorial Board of Chinese Journal of Cardiology, 2021)	IIa	B
For people aged ≥75 years at high risk for ASCVD, co-morbidities, frailty, life expectancy and their willingness need to be taken into account, and if the benefits outweigh the risks, initiation of statin therapy for primary prevention	IIb	B

is recommended (Shepherd et al., 2002; Cholesterol Treatment Trialists Collaboration, 2019)		
For people ≥75 years of age at high risk for ASCVD, co-morbidities, frailty, life expectancy, and their willingness need to be taken into account, and initiation of cholesterol absorption inhibitor therapy is recommended for primary prevention (Shepherd et al., 2002; Cholesterol Treatment Trialists Collaboration, 2019)	IIa	B
For people ≥75 years of age, it is recommended to start with low-dose statins if there are potential drug interactions or renal impairment, and to consider combination cholesterol absorption inhibitors or PCSK9 inhibitors for those who cannot reach the target at moderate doses (Sever et al., 2021)	IIb	C

TIA: transient ischemic attack; CAD: coronary artery disease; PAD: peripheral vascular disease; LDL-C: low-density lipoprotein cholesterol; HDL-C: high-density lipoprotein cholesterol; PCSK9: preprotein converting enzyme subtilisin/kexin 9; ASCVD: atherosclerotic cardiovascular disease

Table S6 Reference standards for dyslipidemia in children and adolescents (mmol/L) AND FH diagnosis and treatment recommendations

Reference standards for dyslipidemia in children and adolescents (mmol/L)			
**Blood lipid**	**Appropriate**	**Critical increase/decrease**	**Abnormal**
TC	< 4.4	4.4 ~ 5.2	≥5.2
LDL-C	< 2.8	2.8 ~ 3.4	≥3.4
TG < 10 years of age	<0.8	0.8 ~ 1.1	≥1.1
≥10 years of age	< 1.0	1.0 ~ 1.5	≥1.5
HDL-C	≥1.2	1.0 ~ 1.2	< 1.0
Non-HDL-C	< 3.1	3.1 ~ 3.7	≥3.7
**FH diagnosis and treatment recommendations**			
Both clinical phenotypic diagnosis and genetic diagnosis can be used for FH screening and diagnosis, with the former being the basis for the latter; genetic diagnosis can help confirm FH diagnosis and family screening, but the absence of pathogenic mutations cannot exclude FH (Atherosclerosis and Coronary Heart Disease Group of the Chinese Society of Cardiology of Chinese Medical Association and Editorial Board of Chinese Journal of Cardiology, 2018; Lee et al., 2019; Di Taranto et al., 2021; Watts et al., 2021)		I	B
LDL-C target values for adult patients with FH (Watts et al., 2021): <2.6 mmol/L without ASCVD; <1.8 mmol/L with subclinical ASCVD; <1.4 mmol/L with clinical ASCVD		IIa	B

LDL-C target values for children and adolescents with FH (<18 years old) (Harada-Shiba et al., 2018b; Ramaswami et al., 2019; Vuorio et al., 2019; Watts et al., 2021): without ASCVD <3.5 mmol/L or ≥50% reduction from baseline; with subclinical ASCVD <2.6 mmol/L and ≥50% reduction from baseline; with clinical ASCVD <1.8 mmol/L and ≥50% reduction from baseline	IIa	C
Single or combination LDL-C-lowering drugs, including statins, cholesterol absorption inhibitors, PCSK9 inhibitors, etc., are selected according to LDL-C compliance needs and individual tolerances	I	A
HoFH patients can be combined with Mipomersen, Lomitapide, Evinacumab (all approved abroad)	I	B
Maximum tolerated dose drug therapy for HoFH or severe phenotype HeFH patients with suboptimal LDL-C is recommended in combination with lipoprotein apheresis therapy at least every 2 weeks	I	C
Children with suspected HeFH should be diagnosed as early as possible (no later than 10 years of age); initiation of statin therapy is recommended for diagnosed individuals with LDL-C ≥4.7 mmol/L twice after lifestyle intervention (≥8 years of age); combination of cholesterol absorption inhibitors may be indicated after statin therapy ≥4.0 mmol/L (≥10 years of age) (Harada-Shiba et al., 2018b; Luirink	I	B

et al., 2019; Ramaswami et al., 2019; Vuorio et al., 2019; Watts et al., 2021)		
Children with suspected HoFH should be diagnosed as early as possible (preferably before 2 years of age); those diagnosed should start statins combined with cholesterol absorption inhibitors as early as possible (preferably before 2 years of age); start lipoprotein apheresis as early as possible (preferably before 5 years of age and no later than 8 years of age), once every 1-2 weeks; ≥12 years of age can be combined with PCSK9 monoclonal antibody, Mipomersen or Evinacumab (Harada-Shiba et al., 2018b; Luirink et al., 2019; Vuorio et al., 2019; The Medical Letter, 2021; Watts et al., 2021)	I	B
Young patients with severe phenotype HoFH who have poor drug efficacy and cannot be treated with regular lipoprotein apheresis may be considered for liver transplantation prior to cardiovascular involvement; those who have developed rapidly progressive ASCVD or severe aortic stenosis should be considered for combined heart-liver transplantation (Raal et al., 2018; Ishigaki et al., 2019; Watts et al., 2021; Zhao et al., 2021)	IIb	C

TC: total cholesterol; LDL-C: low-density lipoprotein cholesterol; TG: triglycerides; HDL-C: high-density lipoprotein cholesterol; FH: familial hypercholesterolemia; LDL-C: low-density lipoprotein cholesterol; ASCVD: atherosclerotic cardiovascular disease; PCSK9: preprotein converting enzyme subtilisin/kexin 9; HoFH: homozygous FH; HeFH: heterozygous FH.
